# Effects of tactical dimension and situational variables in throw-ins on the offensive performance in football

**DOI:** 10.1371/journal.pone.0294317

**Published:** 2023-11-15

**Authors:** Claudio A. Casal, Vasilis Armatas, José Luis Losada, Michalis Mitrotasios

**Affiliations:** 1 Department of Science of Physical Activity and Sport, Catholic University of Valencia, Valencia, Spain; 2 School of Physical Education & Sport Science, National & Kapodistrian University of Athens, Athens, Greece; 3 Department of Social Psychology and Quantitative Psychology, University of Barcelona, Barcelona, Spain; Instituto Politécnico de Santarém: Instituto Politecnico de Santarem, PORTUGAL

## Abstract

The aim of the study was to describe the usual practices in the execution of throw-ins by La Liga teams during the 2021–2022 season, identify tactical indicators related to the outcome of plays that start with a throw-in, calculate their predictive power, and finally analyse the influence of situational variables on the effectiveness of these plays. A total of 2,658 throw-ins, during 80 matches were analysed. Two UEFA PRO coaches designed an *ad hoc* observation instrument “*Thrinfoot*” and two observers coded the data after a training process. Inter and intra-observer reliability was calculated using Cohen´s Kappa coefficient, revealing almost perfect agreement. Multinomial logistic regression was applied to predict the throw-ins outcome (*p*<0.05). Results showed how fast throw-ins (OR = 0.7, p<0.05), without pressing (OR = 0.4, p<0.001), short and backwards (OR = 0.3, p<0.01) in the central zone (OR = 0.6, p<0.01) and made in the 16´-30´ (OR = 0.6, p<0.01), 61´-75´ (OR = 0.7, p< 0.05) periods, presented higher probabilities of continuing with possession. Match status losing>2 (OR = 4.1, p< 0.05) showed higher probabilities of success. On the other hand, throw-ins from the defensive zone presented higher probabilities of unsuccess (OR = 8.6, p<0.01) and losing possession (OR = 1.8, p<0.01). Finally, the bottom teams showed the highest probability of losing the ball. In conclusion, tactical indicators such as duration, press, distance, direction and zone were identified as key performance indicators and the situational variables team quality, match status and time influence the outcome of throw-ins. These findings provide valuable insights to coaches regarding the factors that influence the outcome of throw-ins. This allows them to design optimal strategies for both executing and defending these plays based on the game situation and their immediate aims.

## Introduction

At present, high-performance football has been characterized by the meticulous analysis of any factor that may affect the team’s performance [1]. One of the game situations that has been the focus of research interest are set pieces, as they can unbalance the outcome of a match between two teams of equal performance level [2].

In football, it is striking how throw-ins are the set pieces with the highest frequency of execution during matches, averaging 45 throw-ins per game [3], surpassing corner kicks (10.24) [4] and free kicks (30.44) [5]. However, they do not receive an equivalent level of attention from the scientific community or coaches.

It is indisputable that quantitatively these are relevant actions for the game, hence the importance of their study and analysis, with the aim of improving their effectiveness, since, in 64% of cases, ball possession is lost [6]. In a sporting modality in which there is a constant fight for possession, it seems contradictory that an initially advantageous offensive situation for the team, in which the game is stopped and will restart with possession on your hands, has an efficiency of only 36%. Although these are not key game situations in obtaining goals [7], the value of throw-ins should not be underestimated when continuing possession of the ball and prevent the rival team from recovering, as well as to initiate the organization of the offensive phase, and even its use to create finishing situations in the areas of the field closest to the rival goal.

We have found few works focused on the tactical analysis of throw-ins [8] analysed the tactical behavior of the defending teams. Specifically, in this work, a bivariate analysis was carried out to verify if the recoveries of the defending teams were related to the zone of execution of the throw-ins, with pressing or not, and if the pressure varied depending on the match status. Their results indicate that the frequency of recoveries increased when pressure was applied on the executing team, and that the teams that were losing increased the pressure on their opponents. In the work of Stone et al. [9] a bivariate analysis was also carried out to study the relationship between the level of the teams and the outcome of the throw-ins, considering the length, direction and area of the field. Their findings indicated that throwing the ball to the side or backwards increased success rates and that higher-level teams use this strategy more frequently. In the work by García-Paúl et al. [6] a descriptive analysis of the execution tactics of the throw-ins of a semi-professional team was carried out. Their results indicated that 64% of the throw-ins in favor ended in losing ball possession, with forwards throw-ins being more frequent (82%). Lastly, McKinley [10] created expected throw-ins models (xThrow and xRetain) in which the influence variables of outcome, angle, location, distance from own goal and the time since the last action were included. This author concluded that the throw-ins executed backwards are more effective, the less effective throw-ins are those next to the team´s goals, short throw-ins are more successful than long throw-ins and the optimal time to take a throw-in is about five seconds after the ball goes out of bounds to retain possession.

To date, and to the best of our knowledge, no prior work has conducted a multivariate analysis of these game situations. As Casal et al. [11] point out, performance analysis in football must be carried out taking into account the complexity and multifaceted nature of the game. Therefore, we must consider the influence of the context in which tactical actions occur. This context is defined through a set of situational variables that have been shown to impact team performance and behavior, such as match location, match status, team quality, and the area of the field where the analysed behavior takes place. Additionally, these same authors emphasize the need to study the interaction that occurs during the match development among the different analysed variables, using a multivariate analysis.

In this research, we aimed to address some of the identified methodological deficiencies or shortcomings highlighted in previous studies. Specifically, in our study, we analysed the interaction among the various variables under examination. Furthermore, we took into consideration the context in which throw-ins were executed, assessing the influence of five situational variables (match location, team quality, match status, time, and final result) on the effectiveness of throw-ins and the offensive performance of teams in these game situations. Taking this into consideration, in this empirical study, we described how La Liga teams executed corner kicks during the 2021–2022 season, identified tactical indicators related to the outcome of plays that start with a throw-in, calculated their predictive power, and analysed the influence of five situational variables on the effectiveness of these plays.

## Methods

### Design

The specific design corresponding to this systematic observation, according to Anguera et al. [12] was a nomothetic/follow up/multidimensional (N/F/M) design, since different behaviors of various teams were recorded throughout the season. Moreover, the recording used an intrasessional *follow-up* observation (frame-by-frame analysis of different matches) and was captured, post event, using the *ad hoc* observation instrument. This study applied systematic, direct, non-participative observation.

### Sample

A total of 2,658 throw-ins were analysed, corresponding to 80 matches from the 2021/22 season of Spanish La Liga and extracted from the Wyscout database [13]. A season of La Liga consists of 380 matches and the sample size was calculated with 95% of confidence level and 1% of margin of error, following the equation: n = 380 / [(0.10 x 0.10 x (380–1))+ 1] = 79.3 [14]. The match’s selection was carried out with a simple random sampling of all games, excluding matches with red cards (teams, when reduced to a numerical disadvantage, can modify their game model, which may introduce bias in the results) and using the WinEpi program [15].

Of the 3,236 throw-ins executed in the 80 matches, 578 were removed from the record for unobservability or injury clearances. The recording of the information was carried out respecting the behavior spontaneity of the players and in their natural environment. According to the Belmont Report [16] the use of public images for research purpose does not require informed consent or the approval of an ethical committee.

### Observational instrument

[17] guidance was followed for the creation of the observation instrument. First, a hierarchical range of behavior units was established, which was implemented through the adoption of basic criteria for behavior segmentation. The creation of the observation instrument was based on the following pillars: i) a previous theoretical framework; ii) criteria and categories compiled empirically in other observational studies; iii) and, finally, novel criteria that were tested in this work. The methodological steps implemented were the following: first, the problem was identified, and an expert scientific group was formed, comprising of two academic and UEFA PRO coaches (with PhDs in Physical Activity and Sports Sciences), with more than ten years of experience in observational methodology and performance football analysis. After consulting the theoretical framework and empirical evidence, a first post-event exploratory observation was made. Then, and after a discussion by the group of experts, the problem was divided into smaller units. Subsequently, an *ad hoc* observation instrument, denominated “*Thrinfoot*” (Table 1), consisting of field format and category systems [18], was created and tested in order to find weaknesses in the instrument itself. Then, after further discussion by the group of experts, the observation instrument was readjusted. Finally, the post-event viewing was carried out, to finalize the implementation of the observation instrument. This observational tool was made up of several tactical indicators, five situational variables and the explained variable Outcome of the Offensive Sequences.

**Table 1:** Criteria, categories, and codes to observational toll.

Criteria		Category	Code
Situational Variables	Location LO	**Home**: the observed team plays at home	HM
		**Away**: the observed team plays away from home	AW
	Team quality TQ	**Best teams:** The best seven teams, qualified in the UEFA Champions League, Europa League and Conference League	G1
		**Medium teams:** The seven teams classified in the middle zone of the final classification of the regular season	G2
		**Bottom teams:** The three lower teams that descended from a category and the next three worst ranked	G3
	Final Result FR	**Win:** The attacking team has scored more goals than opponent and won the match	FW
		**Draw:** The attacking team has scored equal goals to opponent and draw the match	FD
		**Loss:** The attacking team has scored fewer goals than opponent and lost the match	FL
	Match Status MS	**Win 1 goal:** The observed team has scored one more goal than the opponent at the time of throw-in	W1
		**Win 2 goal:** The observed team has scored two more goal than the opponent at the time of throw-in	W2
		**Win>2 goal:** The observed team has scored three or more goals than the opponent at the time of throw-in	W3
		**Drawing:** The observed team has scored equal goals to the opposition at the time of the throw-in or no goals had been scored	DR
		**Loss 1:** The observed team has scored one less goals than the opponent at the time of the throw-in	L1
		**Loss 2:** The observed team has scored two less goals than the opponent at the time of the throw-in	L2
		**Loss>2:** The observed team has scored three or fewer goals than the opponent at the time of the throw-in	L3
Time T		**0–15 Minutes:** The throw-in was carried out within 0–15 minutes of the match time	0–15
		**16–30 Minutes:** The throw-in was carried out within 16–30 minutes of the match time	16–30
		**31–45+ Minutes:** The throw-in was carried out within 31 minutes–half time	31–45
		**46–60 Minutes:** The throw-in was carried out within 46–60 minutes of the match time	46–60
		**61–75 Minutes:** The throw-in was carried out within 61–75 minutes of the match time	61–75
		**76–90+ Minutes:** The throw-in was carried out within 76 minutes–full time	76–90
Duration D (McKinley) [10]		**Fast:** The throw-in was executed within 5 seconds of the ball goes out of touch	FT
		**Slow:** The throw-in was executed after 5 seconds of the ball goes out of touch	SW
Press PR Adapted from Augste and Prestel [8]		**Pressing:** When the player being thrown at was attacked by a defending player in a clearly man-oriented manner before the throw-in was executed. The defending player immediately tried to exert the highest possible pressure on the opponent through direct duel. At best, the defender tried to force the first contact with the ball after the throw-in in order to be able to continue the game himself	PRS
		**No pressing:** There was no recognizable pressure in the form of approaching the opponent, or the distance of the defending player to the player in possession of the ball was too great to disturb him in the controlled continuation of play, or when a certain distance was initially allowed to the player who has received the throw-in. However, after the first contact with the ball the player was immediately put under pressure. In addition, if the player was covered but not actively tackled.	CONT
Mobility MV		**Yes:** When the player being thrown at moves before executing the throw-in to get rid of this direct rival and thus be able to contact the ball, or teammate moves to create uncertainty in the defending players	YES
		**No:** No attacking player moves to receive the ball	NO
Distance DT		**Short:** The ball was thrown a distance between 0–10 m.	ST
		**Medium:** The ball was thrown a distance between 11–20 m.	ME
		**Long:** The ball was thrown a distance of 21 m. or longer	LG
Direction DR Augste and Prestel [8]		**Backward:** When the ball was thrown into the 90° sector between an imaginary line perpendicular to the sideline and the sideline in the direction of the own goal	BW
		**Forward:** When it was thrown in the direction of the opponent’s goal	FW
Side of attack SD		**Right:** The throw-in was executed on the right side of the observed team	RT
		**Left:** The throw-in was executed on the left side of the observed team	LT
Zone ZN Casal et al. [34]		**Defensive:** The throw-in as performed in the defensive zone of the pitch	DF
		**Middle Defensive:** The throw-in was performed in the middle defensive zone of the pitch	MD
		**Central:** The throw-in was performed in the central zone of the pitch	CE
		**Middle Offensive:** The throw-in was performed in the middle offensive zone of the pitch	MO
		**Offensive:** The throw-in was performed in the offensive zone of the pitch	OF
Outcome OUT		**Goal:** When a player scored a goal which resulted from the throw-in possession	GO
		**Attempt:** When a player attempted a shot at goal which resulted from the throw-in possession	AT
		**Possession:** The team retained the ball in possession for 7 seconds from the point in which the ball was thrown [7]	PS
		**Set play:** A set piece was awarded to the attacking team in the form of a free kick, corner, penalty kick or throw-in	SP
		**Loss of Possession:** The ball possession is lost with in 7 seconds from the point in which the ball was thrown [7]	LP
		**Attempt against:** The opposing team recovers the ball and created a scoring opportunity	AG
		**Goal against:** The opposing team recovers the ball and scored a goal	GA

### Procedure and reliability

Two observers carried out the data recording. Prior to the coding process, and to reduce inter-observer variability, eight training sessions, lasting two hours, were carried out. In these sessions, 500 throw-ins were recorded following the Losada and Manolov [19] criteria and the criteria of consensual agreement [20] was applied among the two observers, so that recording was only done when there was a full agreement. Four weeks after the initial recording, the recording of 260 randomly selected throw-ins was repeated [21]. A percentage higher than the recommended by the literature (10%) was obtained [22]. To ensure inter- and intra-observer consistency of the data, the Cohens’s Kappa coefficient was calculated [23] for each criterion (Table 2). It revealed *almost perfect* agreement [24].

**Table 2:** Inter and intra-observer concordance.

Category	Inter-observer concordance	Intra-observer concordance
	Kappa	Kappa
Location	1.00	1.00
Team quality	1.00	1.00
Final result	1.00	1.00
Match status	1.00	1.00
Time	1.00	1.00
Duration	1.00	1.00
Press	0.95	0.98
Mobility	0.96	0.97
Distance	0.92	0.96
Direction	0.97	1.00
Side of attack	1.00	1.00
Zone	0.93	1.00
Outcome	0.91	1.00

### Data analysis

In this research we carried out three levels of analysis (univariate, bivariate and multivariate). A descriptive analysis by means of frequencies was carried out to describe the characteristics of the sample and the occurrence of each tactical indicators according to the offensive outcome. A bivariate analysis used contingency tables (with chi-square and association measures) to examine the association between each of the explanatory variables and the explained variable (Outcome). The effect size was calculated using Cramer’s *V* test. Values of *V* = 0.06 to 0.17 would refer to a small effect; V = 0.18 to 0.29 to a medium effect, and *V*> 0.30 would refer to a large effect size [25].

Finally, to examine which factors significantly influenced the outcome of the offensive sequences, a multivariate analysis by means of a multinomial logistic regression analysis was carried out, using the step-back model and presenting the results as odds. The odds ratio is used to assess the strength and direction of the association between two categorical variables, indicating the likelihood of an event occurring or not. It has values ranging from 0 to positive infinity. Values of 1 indicate no relationship. Values greater than 1 indicate a positive influence, while values less than 1 indicate a negative influence. In our study, they helped us determine whether there was a relationship between the analyzed variables and the outcome of the play. For example, we examined whether the zone influenced the outcome, using the offensive zone as a reference. The odds ratio for the defensive zone had a value of 1.899 for the "lost possession" outcome. This means that throw-ins executed from the defensive zone had a higher probability of resulting in a "lost possession" compared to those executed in the offensive zone.

For this analysis, firstly, due to the low frequency of Goal (0.9%) and Goal Against (0.3%) and to a better interpretation of the results, we decided to recodify the outcome of the throw-ins by reducing it in the following: *Success* (Goal and Attempt); *Unsuccess* (Goal against and Attempt Against); *Continued Possession* (Possession and Set Piece) and *Lost Possession*. *Unsuccess* was our reference category. We also calculated the effect size based on Cohen’s d coefficient [26], which measures the distance between the observed means, expressed in terms of the combined standard deviation of the two groups. It suggests that values of 0.20, 0.50, and 0.80 indicate small, medium, and large effect sizes, respectively.

R program (v.4.2) using “MRAN” library was used to run all analysis, and the level of significance for each performance indicator was set at 5% (p<0,05) as usual in comparable scientific studies [27].

## Results

### Usual way of execution for throw-ins

A total of 2,658 throw-ins were analysed within the study, with an average of 40.45 per game, of which 0.9% ended in a goal and 6.2% ended with an attempt. The percentage of possession and lost possession were very similar (37.4% and 37.2% respectively), with those losses of possession resulting in an attempt against (1.5%) or a goal against (0.3%) for the opposing team. The highest percentage of throw-ins were executed at the end of the first (18%) and second half of the match (18.7%) and with the match status in favor or winning (47.7%). The best teams executed more throw-ins than the rest of the teams (42%) and the majority of these were executed in the middle offensive zone (32.1%). Table 3 shows the absolute frequencies and percentages for each indicator in relation to the outcome of offensive sequences.

**Table 3:** Absolute frequencies, percentage occurrence of total distribution and association with outcome.

	Outcome								
	Success N (%)		Unsuccess N (%)		Lost possession N (%)	CP N (%)		χ^2^	Cramer´s *V*
Location	Goal	Attempt	Goal against	Attempt against	Loss possess.	Possession	Set play		
Home	14 (60.9)	88 (53.3)	3 (37.5)	15 (37.5)	445 (44.9)	565 (56.8)	216 (49.4)	32.9***	0.14
Away	9 (39.1)	77 (46.7)	5 (62.5)	25 (62.5)	545 (55.1)	430 (43.2)	221 (50.6)		
**Team Quality**									
Best teams	12 (52.2)	70 (42.4)	2 (25.0)	15 (37.5)	339 (34.2)	500 (50.3)	178 (40.7)	75.1***	0.10
Medium teams	7 (30.4)	52 (31.5)	1 (12.5)	13 (32.5)	314 (31.7)	291 (29.2)	151 (34.6)		
Bottom teams	4 (17.4)	43 (26.1)	5 (62.5)	12 (30.0)	337 (34.0)	204 (20.5)	108 (24.7)		
**Final Result**									
Draw	6 (26.1)	52 (31.5)	1 (12.5)	8 (20.0)	254 (25.6)	250 (25.1)	107 (24.5)	11.3	—
Loss	6 (26.1)	60 (36.4)	5 (62.5)	15 (37.5)	376 (38.0)	355 (35.7)	153 (35.0)		
Win	11 (47.8)	53 (32.1)	2 (25.0)	17 (42.5)	360 (36.4)	390 (39.2)	177 (40.5)		
**Match Status**									
Win 1	4 (17.4)	26 (15.8)	2 (25.0)	5 (12.5)	199 (20.1)	125 (12.6)	89 (20.4)		
Win 2	1 (4.4)	5 (3.0)	0 (0.0)	2 (5.5)	51 (5.2)	49 (4.9)	30 (6.9)		
Win>2	0 (0.0)	0 (0.0)	1 (12.5)	0 (0.0)	9 (0.9)	16 (1.7)	2 (0.5)		
Drawing	10 (43.5)	69 (41.8)	3 (37.5)	22 (55.0)	497 (50.2)	476 (47.8)	191 (43.7)	88.4***	0.08
Lost 1	6 (26.1)	47 (28.5)	1 (12.5)	4 (10.0)	172 (17.4)	240 (24.1)	93 (21.3)		
Lost 2	1 (4.4)	13 (7.9)	1 (12.5)	7 (17.5)	44 (4.4)	76 (7.6)	28 (6.4)		
Lost>2	1 (4.4)	5 (3.0)	0 (0.0)	0 (0.0)	18 (1.8)	13 (1.3)	4 (0.9)		
**Time**									
0–15	0 (0.0)	23 (13.9)	1 (12.5)	10 (25.0)	190 (19.2)	171 (17.3)	77 (17.6)	37.0	—
16–30	5 (21.7)	24 (14.5)	1 (12.5)	3 (7.5)	157 (15.9)	185 (18.6)	77 (17.6)		
31-HT	4 (17.4)	30 (18.2)	0 (0.0)	10 (25.0)	191 (19.3)	165 (16.6)	79 (18.1)		
46–60	7 (30.4)	24 (14.5)	2 (25.0)	9 (22.5)	147 (14.8)	160 (16.1)	68 (15.6)		
61–75	3 (13.0)	22 (13.3)	1 (12.5)	2 (5.0)	114 (11.5)	145 (14.6)	54 (12.4)		
76-FT	4 (17.4)	42 (25.5)	3 (37.5)	6 (15.0)	192 (19.3)	168 (16.9)	82 (18.8)		
**Duration**									
Fast	6 (26.1)	51(31.0)	0 (0.0)	7 (17.5)	115 (11.6)	300 (30.2)	70 (16.0)	123.2***	0.18
Slow	17 (73.9)	114 (69.0)	8 (100)	33 (82.5)	875 (88.4)	695 (69.8)	367 (84.0)		
**Press**									
Presssing	16 (69.6)	137 (83.0)	7 (87.5)	34 (85.0)	915 (92.4)	625 (62.8)	383 (87.6)	294.5***	0.26
No press	7 (30.4)	28 (17.0)	1 (12.5)	6 (15.0)	75 (7.6)	370 (37.2)	54 (12.4)		
**Mobility**									
Yes	22 (95.7)	160 (97.0)	8 (100)	38 (95.0)	967 (97.7)	876 (88.0)	430 (98.4)	106.2***	0.13
No	1 (4.3)	5 (3.0)	0 (0.0)	2 (5.0)	23 (2.3)	119 (12.0)	7 (1.6)		
**Distance**									
Short	11 (47.8)	88 (53.3)	2 (25.0)	24 (60.0)	421 (42.5)	508 (51.1)	230 (52.6)	42.6***	0.08
Medium	8 (34.8)	66 (40)	4 (50.0)	14 (35.0)	446 (45.1)	423 (42.5)	169 (38.7)		
Long	4 (17.4)	11 (6.7)	2 (25.0)	2 (5.0)	123 (12.4)	64 (6.4)	38 (8.7)		
**Direction**									
Forward	14 (60.9)	100 (60.6)	6 (75.0)	27 (67.5)	781 (78.9)	361 (36.3)	320 (73.2)	123.2***	0.30
Backward	9 (39.1)	65 (39.4)	2 (25.0)	13 (32.5)	209 (21.1)	634 (63.7)	117 (26.8)		
**Side of attack**									
Right	12 (52.2)	86 (52.1)	5 (62.5)	15 (37.5)	512 (51.7)	530 (53.3)	227 (51.9)	4.4	—
Left	11 (47.8)	79 (47.9)	3 (37.5)	25 (62.5)	478 (48.3)	465 (46.7)	210 (48.1)		
**Zone**									
Defensive	0 (0.0)	4 (2.4)	1 (12.5)	7 (17.5)	85 (8.6)	26 (2.6)	14 (3.2)	162.7***	0.13
Middle defensive	5 (21.7)	15 (9.1)	2 (25.0)	14 (35.0)	259 (26.2)	242 (24.3)	102 (23.3)		
Central	2 (8.7)	28 (17)	2 (25.0)	12 (30.0)	203 (20.5)	289 (29.0)	99 (22.7)		
Middle offensive	13 (56.5)	68 (41.2)	0 (0.0)	6 (15.0)	295 (29.8)	331 (33.3)	139 (31.8)		
Offensive	3 (13.0)	50 (30.3)	3 (37.5)	1 (2.5)	148 (14.9)	107 (10.8)	83 (19.0)		

CP: continued possession

***p<0.001

### Association between offensive outcome and tactical dimensions of throw-ins

Except for the variable side of attack, all the tactical indicators showed a significant association with the outcome of offensive sequences (*p*<0.001), with Direction showing the greatest association (*V* = 0.30), followed by Press (*V* = 0.26) and Duration (*V* = 0.18). Slow throw-ins, with pressing, mobility and forward direction, showed the highest percentages for each outcome. The medium throw-ins showed higher percentages of goal against (50%) and loss of possession (45.1%). On the other hand, short throw-ins obtained higher percentages of possession (51.1%) and set pieces (52.6%). However, the effect size was small (*V* = 0.08). The highest percentage of goals (56.5%) and attempts (41.2%) were obtained through a throw-in executed from the offensive middle zone, showing a small to medium effect size (*V* = 0.13).

### Association between offensive outcome and situational variables

All situational variables showed a significant association with outcome (*p*<0.001), except Final result and Time. The home and best teams were more effective than the rest of the teams and the bottoms teams received the most goals against (62.5%), showing a small effect size respectively (*V* = 0.14, *V* = 0.10). Match status drawing obtained the highest percentages in all results. However, the effect size was small (*V* = 0.08).

### Predictor of outcome of offensive sequences started by a throw-in

Table 4 shows the results of the multinomial logistic regression analysis. The model explained 22% of the changes in throw-in outcome, suggesting that it is a good fit with the data. The accuracy of the model using the ensemble was 0.7813, this is 78.13%. The Cohen’s d coefficient [26] had a small value of 0.224, according to [22], (small, ES = 0.20–0.49; medium, ES = 0.50–0.70 or large, ES>0.80).

**Table 4:** Tactical dimensions and situational variables that affect the outcome between lost possession, success and unsuccess vs continued possession. Results from a multinomial logistic regression.

	OUTCOME								
	Lost possession			Success			Unsuccess		
	β	OR	IC (95%)	β	OR	IC (95%)	β	OR	IC (95%)
**Location**									
Away	0.185	1.203	(0.994–1.455)	-0.033	0.967	(0.689–1.358)	0.450	1.568	(0.813–3.022)
Home ^#^									
**Team_Quality**									
Best teams	-0.711	0.491	**(0.389–0.621)** ***	-0.121	0.886	(0.580–1.354)	-0.655	0.520	(0.245–1.100)
Medium teams	-0.276	0.759	**(0.598–0.962)** **	-0.082	0.921	(0.596–1.422)	-0.322	0.725	(0.339–1.550)
Bottom teams^#^									
**Final Result**									
Draw	0.116	1.124	(0.872–1.448)	0.259	1.295	(0.851–1.972)	-0.401	0.669	(0.310–1.446)
Loss	0.049	1.050	(0.801–1.377)	-0.139	0.871	(0.541–1.401)	-0.374	0.688	(0.316–1.496)
Win ^#^									
**Match Status**									
Win 1	0.221	1.247	(812–1.915)	0.768	2.156	(0.848–5.477)	0.094	1.099	(0.214–5.628)
Win >2	-0.051	0.950	(0.372–2.430)	-18.445	9.759E-9	(0.000–0.000)	1.102	3.009	(0.244–37.031)
Drawing	0.163	1.177	(0.773–1.793)	0.806	2.238	(0.902–5.555	0.214	1.239	(0.257–5.980)
Lost 1	-0.230	0.795	(0.509–1.240)	0.906	2.474	**(0.980–6.244)** *	-0.582	0.559	(0.097–3.225)
Lost 2	-0.396	0.673	(0.386–1.172)	0.772	2.164	(0.754–6.208)	0.914	2.494	(0.458–13.571)
Lost>2	0.137	1.147	(0.505–2.609)	1.424	4.154	**(1.108–15.574)** *	-8.710	7.489E9	(7.489E9-7.489E9)
Win2^#^									
**Time**									
0–15	-0.120	0.887	(0.644–1.220)	-0.740	0.477	**(0.267–0.852)** *	0.150	1.162	(0.414–3.260)
16–30	-0.446	0.640	**(0.469–0.874)** **	-0.524	0.592	**(0.349–1.004)** *	-1.009	0.365	(0.104–1.275)
31-HT	-0.032	0.969	(0.720–1.303)	-0.321	0.726	(0.441–1.194)	0.127	1.135	(0.435–2.958)
46–60	-0.211	0.810	(0.596–1.100)	-0.337	0.714	(0.431–1.182)	0.176	1.192	(0.473–3.007)
61–75	-0.317	0.728	**(0.527–1.007)** *	-0.448	0.639	(0.373–1.092)	-0.798	0.450	(0.118–1.725)
76-FT^#^									
**Duration**									
Fast	-0.281	0.755	**(0.581–0.980)** *	0.304	1.356	(0.926–1.985)	-0.111	0.895	(0.363–2.207)
Slow^#^									
**Press**									
No press	-0.817	0.442	**(0.319–0.612)** ***	-0.173	0.841	(0.523–1.354)	-0.457	0.633	(0.223–1.801)
Presssing ^#^									
**Mobility**									
No	-0.218	0.804	(0.472–1.370)	-0.408	0.665	(0.270–1.638)	-0.249	0.780	(0.154–3.956)
Yes ^#^									
**Distance**									
Long	0.532	1.702	**(1.241–2.336)** ***	0.208	1.231	(0.671–2.257)	-0.133	0.875	(0.292–2.627)
Medium	0.262	1.300	**(1.074–1.573)** **	0.150	1.161	(0.830–1.625)	-0.197	0.821	(0.434–1.555)
Short ^#^									
**Direction**									
Backward	-0.937	0.392	**(0.315–0.487)** ***	-0.632	0.532	**(0.369–0.766)** **	-0.505	0.604	(0.285–1.278)
Forward ^#^									
**Side of attack**									
Left	0.065	1.067	(0.894–1.274)	0.052	1.053	(0.770–1.440)	0.476	1.609	(0.886–2.921)
Right ^#^									
**Zone**									
Defensive	0.641	1.899	**(1.193–3.023)** **	-1.140	0.320	**(0.108–0.946)** *	2.159	8.658	**(2.398–31.267)** **
Middle defensive	-0.263	0.768	(0.569–1.037)	-1.642	0.194	**(0.110–0.341)** ***	0.688	1.990	(0.631–6.279)
Central	-0.509	0.601	**(0.445–0.812)** **	-1.332	0.264	**(0.160–0.435)** ***	0.506	1.658	(0.522–5.271)
Middle offensive	-0.251	0.778	(0.587–1.031)	-0.526	0.591	**(0.397–0.881)** *	-0.520	0.595	(0.163–2.164)
Offensive^#^									

^#^, Reference category; β, Coefficient; OR, Odds Ratio; IC, Confidence interval for odds ratio

* p < 0.05

** p < 0.01

*** p < 0.001.

Throw-ins executed in the first thirty minutes, compared to those executed in the final minutes, decreased the odds of a successful outcome in favor of continued possession by 52.3% (OR = 0.477) and 40.8% (OR = 0.592) respectively. However, the IC values of 16–30 period (0.349–1.004) indicate that we should not take this association into account. Throw-ins executed in the 16–30 and 61–75 periods, compared to those executed in the 76-FT period, decreased the odds of lost possession by 36% (OR = 0.640) and 27.2% (OR = 0.728) respectively in favor of continued possession. However, in the 61–75 period, their IC values (0.527–1.007) make us rule out this probability. Fast throw-ins decreased the odds of losing possession by 24.5% (OR = 0.755) in favor of continuing possession, compared to slow throw-ins. Throw-ins without pressure of the opposing team decreased the odds of lost possession by 55,8% (OR = 0.442) in favor of continued possession. The long and medium throw-ins increased the probabilities of lost possession by 70% (OR = 1.702) and 30% (OR = 1.300) respectively, compared to shot throw-ins. Backwards throw-ins decreased the odds of lost possession by 60.8% (OR = 0.392) in favor of continued possession, compared to forwards throw-ins. On the other hand, the chances of success in favor to continued possession decreased by 46.8% (OR = 0.532), compared to forward throw-ins.

Throw-ins executed from the defensive zone compared to those executed from the offensive zone had 89.9% (OR = 1.899) higher probability of lost possession, 68% (OR = 0.320) less probability of success and 765.8% (OR = 8.658; IC = 2.398–31.267) higher chances of unsuccess, compared to continued possession. The central zone, compared to the offensive zone, decreased by 39.9% (OR = 0.601) the odds of lost possession in favor continued possession and decreased by 73.6% (OR = 0.264) the odds of success in favor to continued possession. The middle defensive and offensive zone decreased the chances of success by 80.6% (OR = 0.194) and 40.9% (OR = 0.591) compared to continued possession.

Regarding situational variables, the best and medium teams, compared to bottom teams, showed 50.9% (OR = 0.491) and 24.1% (OR = 0.759) lower probabilities of lost possession in favor of continued possession. Match status losing, compared to winning 2 increased success chance by 147.4% (OR = 2.474). However, the IC values (0.98–6.24) do not allow us to confirm this statement. Match status losing>2, compared to winning 2, increased success chances by 315.4% (OR = 4.154).

## Discussion

The aim of the study was to describe the usual practices in the execution of throw-ins by La Liga teams during the 2021–2022 season, identify tactical indicators related to the outcome of playing strategies that start with a throw-in, calculate their predictive power, and finally, analyse the influence of situational variables on the effectiveness in these game situations. The results of the univariate analysis show how the average number of throw-ins per game was 40.45, these data being very similar to those provided by Augste and Cordes [3], (44.8); Izquierdo et al. [28], (51.55); Stone et al. [9], (43) and Siegle and Lames [29], (39.69), coinciding with these authors in pointing out that it is the most frequent set piece play during matches. The offensive sequences initiated with a throw-in had a success rate of 7.1% in terms of goals or goal attempts. On the other hand, the possession and loss of possession percentages were very similar, reaching 37.4% and 37.2%, respectively. These results are consistent with those reported by Stone et al. [9] who obtained a shooting rate of 8.8% and a ball possession of 54%. Finally, the probabilities of a loss of possession resulting in an attempt against or a goal against for the opposing team were 1.5% and 0.3%, respectively. These data show the high frequency and ineffectiveness of this set piece play, highlighting the need for greater attention from the coaching staff.

The bivariate analysis allowed us to verify how, except for final result, time and side of attack, all the tactical indicators and the situational variables analysed showed a significant relationship with outcome. Specifically, home teams scored more goals in plays started with a throw-in (60.9%) than away teams (39.1%) and the latter conceded more goals against (62.5%) and attempts against (62.5%). Therefore, home advantage affects the performance of throw-ins, increasing their effectiveness in home teams. However, the effect size was small. Despite not having previous studies that allow us to compare our results, these data agree with previous work indicating that playing at home is associated with greater offensive performance [30, 31]. Consequently, these plays can be used by teams to gain an offensive advantage when playing at home and try to gain a defensive advantage over visiting teams. On the contrary, they have to pay a lot of defensive attention when they play away from home. On the other hand, the mobility of the teammates in possession of the ball also turned out to be related to the outcome, with mobile throw-ins being more effective, although the effect size was also small and this data could possibly be explained by the fact that mobility occurred in more than 90% of the throw-ins. There are no previous results on this indicator in this game situation either, but they do coincide with those of other set pieces, such as corner kicks [4] and indirect free kicks [5, 32]. Therefore, to increase efficiency in this type of play, they must ensure that potential receivers do not remain static and move to try to get clear and receive the ball.

Finally, the multivariate analysis of the multinomial logistic regression identified the tactical indicators and the situational variables that predict the outcome of the offensive sequences started with a throw-in. The execution time of the throw-in has shown a relationship with the result. Specifically, fast throw-ins increased the odds of continuing possession compared to losing possession. These results agree with the work of McKinley [10] which indicates that the optimal time to take a throw-in is about five seconds after the ball goes out of bounds to retain possession. If a throw-in is executed prior to this, the throwers teammates may not yet be prepared to receive the ball, and after five seconds it allows the opposing team to get set to defend. Pressure of opposing teams also showed a relationship with outcome. Throw-ins without pressure of opposing team increased the odds of continuing possession compared to losing possession [8] also reported a 54% ball recovery success rate with high pressing from the defending team. Consequently, coaches should instruct their teams to try to execute throw-ins quickly to avoid the opposing team from organizing defensively and start to add pressure.

The short throw-ins presented higher probabilities of continued possession compared to the medium and long throw-ins, respectively. These data are also corroborated by McKinley [10] and compared with the pass. A short throw-in is more precise and covers less distance than a long or medium one, getting the ball to the teammates faster, decreasing the defensive response time. Therefore, to ensure possession of the ball, the throw-in must be short.

Backwards throw-ins had higher odds of continued possession versus lost possession and had higher odds of continued possession compared to success. The results allow us to indicate that short backwards throw-ins allow us to ensure possession of the ball, but, nevertheless, they decrease our chances of achieving an attempt or goal. However, this type of throw-in will not be valid to try to create a finishing situation because the opposing team will have time to organize defensively [9] also indicate that long and forward throw-ins have a lower success rate.

The area from which the throw-in was executed showed a significant relationship with offensive performance. Specifically, throw-ins executed from the defensive zone compared to those executed from the offensive zone had higher probability of lost possession, goal and goal against and less probability of success, instead to continued possession. The central zone increased the chances of continued possession compared to lost possession and decreased the chances of success compared to continued possession. The middle defensive and offensive zone increased the chances of continued possession compared to success. These data coincide with the information provided by McKinley [10] who indicates that the closer a throw-in is executed to the own goal, the greater the chances of losing the ball. We believe that this could be explained because almost 82% of the throw-ins executed from the defensive zone went forward and, as we have already indicated, with this type of throw-in it is more likely that possession of the ball is lost. Besides, this author also indicates that teams tend to use throw-ins at the defensive zone of opposing team as triggers to press and regain possession because, as [33] point out, the higher up in the field a team wins turnovers, the more likely it is to score goals. Consequently, teams should avoid awarding throw-ins in the defensive zone and pay close attention to defensive transitions on throw-ins executed from this area.

Regarding situational variables, the best and medium teams showed higher probabilities of continued possession compared to bottom teams. [9] also reported that the top ranked teams had higher success rates than the rest, corroborating the correlation between throw-in performance and team performance. Previous studies [34] have shown a relationship between ball possession and team performance, indicating that the best teams are characterized by longer possession times than the rest. Therefore, a throw-in can be executed as a strategy to maintain ball possession, which favors the chances of victory.

The time of the match at which the throw-in is executed has also been shown to be related to its outcome. The last 15 minutes of the match showed a higher probability of goal and goal attempts that in the first half hour. This situation could be explained by the lower defensive pressure exerted by the rival team in this period of the match [9]. We could also compare these results with those obtained in the study of the corner kicks of Casal et al. [4] who had higher odds of goals in the last 30 minutes of the match, indicating that these results could be explained due to the greater physical and mental fatigue experienced by the defenders and/or to the fact that attacking teams tend to take more initiative and risks towards the end of a game, particularly if they are losing. Therefore, teams should take advantage of the throw-ins executed at the end of the match to try to create goals or attempts.

Finally, match status also showed a significant relationship with the performance of throw-ins and offensive sequences. Specifically, losing >2, compared to winning <2, increased the chances of success. In other words, the score against favored the success of the offensive sequences started with a throw-in. In the study [8] it was verified how losing teams increase defensive pressure on throw-ins. As indicated above, pressure from the opposing team is a key factor in throw-in performance, with the chances of ball recovery increasing and the chances of success for the throwing team decreasing as defensive pressure increases.

As for the limitations, we must report that, by including the additional times in the third and sixth periods of the match period variable, a bias may arise, as all periods could not have the same length. However, this way of classifying the variable is the most used by researchers. Other technical-tactical indicators could also be explored, such as the interaction context, the game styles and the role of the player taking the throw-in to increase the predictive capacity of the model.

The aim of the study was to describe the usual practices in the execution of throw-ins by La Liga teams during the 2021–2022 season, identify tactical indicators related to the outcome of plays that start with a throw-in and calculate their predictive power. Additionally, the influence of situational variables on the effectiveness of these plays was analysed. This information can be used by coaches to develop tactical models that increase the likelihood of successfully concluding these game situations, both offensively and defensively. For example, if the aim is to retain ball possession, an effective strategy could involve executing a quick backward and short pass. On the other hand, if the opposing team is about to take a throw-in near their goal, a recommended strategy would be to apply pressure to attempt regaining possession. Although we believe that the results can be extrapolated to other professional leagues, it would be interesting to analyse the other major European competitions, such as the Italian Serie A, the Bundesliga, or the Premier League.

## Conclusions

The results obtained in this study have allowed us to identify the key performance indicators of throw-ins and the situational variables that modify their performance. Specifically, fast and backwards throw-ins, avoiding pressure and taking advantage of the defensive disorganization of opposing team, have a greater chance of continuing possession, although the chances of goal or attempt decrease. Short throw-ins are more effective than long ones. The areas close to the goal itself offer a greater chance of losing possession than those further away. The last fifteen minutes of the game showed higher chances of goals and attempts. The best teams showed a higher percentage of success of the throw-ins. Finally, match status modulates the performance of throw-ins, increasing performance when losing by more than two goals.

## Supporting information

S1 File (PDF)
