# Supplementary material for: Effects of tactical dimension and situational variables in throw-ins on the offensive performance in football
Source: PLoS One. 2023 Nov 15;18(11):e0294317. doi: 10.1371/journal.pone.0294317 (PMC10650988; doi:10.1371/journal.pone.0294317)
Supplement: S1 File — (PDF) [file pone.0294317.s001.pdf]

LOSS	0-15	HM	3	DR	SW	PRS
LOSS	0-15	HM	3	DR	SW	PRS
LOSS	0-15	HM	3	L1	SW	PRS
LOSS	16-30	HM	3	L1	SW	PRS
LOSS	16-30	HM	3	L1	SW	PRS
LOSS	16-30	HM	3	DR	SW	PRS
LOSS	16-30	HM	3	DR	FT	CNT
LOSS	16-30	HM	3	DR	SW	PRS
LOSS	31-45+	HM	3	DR	SW	PRS
LOSS	31-45+	HM	3	DR	SW	PRS
LOSS	46-60	HM	3	DR	SW	PRS
LOSS	46-60	HM	3	DR	SW	CNT
LOSS	46-60	HM	3	DR	SW	PRS
LOSS	46-60	HM	3	DR	FT	CNT
LOSS	61-75	HM	3	DR	SW	PRS
LOSS	61-75	HM	3	DR	SW	PRS
LOSS	61-75	HM	3	L1	SW	PRS
LOSS	76-90+	HM	3	L1	SW	CNT
LOSS	76-90+	HM	3	L1	SW	PRS
LOSS	61-75	HM	3	L1	FT	CNT
DRAW	0-15	AW	3	DR	SW	PRS
DRAW	16-30	AW	3	DR	FT	CNT
DRAW	31-45+	AW	3	DR	SW	PRS
DRAW	46-60	AW	3	DR	SW	PRS
DRAW	61-75	AW	3	DR	SW	PRS
DRAW	76-90+	AW	3	DR	SW	PRS
DRAW	76-90+	AW	3	DR	SW	PRS
LOSS	0-15	AW	3	L1	SW	PRS
LOSS	0-15	AW	3	L1	SW	PRS
LOSS	16-30	AW	3	L1	FT	CNT
LOSS	16-30	AW	3	L1	SW	PRS
LOSS	16-30	AW	3	L1	SW	PRS
LOSS	16-30	AW	3	L1	SW	PRS
LOSS	16-30	AW	3	L1	SW	PRS
LOSS	16-30	AW	3	L1	FT	CNT
LOSS	16-30	AW	3	L1	SW	PRS
LOSS	31-45+	AW	3	L2	SW	CNT
LOSS	31-45+	AW	3	L2	SW	PRS
LOSS	31-45+	AW	3	L2	SW	CNT
LOSS	31-45+	AW	3	L2	SW	PRS
LOSS	31-45+	AW	3	L2	SW	CNT
LOSS	31-45+	AW	3	L2	FT	PRS
LOSS	31-45+	AW	3	L>2	SW	PRS
LOSS	46-60	AW	3	L2	SW	PRS
LOSS	46-60	AW	3	L>2	SW	PRS
LOSS	76-90+	AW	3	L2	SW	PRS
LOSS	76-90+	AW	3	L>2	SW	PRS
LOSS	76-90+	AW	3	L>2	SW	CNT
LOSS	76-90+	AW	3	L>2	SW	PRS
LOSS	76-90+	AW	3	L>2	SW	PRS

WIN	0-15	AW	3	DR	SW	PRS
WIN	0-15	AW	3	DR	SW	PRS
WIN	0-15	AW	3	DR	SW	PRS
WIN	0-15	AW	3	DR	SW	PRS
WIN	0-15	AW	3	DR	SW	PRS
WIN	16-30	AW	3	DR	SW	PRS
WIN	16-30	AW	3	W1	SW	PRS
WIN	16-30	AW	3	W1	SW	PRS
WIN	16-30	AW	3	W1	SW	PRS
WIN	31-45+	AW	3	W1	SW	PRS
WIN	31-45+	AW	3	W1	SW	PRS
WIN	31-45+	AW	3	W1	SW	PRS
WIN	31-45+	AW	3	W1	SW	PRS
WIN	31-45+	AW	3	W1	SW	PRS
WIN	31-45+	AW	3	W1	SW	PRS
WIN	31-45+	AW	3	W1	SW	PRS
WIN	46-60	AW	3	DR	SW	PRS
WIN	46-60	AW	3	DR	SW	PRS
WIN	61-75	AW	3	L1	SW	PRS
WIN	61-75	AW	3	L1	SW	PRS
WIN	61-75	AW	3	L1	SW	PRS
WIN	76-90+	AW	3	L1	SW	PRS
WIN	76-90+	AW	3	L1	FT	PRS
WIN	76-90+	AW	3	L1	FT	PRS
WIN	76-90+	AW	3	L1	SW	PRS
LOSS	0-15	AW	3	DR	SW	PRS
LOSS	0-15	AW	3	DR	SW	PRS
LOSS	16-30	AW	3	DR	SW	PRS
LOSS	16-30	AW	3	DR	SW	PRS
LOSS	16-30	AW	3	DR	SW	PRS
LOSS	16-30	AW	3	DR	SW	PRS
LOSS	16-30	AW	3	DR	SW	PRS
LOSS	31-45+	AW	3	DR	SW	PRS
LOSS	31-45+	AW	3	DR	SW	PRS
LOSS	31-45+	AW	3	DR	SW	PRS
LOSS	46-60	AW	3	DR	SW	PRS
LOSS	46-60	AW	3	DR	SW	PRS
LOSS	46-60	AW	3	DR	SW	PRS
LOSS	61-75	AW	3	L1	FT	PRS
LOSS	61-75	AW	3	L1	SW	PRS
LOSS	61-75	AW	3	L1	FT	CNT
LOSS	61-75	AW	3	L1	SW	PRS
LOSS	61-75	AW	3	L1	FT	PRS
LOSS	61-75	AW	3	L1	FT	PRS
LOSS	76-90+	AW	3	L1	FT	PRS
LOSS	76-90+	AW	3	L2	SW	PRS
DRAW	0-15	AW	3	DR	SW	PRS
DRAW	0-15	AW	3	W1	SW	PRS
DRAW	0-15	AW	3	W1	SW	PRS
DRAW	16-30	AW	3	W1	SW	PRS

DRAW	16-30	AW	3	W1	SW	PRS
DRAW	16-30	AW	3	W1	SW	PRS
DRAW	16-30	AW	3	W1	SW	PRS
DRAW	16-30	AW	3	W1	SW	PRS
DRAW	16-30	AW	3	W1	SW	PRS
DRAW	16-30	AW	3	W1	SW	PRS
DRAW	31-45+	AW	3	W1	SW	PRS
DRAW	31-45+	AW	3	W1	SW	PRS
DRAW	31-45+	AW	3	DR	SW	PRS
DRAW	46-60	AW	3	W1	SW	PRS
DRAW	46-60	AW	3	W1	SW	PRS
DRAW	76-90+	AW	3	W1	SW	PRS
LOSS	0-15	HM	3	DR	SW	PRS
LOSS	0-15	HM	3	DR	SW	PRS
LOSS	0-15	HM	3	DR	SW	PRS
LOSS	16-30	HM	3	DR	SW	PRS
LOSS	31-45+	HM	3	DR	SW	PRS
LOSS	31-45+	HM	3	DR	SW	PRS
LOSS	31-45+	HM	3	DR	SW	PRS
LOSS	31-45+	HM	3	DR	SW	PRS
LOSS	31-45+	HM	3	DR	SW	PRS
LOSS	46-60	HM	3	DR	SW	PRS
LOSS	46-60	HM	3	DR	SW	PRS
LOSS	46-60	HM	3	DR	SW	PRS
LOSS	61-75	HM	3	DR	FT	PRS
LOSS	76-90+	HM	3	L1	SW	PRS
LOSS	76-90+	HM	3	L1	SW	PRS
LOSS	76-90+	HM	3	L1	SW	PRS
LOSS	76-90+	HM	3	L1	FT	PRS
LOSS	76-90+	HM	3	L1	SW	PRS
LOSS	76-90+	HM	3	L1	SW	PRS
LOSS	76-90+	HM	3	L1	SW	PRS
LOSS	76-90+	HM	3	L1	SW	PRS
LOSS	76-90+	HM	3	L1	FT	PRS
WIN	0-15	HM	1	DR	SW	PRS
WIN	0-15	HM	1	DR	SW	PRS
WIN	0-15	HM	1	DR	SW	PRS
WIN	16-30	HM	1	DR	FT	PRS
WIN	16-30	HM	1	DR	SW	PRS
WIN	31-45+	HM	1	W1	SW	PRS
WIN	31-45+	HM	1	W1	SW	PRS
WIN	46-60	HM	1	W2	FT	CNT
WIN	46-60	HM	1	W2	SW	PRS
WIN	46-60	HM	1	W2	SW	PRS
WIN	46-60	HM	1	W2	SW	PRS
WIN	46-60	HM	1	W2	SW	PRS
WIN	61-75	HM	1	W2	SW	PRS
WIN	76-90+	HM	1	W2	SW	CNT
DRAW	0-15	HM	1	DR	SW	PRS
DRAW	16-30	HM	1	DR	FT	CNT

DRAW	16-30	HM	1	DR	SW	PRS
DRAW	16-30	HM	1	DR	SW	PRS
DRAW	16-30	HM	1	DR	SW	PRS
DRAW	16-30	HM	1	DR	FT	CNT
DRAW	31-45+	HM	1	W1	SW	PRS
DRAW	46-60	HM	1	W1	SW	PRS
DRAW	61-75	HM	1	W2	SW	PRS
DRAW	61-75	HM	1	W2	SW	PRS
DRAW	76-90+	HM	1	W2	SW	PRS
DRAW	76-90+	HM	1	W2	SW	PRS
WIN	0-15	HM	1	DR	SW	PRS
WIN	0-15	HM	1	DR	FT	CNT
WIN	0-15	HM	1	DR	SW	PRS
WIN	0-15	HM	1	DR	SW	PRS
WIN	16-30	HM	1	DR	SW	PRS
WIN	16-30	HM	1	DR	FT	PRS
WIN	31-45+	HM	1	DR	SW	CNT
WIN	31-45+	HM	1	DR	SW	PRS
WIN	46-60	HM	1	W1	SW	PRS
WIN	46-60	HM	1	W1	FT	PRS
WIN	46-60	HM	1	W1	FT	CNT
WIN	46-60	HM	1	W1	FT	CNT
WIN	46-60	HM	1	W1	SW	PRS
WIN	61-75	HM	1	W1	SW	CNT
WIN	76-90+	HM	1	W1	SW	PRS
WIN	76-90+	HM	1	W1	SW	PRS
WIN	76-90+	HM	1	W1	SW	PRS
WIN	0-15	HM	1	DR	SW	PRS
WIN	0-15	HM	1	DR	SW	PRS
WIN	0-15	HM	1	DR	SW	PRS
WIN	0-15	HM	1	DR	SW	PRS
WIN	0-15	HM	1	DR	SW	PRS
WIN	0-15	HM	1	DR	SW	CNT
WIN	0-15	HM	1	DR	SW	PRS
WIN	0-15	HM	1	DR	SW	PRS
WIN	16-30	HM	1	DR	SW	PRS
WIN	16-30	HM	1	DR	SW	PRS
WIN	16-30	HM	1	DR	SW	PRS
WIN	31-45+	HM	1	DR	FT	CNT
WIN	31-45+	HM	1	DR	SW	PRS
WIN	61-75	HM	1	W1	SW	PRS
WIN	61-75	HM	1	W1	SW	PRS
WIN	61-75	HM	1	DR	SW	PRS
WIN	76-90+	HM	1	DR	SW	PRS
WIN	76-90+	HM	1	DR	SW	CNT
WIN	76-90+	HM	1	DR	FT	PRS
WIN	76-90+	HM	1	DR	SW	PRS
DRAW	0-15	HM	1	DR	FT	PRS
DRAW	0-15	HM	1	DR	SW	PRS
DRAW	0-15	HM	1	DR	SW	PRS

DRAW	16-30	HM	1	DR	SW	PRS
DRAW	16-30	HM	1	DR	SW	PRS
DRAW	16-30	HM	1	DR	FT	CNT
DRAW	16-30	HM	1	DR	SW	PRS
DRAW	16-30	HM	1	DR	SW	CNT
DRAW	16-30	HM	1	DR	SW	CNT
DRAW	16-30	HM	1	DR	SW	PRS
DRAW	46-60	HM	1	DR	FT	CNT
DRAW	46-60	HM	1	DR	SW	CNT
DRAW	46-60	HM	1	DR	FT	CNT
DRAW	46-60	HM	1	DR	FT	CNT
DRAW	46-60	HM	1	DR	SW	PRS
DRAW	46-60	HM	1	DR	FT	PRS
DRAW	46-60	HM	1	DR	SW	CNT
DRAW	46-60	HM	1	DR	SW	PRS
DRAW	61-75	HM	1	DR	SW	PRS
DRAW	76-90+	HM	1	DR	SW	PRS
DRAW	76-90+	HM	1	DR	FT	CNT
DRAW	76-90+	HM	1	DR	FT	CNT
DRAW	76-90+	HM	1	DR	FT	CNT
DRAW	76-90+	HM	1	DR	SW	CNT
WIN	0-15	HM	1	DR	SW	PRS
WIN	0-15	HM	1	DR	FT	PRS
WIN	0-15	HM	1	DR	SW	PRS
WIN	0-15	HM	1	DR	SW	PRS
WIN	0-15	HM	1	DR	SW	PRS
WIN	16-30	HM	1	DR	SW	PRS
WIN	31-45+	HM	1	DR	FT	CNT
WIN	31-45+	HM	1	DR	SW	PRS
WIN	31-45+	HM	1	DR	FT	CNT
WIN	31-45+	HM	1	DR	FT	CNT
WIN	46-60	HM	1	DR	FT	PRS
WIN	46-60	HM	1	DR	FT	CNT
WIN	61-75	HM	1	DR	SW	PRS
WIN	61-75	HM	1	DR	SW	PRS
WIN	61-75	HM	1	DR	SW	PRS
WIN	76-90+	HM	1	DR	FT	PRS
WIN	76-90+	HM	1	DR	FT	PRS
WIN	76-90+	HM	1	W1	FT	PRS
WIN	76-90+	HM	1	W1	SW	PRS
DRAW	0-15	HM	1	DR	SW	PRS
DRAW	0-15	HM	1	DR	FT	CNT
DRAW	0-15	HM	1	DR	FT	CNT
DRAW	0-15	HM	1	L1	FT	CNT
DRAW	0-15	HM	1	L1	SW	CNT
DRAW	16-30	HM	1	L1	FT	CNT
DRAW	16-30	HM	1	L1	FT	PRS
DRAW	16-30	HM	1	L1	FT	CNT
DRAW	16-30	HM	1	L1	FT	CNT
DRAW	31-45+	HM	1	L1	SW	PRS

DRAW	46-60	HM	1	L2	FT	CNT
DRAW	46-60	HM	1	L2	SW	PRS
DRAW	46-60	HM	1	L2	FT	CNT
DRAW	61-75	HM	1	L1	SW	PRS
DRAW	61-75	HM	1	L1	SW	PRS
DRAW	61-75	HM	1	L1	SW	CNT
DRAW	61-75	HM	1	L1	SW	CNT
DRAW	61-75	HM	1	L1	SW	PRS
DRAW	61-75	HM	1	L1	SW	CNT
DRAW	76-90+	HM	1	DR	SW	PRS
DRAW	76-90+	HM	1	DR	SW	CNT
DRAW	76-90+	HM	1	DR	FT	CNT
WIN	0-15	HM	1	DR	SW	PRS
WIN	0-15	HM	1	DR	FT	CNT
WIN	16-30	HM	1	DR	SW	CNT
WIN	16-30	HM	1	DR	SW	PRS
WIN	16-30	HM	1	DR	SW	PRS
WIN	31-45+	HM	1	L1	SW	PRS
WIN	31-45+	HM	1	L1	FT	CNT
WIN	31-45+	HM	1	L1	SW	PRS
WIN	31-45+	HM	1	L2	FT	CNT
WIN	31-45+	HM	1	L2	SW	PRS
WIN	46-60	HM	1	L2	FT	CNT
WIN	46-60	HM	1	L2	SW	CNT
WIN	46-60	HM	1	L2	FT	CNT
WIN	46-60	HM	1	L2	FT	CNT
WIN	61-75	HM	1	L2	FT	PRS
WIN	61-75	HM	1	L1	SW	PRS
WIN	61-75	HM	1	L1	FT	CNT
WIN	61-75	HM	1	L1	SW	PRS
WIN	76-90+	HM	1	L1	SW	PRS
WIN	76-90+	HM	1	L1	FT	PRS
WIN	76-90+	HM	1	L1	FT	CNT
WIN	76-90+	HM	1	W1	SW	PRS
WIN	0-15	AW	1	DR	SW	PRS
WIN	0-15	AW	1	DR	SW	CNT
WIN	0-15	AW	1	DR	SW	CNT
WIN	16-30	AW	1	DR	SW	PRS
WIN	31-45+	AW	1	DR	SW	PRS
WIN	31-45+	AW	1	DR	SW	CNT
WIN	31-45+	AW	1	DR	SW	PRS
WIN	46-60	AW	1	DR	SW	PRS
WIN	46-60	AW	1	DR	FT	CNT
WIN	61-75	AW	1	W1	SW	PRS
WIN	76-90+	AW	1	W>2	FT	CNT
WIN	0-15	AW	1	W1	SW	PRS
WIN	16-30	AW	1	W1	SW	PRS
WIN	16-30	AW	1	W1	SW	PRS
WIN	16-30	AW	1	W1	SW	CNT
WIN	31-45+	AW	1	W1	SW	PRS

WIN	31-45+	HM	1	W1	SW	CNT
WIN	31-45+	AW	1	W1	SW	CNT
WIN	31-45+	AW	1	W1	SW	PRS
WIN	31-45+	AW	1	W1	SW	CNT
WIN	31-45+	AW	1	W1	SW	PRS
WIN	46-60	AW	1	DR	SW	PRS
WIN	46-60	AW	1	DR	SW	PRS
WIN	46-60	AW	1	DR	SW	PRS
WIN	61-75	AW	1	W1	SW	PRS
WIN	76-90+	AW	1	W1	SW	PRS
WIN	0-15	AW	1	DR	SW	PRS
WIN	0-15	AW	1	DR	FT	PRS
WIN	0-15	AW	1	W1	FT	PRS
WIN	0-15	AW	1	W1	SW	PRS
WIN	16-30	AW	1	W1	FT	PRS
WIN	16-30	AW	1	W1	SW	PRS
WIN	31-45+	AW	1	W1	SW	PRS
WIN	31-45+	AW	1	W1	FT	PRS
WIN	31-45+	AW	1	W1	SW	PRS
WIN	31-45+	AW	1	W1	SW	PRS
WIN	31-45+	AW	1	W1	SW	PRS
WIN	31-45+	AW	1	W1	FT	PRS
WIN	31-45+	AW	1	W1	SW	PRS
WIN	46-60	AW	1	W1	SW	PRS
WIN	46-60	AW	1	W1	SW	PRS
WIN	46-60	AW	1	W1	SW	PRS
WIN	61-75	AW	1	W2	SW	PRS
WIN	61-75	AW	1	W2	SW	PRS
WIN	61-75	AW	1	W2	SW	PRS
WIN	76-90+	AW	1	W2	SW	PRS
WIN	76-90+	AW	1	W2	SW	PRS
LOSS	0-15	AW	1	DR	SW	PRS
LOSS	0-15	AW	1	DR	FT	PRS
LOSS	16-30	AW	1	L1	SW	PRS
LOSS	16-30	AW	1	L1	SW	PRS
LOSS	31-45+	AW	1	L1	SW	PRS
LOSS	31-45+	AW	1	L1	FT	PRS
LOSS	31-45+	AW	1	L1	SW	CNT
LOSS	46-60	AW	1	W1	SW	CNT
LOSS	46-60	AW	1	L2	SW	CNT
LOSS	61-75	AW	1	L2	SW	CNT
LOSS	76-90+	AW	1	L2	SW	CNT
LOSS	76-90+	AW	1	L2	FT	PRS
LOSS	0-15	AW	1	W1	SW	PRS
LOSS	16-30	AW	1	W1	SW	PRS
LOSS	16-30	AW	1	DR	SW	PRS
LOSS	31-45+	AW	1	DR	FT	PRS
LOSS	31-45+	AW	1	DR	SW	PRS
LOSS	31-45+	AW	1	DR	SW	CNT
LOSS	31-45+	AW	1	DR	SW	PRS

LOSS	46-60	AW	1	DR	SW	CNT
LOSS	46-60	AW	1	DR	SW	CNT
LOSS	61-75	AW	1	L1	FT	PRS
LOSS	61-75	AW	1	L1	SW	PRS
LOSS	61-75	AW	1	L1	FT	CNT
LOSS	61-75	AW	1	L1	FT	PRS
LOSS	61-75	AW	1	L1	SW	CNT
LOSS	76-90+	AW	1	L1	FT	CNT
DRAW	0-15	HM	1	DR	SW	PRS
DRAW	31-45+	HM	1	W1	SW	PRS
DRAW	31-45+	HM	1	W1	SW	PRS
DRAW	31-45+	HM	1	W1	SW	PRS
DRAW	31-45+	HM	1	W1	SW	PRS
DRAW	46-60	HM	1	W1	SW	PRS
DRAW	46-60	HM	1	W1	SW	PRS
DRAW	46-60	HM	1	W1	SW	PRS
DRAW	46-60	HM	1	W1	SW	PRS
DRAW	46-60	HM	1	W1	SW	PRS
DRAW	61-75	HM	1	W1	SW	PRS
DRAW	61-75	HM	1	W1	SW	PRS
DRAW	61-75	HM	1	W1	SW	PRS
DRAW	76-90+	HM	1	W1	SW	PRS
DRAW	76-90+	HM	1	W1	SW	PRS
DRAW	76-90+	HM	1	DR	FT	PRS
WIN	0-15	HM	2	DR	SW	PRS
WIN	0-15	HM	2	DR	SW	PRS
WIN	0-15	HM	2	DR	SW	PRS
WIN	0-15	HM	2	DR	SW	PRS
WIN	16-30	HM	2	DR	SW	PRS
WIN	16-30	HM	2	DR	SW	PRS
WIN	16-30	HM	2	DR	SW	PRS
WIN	31-45+	HM	2	DR	SW	PRS
WIN	31-45+	HM	2	DR	SW	PRS
WIN	46-60	HM	2	DR	SW	PRS
WIN	46-60	HM	2	DR	SW	PRS
WIN	46-60	HM	2	L1	FT	CNT
WIN	61-75	HM	2	L1	SW	PRS
WIN	61-75	HM	2	L1	SW	CNT
WIN	61-75	HM	2	L1	FT	PRS
WIN	61-75	HM	2	L1	SW	PRS
WIN	76-90+	HM	2	DR	SW	PRS
WIN	76-90+	HM	2	DR	SW	PRS
WIN	76-90+	HM	2	W1	SW	PRS
WIN	0-15	HM	2	DR	FT	CNT
WIN	0-15	HM	2	DR	SW	PRS
WIN	0-15	HM	2	DR	SW	CNT
WIN	0-15	HM	2	DR	FT	CNT
WIN	16-30	HM	2	DR	FT	CNT
WIN	16-30	HM	2	DR	FT	PRS
WIN	46-60	HM	2	DR	SW	CNT

WIN	46-60	HM	2	DR	SW	PRS
WIN	46-60	HM	2	DR	SW	PRS
WIN	61-75	HM	2	W1	SW	PRS
WIN	61-75	HM	2	W1	SW	PRS
WIN	61-75	HM	2	W1	SW	CNT
WIN	76-90+	HM	2	W2	SW	PRS
WIN	76-90+	HM	2	W2	SW	PRS
WIN	76-90+	HM	2	W2	SW	PRS
WIN	0-15	HM	2	DR	SW	PRS
WIN	0-15	HM	2	DR	SW	PRS
WIN	0-15	HM	2	DR	SW	PRS
WIN	16-30	HM	2	DR	SW	PRS
WIN	16-30	HM	2	DR	SW	PRS
WIN	31-45+	HM	2	W1	SW	PRS
WIN	31-45+	HM	2	W1	SW	PRS
WIN	31-45+	HM	2	W1	SW	PRS
WIN	31-45+	HM	2	W1	SW	PRS
WIN	31-45+	HM	2	W1	SW	PRS
WIN	31-45+	HM	2	W1	FT	CNT
WIN	61-75	HM	2	W1	SW	PRS
WIN	61-75	HM	2	W1	SW	PRS
WIN	76-90+	HM	2	W2	SW	PRS
LOSS	0-15	AW	2	DR	SW	PRS
LOSS	0-15	AW	2	DR	SW	PRS
LOSS	16-30	AW	2	DR	SW	PRS
LOSS	16-30	AW	2	DR	SW	PRS
LOSS	31-45+	AW	2	DR	SW	PRS
LOSS	31-45+	AW	2	DR	FT	PRS
LOSS	46-60	AW	2	L1	SW	PRS
LOSS	46-60	AW	2	L1	FT	PRS
LOSS	46-60	AW	2	L1	SW	PRS
LOSS	61-75	AW	2	L1	FT	PRS
LOSS	61-75	AW	2	L1	SW	PRS
LOSS	76-90+	AW	2	L1	SW	PRS
LOSS	76-90+	AW	2	L1	SW	PRS
LOSS	76-90+	AW	2	L1	SW	PRS
LOSS	76-90+	AW	2	L1	SW	PRS
DRAW	0-15	AW	2	DR	SW	PRS
DRAW	16-30	AW	2	DR	SW	PRS
DRAW	16-30	AW	2	DR	SW	PRS
DRAW	16-30	AW	2	DR	SW	PRS
DRAW	31-45+	AW	2	DR	SW	PRS
DRAW	31-45+	AW	2	DR	SW	PRS
DRAW	31-45+	AW	2	DR	SW	PRS
DRAW	31-45+	AW	2	DR	SW	PRS
DRAW	31-45+	AW	2	DR	SW	PRS
DRAW	31-45+	AW	2	DR	SW	PRS
DRAW	31-45+	AW	2	DR	SW	PRS
DRAW	31-45+	AW	2	DR	SW	PRS
DRAW	31-45+	AW	2	DR	SW	PRS

DRAW	46-60	AW	2	DR	SW	PRS
DRAW	46-60	AW	2	DR	SW	PRS
DRAW	61-75	AW	2	DR	SW	PRS
DRAW	76-90+	AW	2	DR	SW	PRS
DRAW	76-90+	AW	2	DR	SW	PRS
DRAW	76-90+	AW	2	DR	SW	PRS
DRAW	76-90+	AW	2	DR	SW	PRS
WIN	0-15	AW	2	DR	SW	PRS
WIN	0-15	AW	2	DR	SW	PRS
WIN	0-15	AW	2	DR	SW	PRS
WIN	0-15	AW	2	DR	SW	PRS
WIN	16-30	AW	2	DR	SW	PRS
WIN	16-30	AW	2	DR	SW	PRS
WIN	16-30	AW	2	DR	SW	PRS
WIN	16-30	AW	2	DR	SW	PRS
WIN	31-45+	AW	2	W1	SW	PRS
WIN	31-45+	AW	2	W1	FT	PRS
WIN	31-45+	AW	2	W1	SW	PRS
WIN	31-45+	AW	2	W1	SW	PRS
WIN	31-45+	AW	2	W1	SW	PRS
WIN	46-60	AW	2	W1	SW	PRS
WIN	61-75	AW	2	W1	SW	PRS
WIN	61-75	AW	2	W1	SW	PRS
WIN	76-90+	AW	2	W1	SW	PRS
WIN	76-90+	AW	2	W1	SW	PRS
WIN	76-90+	AW	2	W1	SW	PRS
LOSS	0-15	AW	1	DR	SW	PRS
LOSS	0-15	AW	1	DR	SW	PRS
LOSS	0-15	AW	1	DR	SW	CNT
LOSS	16-30	AW	1	DR	SW	PRS
LOSS	46-60	AW	1	L2	SW	CNT
LOSS	46-60	AW	1	L2	FT	CNT
LOSS	46-60	AW	1	L2	FT	CNT
LOSS	46-60	AW	1	L2	FT	CNT
LOSS	46-60	AW	1	L2	SW	CNT
LOSS	61-75	AW	1	L2	SW	CNT
LOSS	61-75	AW	1	L2	SW	PRS
LOSS	76-90+	AW	1	L2	SW	PRS
LOSS	76-90+	AW	1	L2	SW	PRS
LOSS	76-90+	AW	1	L2	FT	CNT
LOSS	76-90+	AW	1	L2	FT	CNT
WIN	0-15	HM	1	DR	FT	PRS
WIN	0-15	HM	1	DR	SW	PRS
WIN	16-30	HM	1	DR	SW	PRS
WIN	16-30	HM	1	DR	SW	CNT
WIN	31-45+	HM	1	DR	SW	PRS
WIN	31-45+	HM	1	DR	SW	PRS
WIN	46-60	HM	1	DR	FT	CNT
WIN	46-60	HM	1	DR	SW	PRS
WIN	46-60	HM	1	DR	SW	PRS

WIN	76-90+	HM	1	W1	SW	PRS
DRAW	0-15	HM	1	DR	FT	PRS
DRAW	0-15	HM	1	DR	SW	PRS
DRAW	0-15	HM	1	DR	SW	PRS
DRAW	0-15	HM	1	DR	FT	PRS
DRAW	0-15	HM	1	DR	SW	CNT
DRAW	0-15	HM	1	DR	FT	CNT
DRAW	0-15	HM	1	DR	SW	CNT
DRAW	16-30	HM	1	DR	SW	PRS
DRAW	31-45+	HM	1	DR	SW	PRS
DRAW	31-45+	HM	1	DR	FT	PRS
DRAW	31-45+	HM	1	DR	FT	CNT
DRAW	31-45+	HM	1	DR	FT	CNT
DRAW	46-60	HM	1	DR	SW	CNT
DRAW	46-60	HM	1	DR	SW	CNT
DRAW	46-60	HM	1	DR	SW	PRS
DRAW	61-75	HM	1	DR	SW	PRS
DRAW	61-75	HM	1	DR	FT	CNT
DRAW	61-75	HM	1	DR	FT	CNT
DRAW	61-75	HM	1	DR	SW	CNT
DRAW	76-90+	HM	1	DR	SW	PRS
DRAW	76-90+	HM	1	DR	FT	CNT
WIN	0-15	HM	1	DR	SW	PRS
WIN	31-45+	HM	1	W2	SW	PRS
WIN	46-60	HM	1	W2	FT	CNT
WIN	61-75	HM	1	DR	SW	PRS
WIN	61-75	HM	1	DR	SW	CNT
WIN	76-90+	HM	1	W1	SW	PRS
WIN	76-90+	HM	1	W1	SW	PRS
WIN	0-15	HM	1	DR	SW	PRS
WIN	0-15	HM	1	DR	FT	CNT
WIN	0-15	HM	1	DR	SW	PRS
WIN	0-15	HM	1	DR	FT	CNT
WIN	0-15	HM	1	DR	SW	PRS
WIN	16-30	HM	1	DR	FT	CNT
WIN	16-30	HM	1	DR	SW	CNT
WIN	31-45+	HM	1	W1	SW	CNT
WIN	31-45+	HM	1	W1	FT	CNT
WIN	46-60	HM	1	W1	SW	PRS
WIN	46-60	HM	1	W1	SW	CNT
WIN	46-60	HM	1	W>2	FT	CNT
WIN	61-75	HM	1	W2	SW	PRS
WIN	76-90+	HM	1	W2	SW	PRS
WIN	76-90+	HM	1	W1	SW	PRS
WIN	76-90+	HM	1	W1	SW	PRS
WIN	76-90+	HM	1	W1	SW	PRS
WIN	76-90+	HM	1	W1	SW	PRS
WIN	76-90+	HM	1	W1	SW	PRS
WIN	0-15	HM	1	L1	SW	CNT
WIN	16-30	HM	1	DR	SW	CNT

WIN	16-30	HM	1	DR	SW	PRS
WIN	31-45+	HM	1	DR	FT	CNT
WIN	31-45+	HM	1	W1	SW	PRS
WIN	46-60	HM	1	W1	SW	PRS
WIN	46-60	HM	1	W1	SW	PRS
WIN	46-60	HM	1	W1	SW	PRS
WIN	46-60	HM	1	W1	SW	PRS
WIN	61-75	HM	1	W1	SW	PRS
WIN	61-75	HM	1	W1	SW	PRS
WIN	61-75	HM	1	W1	SW	PRS
WIN	76-90+	HM	1	W1	SW	PRS
WIN	76-90+	HM	1	W2	SW	PRS
WIN	0-15	AW	1	DR	SW	PRS
WIN	0-15	AW	1	DR	SW	PRS
WIN	0-15	AW	1	DR	SW	PRS
WIN	0-15	AW	1	DR	SW	PRS
WIN	0-15	AW	1	DR	SW	PRS
WIN	0-15	AW	1	DR	SW	PRS
WIN	16-30	AW	1	DR	SW	PRS
WIN	16-30	AW	1	DR	SW	PRS
WIN	16-30	AW	1	DR	SW	PRS
WIN	31-45+	AW	1	W1	SW	PRS
WIN	31-45+	AW	1	W2	SW	PRS
WIN	31-45+	AW	1	W>2	SW	PRS
WIN	46-60	AW	1	W2	SW	PRS
WIN	61-75	AW	1	W2	SW	PRS
WIN	76-90+	AW	1	W>2	SW	CNT
WIN	76-90+	AW	1	W>2	SW	PRS
WIN	76-90+	AW	1	W>2	SW	PRS
WIN	76-90+	AW	1	W>2	SW	PRS
WIN	0-15	AW	1	DR	SW	PRS
WIN	0-15	AW	1	DR	FT	CNT
WIN	0-15	AW	1	DR	SW	PRS
WIN	16-30	AW	1	DR	SW	PRS
WIN	31-45+	AW	1	DR	SW	PRS
WIN	46-60	AW	1	DR	FT	CNT
WIN	46-60	AW	1	W1	SW	PRS
WIN	46-60	AW	1	W1	SW	CNT
WIN	61-75	AW	1	W1	SW	PRS
WIN	61-75	AW	1	W1	SW	PRS
WIN	61-75	AW	1	W1	SW	PRS
WIN	76-90+	AW	1	W1	SW	PRS
WIN	76-90+	AW	1	DR	SW	PRS
WIN	76-90+	AW	1	W1	SW	PRS
WIN	0-15	AW	1	DR	SW	PRS
WIN	16-30	AW	1	DR	SW	PRS
WIN	16-30	AW	1	DR	SW	PRS
WIN	31-45+	AW	1	W1	SW	CNT
WIN	31-45+	AW	1	W1	SW	PRS
WIN	46-60	AW	1	W>2	SW	PRS

LOSS	0-15	AW	1	DR	SW	PRS
LOSS	16-30	AW	1	DR	FT	PRS
LOSS	16-30	AW	1	DR	SW	PRS
LOSS	16-30	AW	1	DR	SW	PRS
LOSS	16-30	AW	1	DR	FT	PRS
LOSS	16-30	AW	1	DR	FT	PRS
LOSS	31-45+	AW	1	L1	SW	PRS
LOSS	31-45+	AW	1	L1	SW	PRS
LOSS	31-45+	AW	1	L1	SW	PRS
LOSS	31-45+	AW	1	L1	SW	PRS
LOSS	46-60	AW	1	L1	SW	PRS
LOSS	46-60	AW	1	L1	SW	PRS
LOSS	61-75	AW	1	L1	SW	PRS
LOSS	61-75	AW	1	L1	FT	PRS
LOSS	61-75	AW	1	L1	SW	PRS
LOSS	61-75	AW	1	L1	FT	PRS
LOSS	76-90+	AW	1	L1	SW	PRS
WIN	0-15	AW	1	DR	SW	CNT
WIN	0-15	AW	1	DR	SW	CNT
WIN	0-15	AW	1	DR	SW	PRS
WIN	0-15	AW	1	DR	SW	PRS
WIN	0-15	AW	1	DR	FT	CNT
WIN	16-30	AW	1	DR	SW	CNT
WIN	31-45+	AW	1	DR	SW	PRS
WIN	31-45+	AW	1	DR	SW	PRS
WIN	31-45+	AW	1	DR	SW	PRS
WIN	46-60	AW	1	DR	SW	CNT
WIN	46-60	AW	1	L1	SW	PRS
WIN	76-90+	AW	1	L1	SW	CNT
WIN	76-90+	AW	1	W1	SW	PRS
DRAW	0-15	AW	1	DR	SW	PRS
DRAW	0-15	AW	1	DR	FT	PRS
DRAW	0-15	AW	1	DR	SW	PRS
DRAW	0-15	AW	1	DR	SW	PRS
DRAW	0-15	AW	1	DR	SW	CNT
DRAW	16-30	AW	1	DR	SW	CNT
DRAW	16-30	AW	1	DR	SW	PRS
DRAW	16-30	AW	1	DR	SW	PRS
DRAW	16-30	AW	1	DR	SW	CNT
DRAW	16-30	AW	1	DR	SW	PRS
DRAW	31-45+	AW	1	DR	FT	CNT
DRAW	31-45+	AW	1	DR	FT	PRS
DRAW	31-45+	AW	1	DR	SW	PRS
DRAW	31-45+	AW	1	DR	FT	CNT
DRAW	46-60	AW	1	DR	SW	PRS
DRAW	61-75	AW	1	DR	SW	PRS
DRAW	76-90+	AW	1	DR	SW	CNT
DRAW	76-90+	AW	1	DR	SW	CNT
DRAW	76-90+	AW	1	DR	SW	PRS
DRAW	76-90+	AW	1	DR	SW	PRS

LOSS	16-30	AW	1	DR	FT	CNT
LOSS	31-45+	AW	1	DR	SW	PRS
LOSS	31-45+	AW	1	DR	SW	CNT
LOSS	31-45+	AW	1	DR	SW	CNT
LOSS	31-45+	AW	1	DR	SW	PRS
LOSS	31-45+	AW	1	DR	FT	PRS
LOSS	31-45+	AW	1	DR	FT	PRS
LOSS	46-60	AW	1	DR	SW	PRS
LOSS	46-60	AW	1	DR	SW	PRS
LOSS	46-60	AW	1	W1	SW	PRS
LOSS	61-75	AW	1	W1	SW	CNT
LOSS	76-90+	AW	1	DR	SW	CNT
LOSS	76-90+	AW	1	DR	SW	CNT
LOSS	76-90+	AW	1	DR	SW	PRS
LOSS	76-90+	AW	1	DR	SW	PRS
LOSS	76-90+	AW	1	L1	SW	PRS
LOSS	76-90+	AW	1	L1	SW	PRS
WIN	0-15	HM	1	DR	SW	PRS
WIN	0-15	HM	1	DR	SW	PRS
WIN	0-15	HM	1	DR	SW	PRS
WIN	0-15	HM	1	DR	SW	PRS
WIN	16-30	HM	1	DR	FT	PRS
WIN	16-30	HM	1	DR	SW	PRS
WIN	16-30	HM	1	DR	SW	PRS
WIN	46-60	HM	1	W2	SW	CNT
WIN	46-60	HM	1	W2	SW	CNT
WIN	46-60	HM	1	W2	SW	PRS
WIN	61-75	HM	1	W1	SW	PRS
WIN	61-75	HM	1	W1	SW	PRS
WIN	61-75	HM	1	W1	SW	PRS
WIN	61-75	HM	1	W1	SW	CNT
WIN	0-15	HM	1	DR	SW	PRS
WIN	0-15	HM	1	DR	SW	PRS
WIN	0-15	HM	1	DR	SW	PRS
WIN	0-15	HM	1	DR	FT	CNT
WIN	16-30	HM	1	W1	FT	CNT
WIN	16-30	HM	1	W1	SW	PRS
WIN	16-30	HM	1	W1	SW	PRS
WIN	16-30	HM	1	W1	SW	CNT
WIN	31-45+	HM	1	W2	SW	PRS
WIN	31-45+	HM	1	W2	SW	PRS
WIN	31-45+	HM	1	W2	SW	PRS
WIN	46-60	HM	1	W>2	SW	PRS
WIN	46-60	HM	1	W>2	SW	CNT
WIN	61-75	HM	1	W>2	SW	PRS
WIN	61-75	HM	1	W>2	SW	CNT
WIN	76-90+	HM	1	W>2	SW	PRS
LOSS	0-15	HM	1	L1	SW	CNT
LOSS	0-15	HM	1	L1	SW	CNT
LOSS	16-30	HM	1	L1	FT	CNT

LOSS	31-45+	HM	1	L1	SW	PRS
LOSS	31-45+	HM	1	L1	SW	CNT
LOSS	31-45+	HM	1	L1	FT	CNT
LOSS	46-60	HM	1	DR	FT	PRS
LOSS	46-60	HM	1	DR	SW	CNT
LOSS	61-75	HM	1	L1	SW	CNT
LOSS	76-90+	HM	1	L1	SW	CNT
LOSS	76-90+	HM	1	L1	FT	CNT
LOSS	76-90+	HM	1	L2	SW	CNT
DRAW	0-15	HM	1	DR	SW	CNT
DRAW	0-15	HM	1	DR	SW	CNT
DRAW	0-15	HM	1	DR	SW	CNT
DRAW	0-15	AW	1	DR	SW	PRS
DRAW	0-15	HM	1	DR	SW	PRS
DRAW	16-30	HM	1	DR	SW	PRS
DRAW	16-30	HM	1	DR	SW	PRS
DRAW	31-45+	HM	1	DR	SW	CNT
DRAW	31-45+	HM	1	DR	FT	CNT
DRAW	46-60	HM	1	DR	SW	CNT
DRAW	46-60	HM	1	DR	SW	PRS
DRAW	46-60	HM	1	DR	FT	CNT
DRAW	76-90+	HM	1	DR	SW	CNT
DRAW	76-90+	HM	1	DR	FT	CNT
DRAW	76-90+	HM	1	DR	FT	CNT
DRAW	76-90+	AW	1	DR	SW	PRS
DRAW	76-90+	AW	1	DR	SW	PRS
WIN	0-15	HM	1	DR	SW	PRS
WIN	0-15	HM	1	DR	SW	PRS
WIN	16-30	HM	1	L1	FT	CNT
WIN	31-45+	HM	1	L1	SW	PRS
WIN	31-45+	HM	1	L1	FT	PRS
WIN	31-45+	HM	1	L1	SW	CNT
WIN	31-45+	HM	1	L1	FT	PRS
WIN	31-45+	HM	1	L1	SW	PRS
WIN	31-45+	HM	1	L1	FT	CNT
WIN	46-60	HM	1	L1	SW	PRS
WIN	46-60	HM	1	L1	FT	CNT
WIN	61-75	HM	1	DR	SW	PRS
WIN	61-75	HM	1	DR	SW	PRS
WIN	61-75	HM	1	W1	SW	PRS
WIN	61-75	HM	1	W1	SW	PRS
WIN	76-90+	HM	1	W1	SW	PRS
WIN	76-90+	HM	1	W1	SW	PRS
WIN	76-90+	HM	1	W2	SW	PRS
WIN	76-90+	HM	1	W2	SW	CNT
WIN	76-90+	HM	1	W2	SW	PRS
WIN	0-15	HM	1	DR	SW	CNT
WIN	0-15	HM	1	DR	SW	PRS
WIN	0-15	HM	1	DR	SW	PRS
WIN	16-30	HM	1	DR	SW	PRS

WIN	16-30	HM	1	DR	SW	CNT
WIN	16-30	HM	1	W1	SW	PRS
WIN	31-45+	HM	1	W1	SW	PRS
WIN	31-45+	HM	1	W1	FT	PRS
WIN	31-45+	HM	1	W1	SW	CNT
WIN	31-45+	HM	1	W1	FT	PRS
WIN	31-45+	HM	1	W1	FT	CNT
WIN	31-45+	HM	1	W1	FT	PRS
WIN	46-60	HM	1	W1	SW	PRS
WIN	46-60	HM	1	W1	SW	PRS
WIN	61-75	HM	1	W1	SW	CNT
WIN	76-90+	HM	1	DR	SW	PRS
WIN	76-90+	HM	1	W1	SW	PRS
WIN	76-90+	HM	1	W1	SW	PRS
LOSS	0-15	HM	1	DR	SW	PRS
LOSS	0-15	HM	1	DR	SW	PRS
LOSS	0-15	HM	1	DR	SW	CNT
LOSS	16-30	HM	1	DR	SW	PRS
LOSS	16-30	HM	1	DR	SW	PRS
LOSS	16-30	HM	1	DR	SW	PRS
LOSS	16-30	HM	1	DR	SW	PRS
LOSS	16-30	HM	1	DR	SW	PRS
LOSS	31-45+	HM	1	DR	SW	PRS
LOSS	31-45+	HM	1	L1	SW	PRS
LOSS	46-60	AW	1	L1	FT	PRS
LOSS	61-75	HM	1	L1	FT	CNT
LOSS	61-75	HM	1	L1	FT	CNT
LOSS	76-90+	HM	1	L1	SW	CNT
LOSS	76-90+	HM	1	L1	SW	PRS
LOSS	76-90+	HM	1	L2	FT	PRS
LOSS	76-90+	HM	1	L2	SW	CNT
WIN	0-15	HM	1	DR	SW	PRS
WIN	0-15	HM	1	DR	SW	PRS
WIN	0-15	HM	1	DR	SW	PRS
WIN	0-15	HM	1	DR	SW	PRS
WIN	16-30	HM	1	W1	SW	PRS
WIN	16-30	HM	1	W1	SW	PRS
WIN	16-30	HM	1	W1	SW	CNT
WIN	16-30	HM	1	W1	SW	PRS
WIN	16-30	HM	1	W1	SW	PRS
WIN	16-30	HM	1	W1	SW	PRS
WIN	31-45+	HM	1	W2	SW	PRS
WIN	31-45+	HM	1	W2	SW	PRS
WIN	31-45+	HM	1	W2	SW	PRS
WIN	31-45+	HM	1	W1	SW	PRS
WIN	31-45+	HM	1	W1	SW	PRS
WIN	31-45+	HM	1	W1	SW	PRS
WIN	46-60	HM	1	W1	SW	PRS
WIN	46-60	HM	1	W1	SW	PRS
WIN	46-60	HM	1	W1	SW	PRS

WIN	61-75	HM	1	W2	SW	PRS
WIN	76-90+	HM	1	W>2	SW	PRS
WIN	0-15	AW	1	DR	FT	CNT
WIN	0-15	AW	1	DR	SW	PRS
WIN	0-15	AW	1	DR	SW	PRS
WIN	0-15	AW	1	DR	SW	PRS
WIN	16-30	AW	1	DR	SW	PRS
WIN	16-30	AW	1	DR	SW	PRS
WIN	16-30	AW	1	W1	SW	PRS
WIN	16-30	AW	1	W1	SW	PRS
WIN	16-30	AW	1	W1	SW	PRS
WIN	31-45+	AW	1	W1	SW	PRS
WIN	31-45+	AW	1	DR	SW	PRS
WIN	46-60	AW	1	DR	SW	PRS
WIN	46-60	AW	1	DR	SW	PRS
WIN	46-60	AW	1	DR	SW	PRS
WIN	46-60	AW	1	DR	SW	CNT
WIN	61-75	AW	1	DR	SW	PRS
WIN	61-75	AW	1	DR	FT	PRS
WIN	76-90+	AW	1	W1	SW	PRS
LOSS	0-15	AW	1	DR	SW	PRS
LOSS	0-15	AW	1	DR	SW	PRS
LOSS	16-30	AW	1	DR	SW	PRS
LOSS	16-30	AW	1	L1	SW	PRS
LOSS	16-30	AW	1	L1	SW	PRS
LOSS	16-30	AW	1	L1	SW	PRS
LOSS	31-45+	AW	1	L1	SW	PRS
LOSS	31-45+	AW	1	L1	FT	PRS
LOSS	31-45+	AW	1	L1	SW	PRS
LOSS	31-45+	AW	1	L2	SW	PRS
LOSS	46-60	AW	1	L2	SW	PRS
LOSS	46-60	AW	1	L2	SW	PRS
LOSS	46-60	AW	1	L2	FT	PRS
LOSS	46-60	AW	1	L2	FT	PRS
LOSS	46-60	AW	1	L2	SW	PRS
LOSS	61-75	AW	1	L2	FT	PRS
LOSS	61-75	AW	1	L2	SW	PRS
LOSS	61-75	AW	1	L2	SW	PRS
LOSS	76-90+	AW	1	L2	SW	PRS
LOSS	76-90+	AW	1	L2	FT	PRS
LOSS	0-15	HM	3	DR	SW	CNT
LOSS	0-15	HM	3	DR	SW	CNT
LOSS	0-15	HM	3	DR	SW	PRS
LOSS	16-30	HM	3	DR	SW	PRS
LOSS	16-30	HM	3	DR	SW	PRS
LOSS	16-30	HM	3	DR	SW	PRS
LOSS	31-45+	HM	3	DR	SW	PRS
LOSS	31-45+	HM	3	DR	SW	PRS
LOSS	46-60	HM	3	DR	SW	PRS
LOSS	46-60	HM	3	DR	FT	CNT

LOSS	0-15	HM	3	DR	SW	PRS
LOSS	0-15	HM	3	DR	SW	PRS
LOSS	16-30	HM	3	DR	SW	PRS
LOSS	16-30	HM	3	DR	SW	PRS
LOSS	16-30	HM	3	DR	SW	PRS
LOSS	16-30	HM	3	DR	SW	PRS
LOSS	16-30	HM	3	DR	SW	PRS
LOSS	31-45+	HM	3	DR	SW	PRS
LOSS	46-60	HM	3	DR	SW	PRS
LOSS	46-60	HM	3	DR	FT	PRS
LOSS	46-60	HM	3	DR	SW	PRS
LOSS	61-75	HM	3	W1	SW	PRS
LOSS	61-75	HM	3	W1	SW	PRS
LOSS	61-75	HM	3	W1	FT	CNT
LOSS	61-75	HM	3	W1	SW	PRS
LOSS	76-90+	HM	3	W1	SW	PRS
LOSS	76-90+	HM	3	L1	SW	PRS
LOSS	76-90+	HM	3	L1	SW	PRS
LOSS	76-90+	HM	3	L1	SW	PRS
DRAW	0-15	HM	3	L1	SW	CNT
DRAW	0-15	HM	3	L1	SW	PRS
DRAW	0-15	HM	3	L1	FT	CNT
DRAW	0-15	HM	3	L1	FT	CNT
DRAW	16-30	HM	3	L1	SW	CNT
DRAW	16-30	HM	3	L1	SW	PRS
DRAW	16-30	HM	3	L1	FT	CNT
DRAW	16-30	HM	3	L1	FT	CNT
DRAW	31-45+	HM	3	L1	FT	CNT
DRAW	31-45+	HM	3	L1	SW	CNT
DRAW	31-45+	HM	3	L1	SW	CNT
DRAW	31-45+	HM	3	L1	SW	PRS
DRAW	46-60	HM	3	DR	SW	PRS
DRAW	46-60	HM	3	DR	SW	PRS
DRAW	61-75	HM	3	DR	FT	CNT
DRAW	61-75	HM	3	DR	SW	PRS
DRAW	76-90+	HM	3	DR	FT	PRS
DRAW	76-90+	HM	3	DR	SW	CNT
DRAW	76-90+	HM	3	DR	SW	CNT
DRAW	76-90+	HM	3	DR	SW	PRS
DRAW	0-15	HM	3	DR	SW	PRS
DRAW	0-15	HM	3	DR	SW	CNT
DRAW	16-30	HM	3	DR	FT	PRS
DRAW	31-45+	HM	3	L1	SW	PRS
DRAW	46-60	HM	3	L1	SW	PRS
DRAW	46-60	HM	3	L1	SW	CNT
DRAW	61-75	HM	3	L1	SW	PRS
DRAW	61-75	HM	3	L1	SW	CNT
DRAW	61-75	HM	3	L1	SW	PRS
DRAW	61-75	HM	3	L1	FT	PRS
DRAW	61-75	HM	3	L1	FT	PRS

DRAW	61-75	HM	3	L1	SW	CNT
DRAW	76-90+	HM	3	L1	SW	CNT
DRAW	76-90+	HM	3	L1	FT	CNT
DRAW	76-90+	HM	3	L1	SW	PRS
DRAW	76-90+	HM	3	L1	FT	PRS
LOSS	0-15	HM	3	DR	SW	PRS
LOSS	0-15	HM	3	DR	SW	PRS
LOSS	0-15	HM	3	DR	SW	PRS
LOSS	31-45+	HM	3	DR	SW	PRS
LOSS	31-45+	HM	3	DR	SW	PRS
LOSS	31-45+	HM	3	DR	SW	PRS
LOSS	31-45+	HM	3	DR	FT	PRS
LOSS	31-45+	HM	3	DR	SW	PRS
LOSS	31-45+	HM	3	DR	SW	PRS
LOSS	61-75	HM	3	L1	FT	PRS
LOSS	61-75	HM	3	L1	FT	CNT
WIN	0-15	AW	3	DR	SW	PRS
WIN	0-15	AW	3	DR	SW	PRS
WIN	0-15	AW	3	DR	SW	PRS
WIN	0-15	AW	3	DR	FT	PRS
WIN	0-15	AW	3	DR	SW	PRS
WIN	0-15	AW	3	DR	SW	PRS
WIN	0-15	AW	3	DR	SW	PRS
WIN	16-30	AW	3	DR	SW	PRS
WIN	16-30	AW	3	DR	SW	PRS
WIN	16-30	AW	3	DR	SW	PRS
WIN	16-30	AW	3	DR	SW	PRS
WIN	16-30	AW	3	DR	FT	CNT
WIN	31-45+	AW	3	DR	SW	PRS
WIN	31-45+	AW	3	DR	SW	PRS
WIN	31-45+	AW	3	W2	SW	PRS
WIN	46-60	AW	3	W2	SW	PRS
WIN	46-60	AW	3	W2	SW	PRS
WIN	61-75	AW	3	W1	SW	PRS
WIN	61-75	AW	3	W1	SW	PRS
WIN	61-75	AW	3	W1	SW	PRS
WIN	76-90+	AW	3	W1	SW	PRS
WIN	76-90+	AW	3	W1	SW	PRS
DRAW	0-15	AW	3	DR	SW	PRS
DRAW	0-15	AW	3	DR	SW	PRS
DRAW	0-15	AW	3	DR	SW	PRS
DRAW	16-30	AW	3	W1	SW	PRS
DRAW	31-45+	AW	3	DR	SW	CNT
DRAW	31-45+	AW	3	DR	SW	PRS
DRAW	31-45+	AW	3	DR	SW	PRS
DRAW	31-45+	AW	3	DR	SW	CNT
DRAW	31-45+	HM	3	DR	SW	PRS
DRAW	46-60	AW	3	DR	SW	PRS
DRAW	46-60	AW	3	DR	SW	PRS
DRAW	76-90+	AW	3	DR	SW	PRS

DRAW	76-90+	AW	3 DR	SW	PRS
DRAW	76-90+	AW	3 DR	SW	PRS
DRAW	76-90+	AW	3 DR	SW	PRS
LOSS	0-15	AW	3 DR	SW	PRS
LOSS	0-15	AW	3 DR	FT	PRS
LOSS	0-15	AW	3 L1	SW	PRS
LOSS	16-30	AW	3 L1	SW	PRS
LOSS	16-30	AW	3 L1	SW	PRS
LOSS	31-45+	AW	3 L1	SW	PRS
LOSS	31-45+	AW	3 L1	FT	PRS
LOSS	31-45+	AW	3 L1	FT	PRS
LOSS	61-75	AW	3 L1	SW	PRS
LOSS	61-75	AW	3 L1	FT	CNT
LOSS	61-75	AW	3 L1	FT	CNT
LOSS	61-75	AW	3 L1	FT	PRS
LOSS	61-75	AW	3 L1	FT	PRS
DRAW	0-15	AW	3 DR	SW	PRS
DRAW	0-15	AW	3 DR	SW	PRS
DRAW	0-15	AW	3 DR	SW	PRS
DRAW	31-45+	AW	3 DR	SW	PRS
DRAW	46-60	AW	3 DR	SW	PRS
DRAW	61-75	AW	3 DR	SW	PRS
LOSS	0-15	AW	3 DR	SW	PRS
LOSS	16-30	AW	3 DR	SW	PRS
LOSS	16-30	AW	3 L1	FT	PRS
LOSS	31-45+	AW	3 L1	SW	PRS
LOSS	31-45+	AW	3 L1	SW	PRS
LOSS	46-60	AW	3 L1	SW	PRS
LOSS	46-60	AW	3 L2	SW	PRS
LOSS	61-75	AW	3 L2	SW	PRS
LOSS	61-75	AW	3 L2	SW	PRS
LOSS	76-90+	AW	3 L2	SW	PRS
LOSS	76-90+	AW	3 L2	SW	PRS
LOSS	76-90+	AW	3 L2	SW	PRS
LOSS	76-90+	AW	3 L2	SW	PRS
LOSS	76-90+	AW	3 L2	SW	PRS
LOSS	76-90+	AW	3 L2	SW	PRS
LOSS	76-90+	AW	3 L2	SW	PRS
WIN	0-15	AW	3 DR	SW	PRS
WIN	0-15	AW	3 DR	SW	PRS
WIN	16-30	AW	3 DR	SW	PRS
WIN	16-30	AW	3 DR	SW	PRS
WIN	16-30	AW	3 DR	SW	PRS
WIN	31-45+	AW	3 DR	SW	PRS
WIN	46-60	AW	3 DR	SW	PRS
WIN	46-60	AW	3 DR	SW	PRS
WIN	46-60	AW	3 DR	FT	PRS
WIN	46-60	AW	3 DR	FT	PRS
WIN	61-75	AW	3 DR	SW	PRS
WIN	61-75	AW	3 DR	SW	PRS

WIN	61-75	AW	3	DR	SW	PRS
WIN	61-75	AW	3	DR	FT	PRS
WIN	61-75	AW	3	DR	FT	PRS
WIN	76-90+	AW	3	W1	SW	PRS
WIN	76-90+	AW	3	W1	SW	PRS
WIN	76-90+	AW	3	W1	SW	PRS
WIN	0-15	AW	2	DR	SW	PRS
WIN	0-15	AW	2	DR	SW	PRS
WIN	0-15	AW	2	DR	SW	PRS
WIN	0-15	AW	2	DR	SW	PRS
WIN	0-15	AW	2	DR	SW	CNT
WIN	0-15	AW	2	DR	SW	CNT
WIN	31-45+	AW	2	DR	FT	CNT
WIN	31-45+	AW	2	DR	FT	CNT
WIN	46-60	AW	2	DR	SW	PRS
WIN	61-75	AW	2	DR	SW	PRS
WIN	61-75	AW	2	DR	SW	PRS
WIN	61-75	AW	2	DR	SW	PRS
WIN	61-75	AW	2	W1	SW	CNT
WIN	61-75	AW	2	W1	SW	PRS
WIN	61-75	AW	2	W1	SW	PRS
WIN	61-75	AW	2	W1	SW	PRS
WIN	76-90+	AW	2	W1	SW	PRS
WIN	76-90+	AW	2	W1	SW	PRS
WIN	76-90+	AW	2	W1	SW	PRS
WIN	76-90+	AW	2	W1	SW	PRS
LOSS	0-15	AW	2	DR	SW	PRS
LOSS	0-15	AW	2	DR	SW	CNT
LOSS	0-15	AW	2	DR	SW	PRS
LOSS	0-15	AW	2	DR	SW	CNT
LOSS	16-30	AW	2	DR	FT	CNT
LOSS	16-30	AW	2	DR	SW	PRS
LOSS	31-45+	AW	2	DR	SW	CNT
LOSS	31-45+	AW	2	L1	SW	CNT
LOSS	31-45+	AW	2	L1	SW	CNT
LOSS	31-45+	AW	2	L1	FT	PRS
LOSS	31-45+	AW	2	L1	SW	PRS
LOSS	31-45+	AW	2	L1	FT	PRS
LOSS	31-45+	AW	2	L1	FT	CNT
LOSS	31-45+	AW	2	L1	FT	PRS
LOSS	46-60	AW	2	L1	SW	PRS
LOSS	46-60	AW	2	L1	FT	CNT
LOSS	61-75	AW	2	L2	SW	PRS
LOSS	61-75	AW	2	L2	FT	PRS
LOSS	61-75	AW	2	L2	SW	PRS
LOSS	76-90+	AW	2	L2	FT	CNT
LOSS	76-90+	AW	2	L2	SW	PRS
LOSS	76-90+	AW	2	L2	SW	CNT
LOSS	76-90+	AW	2	L2	FT	PRS
LOSS	76-90+	AW	2	L2	SW	CNT

LOSS	76-90+	AW	2	L2	FT	CNT
LOSS	76-90+	AW	2	L2	SW	PRS
DRAW	0-15	HM	2	DR	SW	PRS
DRAW	0-15	HM	2	DR	SW	PRS
DRAW	0-15	HM	2	DR	SW	CNT
DRAW	0-15	HM	2	DR	SW	CNT
DRAW	0-15	HM	2	DR	SW	PRS
DRAW	0-15	HM	2	DR	SW	PRS
DRAW	0-15	HM	2	DR	SW	PRS
DRAW	16-30	HM	2	DR	FT	CNT
DRAW	31-45+	HM	2	DR	SW	PRS
DRAW	31-45+	HM	2	DR	SW	CNT
DRAW	31-45+	HM	2	DR	SW	PRS
DRAW	31-45+	HM	2	DR	SW	CNT
DRAW	31-45+	HM	2	DR	SW	PRS
DRAW	46-60	HM	2	DR	FT	CNT
DRAW	61-75	HM	2	DR	FT	CNT
DRAW	61-75	HM	2	DR	SW	PRS
DRAW	61-75	HM	2	DR	FT	CNT
DRAW	76-90+	HM	2	DR	SW	PRS
DRAW	0-15	HM	2	DR	SW	CNT
DRAW	0-15	HM	2	DR	FT	PRS
DRAW	0-15	HM	2	DR	SW	CNT
DRAW	0-15	HM	2	DR	FT	CNT
DRAW	16-30	HM	2	DR	SW	CNT
DRAW	16-30	HM	2	DR	FT	PRS
DRAW	16-30	HM	2	DR	SW	PRS
DRAW	16-30	HM	2	DR	FT	CNT
DRAW	16-30	HM	2	DR	FT	CNT
DRAW	16-30	HM	2	DR	SW	PRS
DRAW	31-45+	HM	2	DR	SW	CNT
DRAW	31-45+	HM	2	L1	FT	CNT
DRAW	31-45+	HM	2	L2	FT	CNT
DRAW	46-60	HM	2	L2	FT	CNT
DRAW	46-60	HM	2	L2	SW	PRS
DRAW	61-75	HM	2	L2	FT	CNT
DRAW	61-75	HM	2	L2	FT	CNT
DRAW	61-75	HM	2	L1	SW	CNT
DRAW	76-90+	HM	2	L1	FT	CNT
DRAW	76-90+	HM	2	L1	SW	CNT
DRAW	76-90+	HM	2	L1	FT	PRS
DRAW	76-90+	HM	2	L1	SW	PRS
DRAW	76-90+	HM	2	L1	FT	CNT
DRAW	76-90+	HM	2	L1	FT	CNT
DRAW	76-90+	HM	2	L1	FT	PRS
DRAW	76-90+	HM	2	L1	SW	PRS
DRAW	76-90+	HM	2	L1	FT	CNT
DRAW	76-90+	HM	2	L1	FT	CNT
DRAW	76-90+	HM	2	L1	FT	CNT
DRAW	76-90+	HM	2	L1	SW	PRS

DRAW	76-90+	HM	2	L1	FT	CNT
DRAW	76-90+	HM	2	L1	FT	CNT
WIN	0-15	HM	2	DR	SW	PRS
WIN	0-15	HM	2	DR	FT	CNT
WIN	0-15	HM	2	DR	SW	PRS
WIN	0-15	HM	2	DR	FT	CNT
WIN	0-15	HM	2	DR	SW	CNT
WIN	16-30	HM	2	DR	SW	CNT
WIN	16-30	HM	2	DR	FT	PRS
WIN	31-45+	HM	2	DR	FT	CNT
WIN	31-45+	HM	2	DR	SW	CNT
WIN	31-45+	HM	2	DR	SW	CNT
WIN	31-45+	HM	2	DR	FT	CNT
WIN	31-45+	HM	2	DR	SW	PRS
WIN	31-45+	HM	2	DR	FT	PRS
WIN	46-60	HM	2	DR	SW	PRS
WIN	46-60	HM	2	DR	SW	PRS
WIN	46-60	HM	2	DR	SW	CNT
WIN	61-75	HM	2	DR	FT	CNT
WIN	61-75	HM	2	DR	SW	PRS
WIN	61-75	HM	2	DR	SW	PRS
WIN	61-75	HM	2	DR	SW	PRS
WIN	76-90+	HM	2	DR	FT	PRS
WIN	76-90+	HM	2	DR	FT	PRS
WIN	76-90+	HM	2	DR	FT	CNT
WIN	76-90+	HM	2	DR	SW	PRS
WIN	76-90+	HM	2	DR	FT	PRS
DRAW	0-15	HM	2	DR	FT	CNT
DRAW	0-15	HM	2	DR	SW	PRS
DRAW	16-30	HM	2	DR	SW	PRS
DRAW	16-30	HM	2	DR	FT	CNT
DRAW	31-45+	HM	2	DR	SW	PRS
DRAW	31-45+	HM	2	DR	SW	PRS
DRAW	46-60	HM	2	DR	FT	PRS
DRAW	46-60	HM	2	DR	SW	PRS
DRAW	46-60	HM	2	DR	FT	PRS
DRAW	46-60	HM	2	DR	SW	CNT
DRAW	46-60	HM	2	DR	SW	CNT
DRAW	61-75	HM	2	DR	FT	CNT
DRAW	76-90+	HM	2	W1	SW	PRS
DRAW	76-90+	HM	2	W1	SW	PRS
DRAW	76-90+	HM	2	W1	SW	PRS
DRAW	76-90+	HM	2	DR	SW	PRS
LOSS	0-15	HM	2	DR	SW	PRS
LOSS	0-15	HM	2	DR	SW	PRS
LOSS	0-15	HM	2	DR	SW	PRS
LOSS	0-15	HM	2	DR	FT	CNT
LOSS	0-15	HM	2	DR	SW	PRS
LOSS	0-15	HM	2	DR	SW	PRS
LOSS	0-15	HM	2	DR	SW	PRS

LOSS	16-30	HM	2 DR	SW	PRS
LOSS	16-30	HM	2 DR	FT	PRS
LOSS	16-30	HM	2 DR	SW	PRS
LOSS	16-30	HM	2 DR	SW	PRS
LOSS	16-30	HM	2 DR	SW	PRS
LOSS	31-45+	HM	2 DR	SW	PRS
LOSS	31-45+	HM	2 DR	SW	PRS
LOSS	46-60	HM	2 DR	SW	PRS
LOSS	46-60	HM	2 DR	SW	PRS
LOSS	61-75	HM	2 L1	SW	PRS
LOSS	61-75	HM	2 L1	SW	PRS
LOSS	61-75	HM	2 L1	FT	PRS
LOSS	61-75	HM	2 L1	SW	CNT
LOSS	61-75	HM	2 L1	SW	PRS
LOSS	76-90+	HM	2 L1	SW	PRS
LOSS	76-90+	HM	2 L1	FT	CNT
LOSS	76-90+	HM	2 L1	FT	CNT
LOSS	76-90+	HM	2 L1	FT	PRS
DRAW	0-15	HM	2 DR	SW	CNT
DRAW	0-15	HM	2 DR	FT	PRS
DRAW	0-15	HM	2 DR	FT	CNT
DRAW	16-30	HM	2 DR	FT	CNT
DRAW	16-30	HM	2 DR	SW	PRS
DRAW	16-30	HM	2 L1	SW	PRS
DRAW	31-45+	HM	2 L1	FT	PRS
DRAW	31-45+	HM	2 L1	SW	CNT
DRAW	31-45+	HM	2 L1	SW	CNT
DRAW	31-45+	HM	2 L1	SW	PRS
DRAW	31-45+	HM	2 L1	SW	PRS
DRAW	31-45+	HM	2 L1	FT	CNT
DRAW	31-45+	HM	2 L1	SW	CNT
DRAW	31-45+	HM	2 L1	FT	CNT
DRAW	31-45+	HM	2 L1	FT	PRS
DRAW	31-45+	HM	2 L1	SW	PRS
DRAW	46-60	HM	2 L1	SW	CNT
DRAW	46-60	HM	2 L1	SW	PRS
DRAW	46-60	HM	2 L1	SW	CNT
DRAW	46-60	HM	2 L1	FT	CNT
DRAW	46-60	HM	2 L1	FT	CNT
DRAW	46-60	HM	2 L1	FT	CNT
DRAW	61-75	HM	2 L1	FT	CNT
DRAW	61-75	HM	2 L1	FT	CNT
DRAW	61-75	HM	2 L1	SW	CNT
DRAW	61-75	HM	2 L1	FT	CNT
DRAW	61-75	HM	2 DR	SW	CNT
DRAW	61-75	HM	2 DR	FT	CNT
DRAW	76-90+	HM	2 DR	SW	CNT
DRAW	76-90+	HM	2 DR	FT	CNT
DRAW	76-90+	HM	2 DR	SW	PRS
LOSS	0-15	AW	2 DR	SW	PRS

LOSS	0-15	AW	2 DR	SW	PRS
LOSS	0-15	AW	2 DR	FT	PRS
LOSS	0-15	AW	2 DR	SW	PRS
LOSS	16-30	AW	2 DR	SW	PRS
LOSS	16-30	AW	2 L1	SW	PRS
LOSS	31-45+	AW	2 L1	SW	PRS
LOSS	31-45+	AW	2 L1	SW	PRS
LOSS	31-45+	AW	2 L1	FT	PRS
LOSS	31-45+	AW	2 L1	FT	PRS
LOSS	31-45+	AW	2 L1	SW	PRS
LOSS	46-60	AW	2 L1	SW	PRS
LOSS	61-75	AW	2 L2	SW	PRS
LOSS	76-90+	AW	2 L2	SW	PRS
LOSS	76-90+	AW	2 L2	FT	PRS
LOSS	0-15	AW	2 DR	SW	CNT
LOSS	0-15	AW	2 DR	SW	CNT
LOSS	16-30	AW	2 DR	SW	PRS
LOSS	16-30	AW	2 DR	SW	CNT
LOSS	31-45+	AW	2 L1	SW	CNT
LOSS	31-45+	AW	2 L1	SW	PRS
LOSS	31-45+	AW	2 L1	SW	CNT
LOSS	61-75	AW	2 L1	SW	PRS
LOSS	61-75	AW	2 L1	SW	PRS
LOSS	61-75	AW	2 L1	SW	PRS
LOSS	61-75	AW	2 L1	SW	PRS
LOSS	76-90+	AW	2 L1	SW	CNT
LOSS	0-15	AW	2 DR	SW	PRS
LOSS	0-15	AW	2 DR	SW	PRS
LOSS	0-15	AW	2 DR	SW	PRS
LOSS	0-15	AW	2 DR	SW	PRS
LOSS	0-15	AW	2 DR	FT	CNT
LOSS	0-15	HM	2 DR	SW	PRS
LOSS	16-30	AW	2 L2	SW	PRS
LOSS	16-30	AW	2 L2	SW	PRS
LOSS	16-30	AW	2 L2	SW	PRS
LOSS	16-30	AW	2 L2	SW	PRS
LOSS	31-45+	AW	2 L2	SW	PRS
LOSS	31-45+	AW	2 L2	SW	PRS
LOSS	46-60	AW	2 L2	SW	PRS
LOSS	46-60	AW	2 L2	SW	CNT
LOSS	46-60	AW	2 L2	SW	PRS
LOSS	61-75	AW	2 DR	SW	PRS
LOSS	61-75	AW	2 DR	SW	PRS
LOSS	61-75	AW	2 DR	SW	PRS
LOSS	61-75	AW	2 DR	SW	PRS
LOSS	76-90+	AW	2 DR	SW	PRS
LOSS	76-90+	AW	2 L1	SW	PRS
LOSS	76-90+	AW	2 L1	SW	CNT
WIN	0-15	HM	2 DR	SW	CNT
WIN	0-15	HM	2 DR	SW	PRS

WIN	0-15	HM	2	DR	SW	PRS
WIN	0-15	HM	2	W1	FT	PRS
WIN	16-30	HM	2	W1	SW	PRS
WIN	16-30	HM	2	W1	SW	PRS
WIN	16-30	HM	2	W1	SW	PRS
WIN	16-30	HM	2	W1	SW	PRS
WIN	16-30	HM	2	W1	SW	PRS
WIN	31-45+	HM	2	W1	SW	CNT
WIN	31-45+	HM	2	W1	SW	PRS
WIN	31-45+	HM	2	W1	FT	PRS
WIN	46-60	HM	2	W1	SW	PRS
WIN	76-90+	HM	2	W2	SW	PRS
WIN	76-90+	HM	2	W2	SW	PRS
WIN	76-90+	HM	2	W2	SW	PRS
WIN	76-90+	HM	2	W1	SW	PRS
WIN	0-15	HM	2	DR	SW	CNT
WIN	0-15	HM	2	DR	SW	PRS
WIN	0-15	HM	2	DR	SW	CNT
WIN	0-15	HM	2	DR	SW	PRS
WIN	0-15	HM	2	DR	SW	PRS
WIN	16-30	HM	2	L1	SW	CNT
WIN	16-30	HM	2	L1	FT	PRS
WIN	16-30	HM	2	L1	SW	PRS
WIN	31-45+	HM	2	L1	SW	PRS
WIN	31-45+	HM	2	L1	SW	PRS
WIN	31-45+	HM	2	L1	FT	PRS
WIN	31-45+	HM	2	L1	FT	PRS
WIN	31-45+	HM	2	L1	FT	PRS
WIN	46-60	HM	2	L1	SW	PRS
WIN	46-60	HM	2	L1	FT	PRS
WIN	46-60	HM	2	DR	FT	PRS
WIN	61-75	HM	2	W1	SW	PRS
WIN	61-75	HM	2	W1	SW	PRS
WIN	61-75	HM	2	W1	SW	PRS
WIN	61-75	HM	2	W1	SW	PRS
WIN	76-90+	HM	2	W1	SW	PRS
WIN	76-90+	HM	2	W1	SW	PRS
WIN	76-90+	HM	2	W1	SW	PRS
DRAW	0-15	HM	2	DR	FT	PRS
DRAW	0-15	HM	2	DR	FT	PRS
DRAW	16-30	HM	2	W1	SW	PRS
DRAW	16-30	HM	2	W1	FT	PRS
DRAW	16-30	HM	2	W1	SW	CNT
DRAW	46-60	HM	2	W1	SW	PRS
DRAW	61-75	HM	2	L1	FT	PRS
DRAW	61-75	HM	2	L1	FT	PRS
DRAW	76-90+	HM	2	L1	FT	PRS
DRAW	76-90+	HM	2	DR	FT	CNT
DRAW	76-90+	HM	2	DR	FT	CNT
DRAW	0-15	HM	2	DR	SW	PRS

DRAW	0-15	HM	2	DR	SW	PRS
DRAW	0-15	HM	2	DR	FT	CNT
DRAW	16-30	HM	2	DR	SW	PRS
DRAW	31-45+	HM	2	W1	SW	PRS
DRAW	31-45+	HM	2	W1	SW	PRS
DRAW	31-45+	HM	2	W1	SW	PRS
DRAW	46-60	HM	2	W1	SW	PRS
DRAW	46-60	HM	2	DR	SW	PRS
DRAW	61-75	HM	2	DR	SW	PRS
DRAW	61-75	HM	2	DR	FT	PRS
DRAW	76-90+	HM	2	DR	SW	PRS
DRAW	76-90+	HM	2	DR	SW	PRS
DRAW	76-90+	HM	2	DR	FT	PRS
LOSS	0-15	HM	2	W1	SW	PRS
LOSS	46-60	HM	2	L1	SW	PRS
LOSS	46-60	HM	2	L1	FT	PRS
LOSS	46-60	HM	2	L1	FT	PRS
LOSS	46-60	HM	2	L1	SW	PRS
LOSS	61-75	HM	2	L1	SW	PRS
LOSS	61-75	HM	2	L1	FT	PRS
LOSS	61-75	HM	2	L1	SW	PRS
LOSS	61-75	HM	2	L1	SW	PRS
LOSS	76-90+	HM	2	L1	FT	CNT
LOSS	76-90+	HM	2	L1	FT	PRS
LOSS	76-90+	HM	2	L1	FT	CNT
LOSS	76-90+	HM	2	L1	SW	PRS
LOSS	76-90+	HM	2	L1	SW	PRS
LOSS	76-90+	HM	2	L1	SW	PRS
LOSS	76-90+	HM	2	L1	FT	PRS
WIN	0-15	AW	2	DR	SW	PRS
WIN	0-15	AW	2	DR	SW	PRS
WIN	16-30	AW	2	W1	SW	PRS
WIN	31-45+	AW	2	W1	SW	PRS
WIN	31-45+	AW	2	W1	SW	PRS
WIN	31-45+	AW	2	W1	SW	PRS
WIN	31-45+	AW	2	W1	SW	PRS
WIN	61-75	AW	2	W1	SW	PRS
WIN	61-75	AW	2	W1	SW	PRS
WIN	76-90+	AW	2	W1	SW	PRS
WIN	76-90+	AW	2	W1	SW	PRS
LOSS	0-15	AW	3	DR	SW	CNT
LOSS	0-15	AW	3	DR	SW	PRS
LOSS	0-15	AW	3	DR	SW	CNT
LOSS	16-30	AW	3	DR	SW	CNT
LOSS	16-30	AW	3	DR	SW	CNT
LOSS	16-30	AW	3	DR	SW	PRS
LOSS	31-45+	AW	3	DR	SW	CNT
LOSS	31-45+	AW	3	DR	SW	CNT
LOSS	31-45+	AW	3	DR	SW	PRS
LOSS	46-60	AW	3	DR	SW	PRS

LOSS	46-60	AW	3	L1	SW	CNT
LOSS	61-75	AW	3	L1	SW	CNT
LOSS	76-90+	AW	3	DR	SW	CNT
LOSS	0-15	AW	3	DR	SW	PRS
LOSS	16-30	AW	3	DR	SW	PRS
LOSS	31-45+	AW	3	DR	SW	PRS
LOSS	31-45+	AW	3	DR	SW	PRS
LOSS	46-60	AW	3	DR	SW	PRS
LOSS	46-60	AW	3	DR	SW	PRS
LOSS	46-60	AW	3	DR	SW	PRS
LOSS	46-60	AW	3	DR	SW	PRS
LOSS	61-75	AW	3	DR	SW	PRS
LOSS	61-75	AW	3	DR	SW	PRS
LOSS	76-90+	AW	3	DR	SW	PRS
DRAW	0-15	AW	3	DR	SW	PRS
DRAW	0-15	AW	3	DR	SW	PRS
DRAW	16-30	AW	3	DR	SW	PRS
DRAW	16-30	AW	3	DR	SW	PRS
DRAW	31-45+	AW	3	L1	FT	PRS
DRAW	31-45+	AW	3	L1	SW	PRS
DRAW	31-45+	AW	3	L1	FT	PRS
DRAW	31-45+	AW	3	L1	SW	PRS
DRAW	46-60	AW	3	L1	SW	PRS
DRAW	61-75	AW	3	W1	SW	PRS
DRAW	61-75	AW	3	W1	SW	PRS
DRAW	76-90+	AW	3	W1	SW	PRS
DRAW	76-90+	AW	3	DR	SW	PRS
DRAW	0-15	AW	3	DR	FT	PRS
DRAW	16-30	AW	3	DR	SW	PRS
DRAW	16-30	AW	3	DR	SW	PRS
DRAW	31-45+	AW	3	DR	SW	PRS
DRAW	31-45+	AW	3	DR	SW	PRS
DRAW	31-45+	AW	3	DR	SW	PRS
DRAW	31-45+	AW	3	DR	SW	PRS
DRAW	31-45+	AW	3	DR	SW	PRS
DRAW	46-60	AW	3	DR	SW	PRS
DRAW	46-60	AW	3	DR	SW	PRS
DRAW	61-75	AW	3	DR	FT	PRS
DRAW	76-90+	AW	3	DR	SW	PRS
DRAW	76-90+	AW	3	DR	SW	PRS
LOSS	0-15	AW	3	DR	SW	PRS
LOSS	0-15	AW	3	DR	SW	CNT
LOSS	16-30	AW	3	DR	SW	PRS
LOSS	31-45+	AW	3	DR	SW	PRS
LOSS	31-45+	AW	3	W2	FT	PRS
LOSS	46-60	AW	3	L2	FT	PRS
LOSS	76-90+	AW	3	L>2	SW	CNT
LOSS	76-90+	AW	3	L>2	SW	PRS
LOSS	76-90+	AW	3	L>2	SW	PRS
DRAW	0-15	AW	3	DR	SW	CNT

DRAW	0-15	AW	3 DR	SW	PRS
DRAW	0-15	AW	3 W1	SW	PRS
DRAW	16-30	AW	3 W1	SW	PRS
DRAW	16-30	AW	3 W1	SW	PRS
DRAW	16-30	AW	3 W1	SW	PRS
DRAW	31-45+	AW	3 W1	SW	PRS
DRAW	31-45+	AW	3 W1	SW	PRS
DRAW	46-60	AW	3 DR	SW	PRS
DRAW	46-60	AW	3 DR	SW	PRS
DRAW	61-75	AW	3 DR	SW	PRS
DRAW	61-75	AW	3 DR	SW	CNT
DRAW	61-75	AW	3 DR	SW	PRS
DRAW	61-75	AW	3 DR	SW	PRS
DRAW	61-75	AW	3 DR	SW	PRS
DRAW	61-75	AW	3 DR	SW	CNT
DRAW	61-75	AW	3 DR	SW	PRS
DRAW	76-90+	AW	3 DR	SW	PRS
DRAW	76-90+	AW	3 DR	SW	PRS
DRAW	76-90+	AW	3 DR	SW	PRS
LOSS	0-15	AW	3 DR	SW	PRS
LOSS	16-30	AW	3 DR	SW	PRS
LOSS	16-30	AW	3 DR	SW	PRS
LOSS	16-30	AW	3 DR	FT	PRS
LOSS	16-30	AW	3 DR	SW	PRS
LOSS	16-30	AW	3 DR	SW	PRS
LOSS	16-30	AW	3 DR	SW	PRS
LOSS	16-30	AW	3 DR	SW	PRS
LOSS	31-45+	AW	3 DR	SW	PRS
LOSS	46-60	AW	3 L1	FT	PRS
LOSS	61-75	AW	3 L1	SW	PRS
LOSS	61-75	AW	3 L1	SW	PRS
LOSS	76-90+	AW	3 L2	SW	PRS
LOSS	76-90+	AW	3 L2	SW	PRS
LOSS	76-90+	AW	3 L2	SW	PRS
LOSS	76-90+	AW	3 L2	FT	PRS
LOSS	76-90+	AW	3 L2	SW	PRS
LOSS	0-15	AW	3 L1	SW	PRS
LOSS	0-15	AW	3 L1	FT	PRS
LOSS	16-30	AW	3 L1	SW	PRS
LOSS	16-30	AW	3 L1	SW	PRS
LOSS	16-30	AW	3 L1	SW	PRS
LOSS	16-30	AW	3 L1	SW	PRS
LOSS	16-30	AW	3 L1	SW	PRS
LOSS	31-45+	AW	3 L1	SW	PRS
LOSS	31-45+	AW	3 L1	FT	PRS
LOSS	31-45+	AW	3 L1	SW	PRS
LOSS	31-45+	AW	3 L1	SW	PRS
LOSS	31-45+	AW	3 L1	SW	PRS
LOSS	31-45+	AW	3 L1	SW	PRS
LOSS	31-45+	AW	3 L1	SW	CNT

LOSS	46-60	AW	3	L1	FT	PRS
LOSS	46-60	AW	3	L1	SW	PRS
LOSS	46-60	AW	3	L1	SW	PRS
LOSS	46-60	AW	3	L1	FT	PRS
LOSS	46-60	AW	3	L1	SW	PRS
LOSS	61-75	AW	3	L1	SW	PRS
LOSS	61-75	AW	3	L1	FT	PRS
LOSS	61-75	AW	3	L1	SW	PRS
LOSS	76-90+	AW	3	L2	SW	PRS
LOSS	76-90+	AW	3	L>2	SW	PRS
LOSS	76-90+	AW	3	L>2	FT	PRS
LOSS	16-30	AW	3	DR	SW	PRS
LOSS	16-30	AW	3	L1	SW	PRS
LOSS	31-45+	AW	3	L1	SW	PRS
LOSS	31-45+	AW	3	L1	SW	PRS
LOSS	46-60	AW	3	L1	SW	PRS
LOSS	46-60	AW	3	L1	SW	PRS
LOSS	46-60	AW	3	L1	FT	PRS
LOSS	46-60	AW	3	L1	SW	PRS
LOSS	61-75	AW	3	L1	SW	PRS
LOSS	61-75	AW	3	L1	FT	PRS
LOSS	76-90+	AW	3	L1	SW	PRS
DRAW	0-15	AW	3	DR	SW	CNT
DRAW	16-30	AW	3	DR	SW	PRS
DRAW	31-45+	AW	3	DR	FT	CNT
DRAW	31-45+	AW	3	DR	SW	PRS
DRAW	31-45+	AW	3	DR	SW	PRS
DRAW	46-60	AW	3	DR	FT	CNT
DRAW	46-60	AW	3	DR	FT	CNT
DRAW	46-60	AW	3	DR	SW	PRS
DRAW	61-75	AW	3	DR	SW	PRS
DRAW	76-90+	AW	3	DR	SW	CNT
DRAW	76-90+	AW	3	DR	SW	PRS
LOSS	0-15	AW	3	DR	SW	PRS
LOSS	0-15	AW	3	DR	SW	CNT
LOSS	0-15	AW	3	DR	SW	PRS
LOSS	0-15	AW	3	DR	SW	PRS
LOSS	0-15	AW	3	DR	SW	PRS
LOSS	16-30	AW	3	DR	SW	PRS
LOSS	16-30	AW	3	DR	SW	PRS
LOSS	31-45+	AW	3	DR	SW	PRS
LOSS	31-45+	AW	3	DR	SW	PRS
LOSS	31-45+	AW	3	DR	SW	PRS
LOSS	46-60	AW	3	DR	SW	PRS
LOSS	46-60	AW	3	DR	SW	PRS
LOSS	46-60	AW	3	DR	SW	PRS
LOSS	61-75	AW	3	DR	SW	PRS
LOSS	76-90+	AW	3	DR	SW	PRS
LOSS	76-90+	AW	3	DR	SW	PRS
LOSS	76-90+	AW	3	DR	SW	PRS

LOSS	76-90+	AW	3	L1	SW	PRS
LOSS	76-90+	AW	3	L1	SW	PRS
WIN	0-15	HM	3	L1	SW	PRS
WIN	0-15	HM	3	L1	SW	PRS
WIN	16-30	HM	3	DR	SW	PRS
WIN	31-45+	HM	3	DR	SW	PRS
WIN	46-60	HM	3	DR	SW	PRS
WIN	46-60	HM	3	DR	FT	PRS
WIN	46-60	HM	3	DR	FT	CNT
WIN	61-75	HM	3	W1	SW	PRS
WIN	61-75	HM	3	W1	SW	PRS
WIN	76-90+	HM	3	W1	SW	PRS
WIN	76-90+	HM	3	W1	SW	PRS
WIN	76-90+	HM	3	W1	SW	PRS
LOSS	0-15	HM	3	DR	SW	PRS
LOSS	16-30	HM	3	W1	SW	CNT
LOSS	16-30	HM	3	W1	SW	PRS
LOSS	31-45+	HM	3	W1	SW	PRS
LOSS	31-45+	HM	3	W1	SW	PRS
LOSS	31-45+	HM	3	W1	SW	PRS
LOSS	31-45+	HM	3	W1	SW	PRS
LOSS	31-45+	HM	3	W1	SW	PRS
LOSS	46-60	HM	3	W1	SW	PRS
LOSS	46-60	HM	3	DR	SW	PRS
LOSS	61-75	HM	3	L1	SW	PRS
LOSS	61-75	HM	3	L1	SW	PRS
LOSS	61-75	HM	3	L1	FT	CNT
LOSS	76-90+	HM	3	DR	SW	PRS
LOSS	76-90+	HM	3	DR	SW	PRS
LOSS	76-90+	HM	3	DR	SW	PRS
LOSS	76-90+	HM	3	L1	SW	PRS
LOSS	76-90+	HM	3	L1	FT	CNT
LOSS	0-15	HM	3	DR	FT	CNT
LOSS	0-15	HM	3	DR	FT	CNT
LOSS	0-15	HM	3	L1	SW	PRS
LOSS	16-30	HM	3	L1	SW	PRS
LOSS	16-30	HM	3	L1	SW	PRS
LOSS	16-30	HM	3	L1	FT	PRS
LOSS	46-60	HM	3	L1	FT	CNT
LOSS	46-60	HM	3	L1	SW	CNT
LOSS	46-60	HM	3	L1	SW	PRS
LOSS	61-75	HM	3	L1	SW	PRS
LOSS	61-75	HM	3	L1	SW	CNT
LOSS	61-75	HM	3	L1	FT	CNT
LOSS	76-90+	HM	3	L1	SW	PRS
LOSS	76-90+	HM	3	L1	SW	CNT
LOSS	76-90+	HM	3	L1	FT	PRS
LOSS	76-90+	HM	3	L1	FT	PRS
LOSS	0-15	HM	3	DR	SW	PRS
LOSS	0-15	HM	3	DR	SW	PRS

LOSS	16-30	HM	3	DR	SW	PRS
LOSS	16-30	HM	3	DR	SW	PRS
LOSS	16-30	HM	3	DR	SW	CNT
LOSS	31-45+	HM	3	L2	FT	CNT
LOSS	31-45+	HM	3	L2	SW	PRS
LOSS	46-60	HM	3	L2	FT	PRS
LOSS	61-75	HM	3	L1	SW	CNT
LOSS	61-75	HM	3	L1	SW	PRS
LOSS	76-90+	HM	3	L2	SW	PRS
LOSS	76-90+	HM	3	L2	FT	PRS
LOSS	76-90+	HM	3	L2	SW	PRS
LOSS	0-15	AW	3	DR	SW	PRS
LOSS	0-15	AW	3	DR	SW	PRS
LOSS	0-15	AW	3	DR	SW	PRS
LOSS	16-30	AW	3	DR	SW	PRS
LOSS	16-30	AW	3	DR	SW	PRS
LOSS	16-30	AW	3	DR	SW	PRS
LOSS	16-30	AW	3	W1	SW	PRS
LOSS	16-30	AW	3	W1	SW	PRS
LOSS	16-30	AW	3	W1	SW	PRS
LOSS	31-45+	AW	3	DR	SW	PRS
LOSS	46-60	AW	3	DR	SW	PRS
LOSS	46-60	AW	3	DR	SW	PRS
LOSS	46-60	AW	3	DR	SW	PRS
LOSS	61-75	AW	3	L1	SW	PRS
LOSS	76-90+	AW	3	L1	SW	PRS
LOSS	76-90+	AW	3	L1	SW	PRS
LOSS	76-90+	AW	3	L1	SW	PRS
DRAW	0-15	AW	3	DR	FT	CNT
DRAW	0-15	AW	3	DR	FT	CNT
DRAW	0-15	AW	3	DR	SW	CNT
DRAW	16-30	AW	3	DR	FT	CNT
DRAW	16-30	AW	3	DR	SW	PRS
DRAW	16-30	AW	3	DR	SW	PRS
DRAW	46-60	AW	3	W1	SW	PRS
DRAW	46-60	AW	3	W1	SW	PRS
DRAW	46-60	AW	3	W1	SW	PRS
DRAW	76-90+	AW	3	W1	SW	PRS
DRAW	76-90+	AW	3	W1	SW	PRS
DRAW	76-90+	AW	3	W1	SW	PRS
DRAW	76-90+	AW	3	W1	SW	PRS
DRAW	0-15	AW	3	DR	SW	PRS
DRAW	16-30	AW	3	DR	SW	PRS
DRAW	16-30	AW	3	DR	SW	PRS
DRAW	16-30	AW	3	DR	SW	PRS
DRAW	16-30	AW	3	DR	SW	PRS
DRAW	31-45+	AW	3	DR	SW	PRS
DRAW	31-45+	AW	3	DR	SW	PRS
DRAW	31-45+	AW	3	DR	SW	PRS
DRAW	31-45+	AW	3	DR	SW	PRS

DRAW	31-45+	AW	3	DR	SW	PRS
DRAW	46-60	AW	3	DR	SW	PRS
DRAW	46-60	AW	3	DR	SW	PRS
DRAW	46-60	AW	3	DR	SW	PRS
DRAW	61-75	AW	3	DR	SW	PRS
DRAW	61-75	AW	3	L1	SW	CNT
DRAW	76-90+	AW	3	L1	FT	CNT
DRAW	76-90+	AW	3	L1	SW	CNT
DRAW	76-90+	AW	3	DR	FT	PRS
DRAW	76-90+	AW	3	DR	SW	PRS
LOSS	0-15	AW	3	DR	SW	CNT
LOSS	16-30	AW	3	DR	SW	CNT
LOSS	16-30	AW	3	DR	SW	CNT
LOSS	31-45+	AW	3	DR	SW	PRS
LOSS	31-45+	AW	3	DR	SW	CNT
LOSS	31-45+	AW	3	DR	SW	CNT
LOSS	31-45+	AW	3	DR	SW	CNT
LOSS	31-45+	AW	3	DR	SW	CNT
LOSS	46-60	AW	3	DR	SW	CNT
LOSS	46-60	AW	3	DR	SW	CNT
LOSS	46-60	AW	3	DR	SW	CNT
LOSS	61-75	AW	3	DR	SW	CNT
LOSS	61-75	AW	3	L1	SW	CNT
LOSS	61-75	AW	3	L1	SW	PRS
LOSS	61-75	AW	3	L1	SW	CNT
LOSS	61-75	AW	3	L1	SW	CNT
LOSS	46-60	AW	3	L1	SW	CNT
LOSS	61-75	AW	3	L1	SW	PRS
LOSS	76-90+	AW	3	L2	SW	CNT
LOSS	76-90+	AW	3	L2	SW	CNT
LOSS	76-90+	AW	3	L>2	SW	CNT
LOSS	0-15	AW	3	DR	SW	PRS
LOSS	0-15	AW	3	DR	SW	PRS
LOSS	16-30	AW	3	W1	SW	PRS
LOSS	16-30	AW	3	W1	SW	PRS
LOSS	16-30	AW	3	W1	SW	PRS
LOSS	31-45+	AW	3	W1	SW	CNT
LOSS	31-45+	AW	3	W1	SW	PRS
LOSS	46-60	AW	3	W1	SW	PRS
LOSS	46-60	AW	3	W1	SW	PRS
LOSS	46-60	AW	3	W1	SW	PRS
LOSS	61-75	AW	3	L1	SW	CNT
LOSS	76-90+	AW	3	L2	SW	PRS
LOSS	76-90+	AW	3	L2	FT	CNT
DRAW	0-15	AW	3	DR	FT	CNT
DRAW	0-15	AW	3	DR	FT	CNT
DRAW	0-15	AW	3	DR	SW	PRS
DRAW	0-15	AW	3	DR	SW	PRS
DRAW	16-30	AW	3	DR	SW	CNT
DRAW	16-30	AW	3	DR	SW	PRS

DRAW	31-45+	AW	3	DR	SW	PRS
DRAW	31-45+	AW	3	L1	SW	CNT
DRAW	31-45+	AW	3	L1	SW	PRS
DRAW	46-60	AW	3	L1	SW	PRS
DRAW	46-60	AW	3	L1	FT	PRS
DRAW	46-60	AW	3	L1	SW	PRS
DRAW	61-75	AW	3	DR	SW	PRS
DRAW	76-90+	AW	3	DR	SW	PRS
DRAW	76-90+	AW	3	DR	SW	PRS
DRAW	76-90+	AW	3	DR	SW	PRS
DRAW	0-15	HM	3	DR	SW	PRS
DRAW	0-15	HM	3	DR	SW	PRS
DRAW	0-15	HM	3	DR	SW	PRS
DRAW	0-15	HM	3	DR	FT	PRS
DRAW	16-30	HM	3	DR	FT	CNT
DRAW	16-30	HM	3	DR	SW	CNT
DRAW	16-30	HM	3	DR	SW	PRS
DRAW	16-30	HM	3	DR	SW	PRS
DRAW	31-45+	HM	3	DR	FT	PRS
DRAW	46-60	HM	3	DR	SW	PRS
DRAW	46-60	HM	3	DR	FT	PRS
DRAW	46-60	HM	3	DR	SW	PRS
DRAW	46-60	HM	3	DR	SW	PRS
DRAW	76-90+	HM	3	DR	SW	PRS
DRAW	76-90+	HM	3	DR	FT	PRS
DRAW	76-90+	HM	3	DR	SW	PRS
LOSS	0-15	AW	3	DR	SW	PRS
LOSS	0-15	AW	3	L1	SW	PRS
LOSS	0-15	AW	3	L1	SW	PRS
LOSS	0-15	AW	3	L1	FT	PRS
LOSS	0-15	AW	3	L1	SW	PRS
LOSS	16-30	AW	3	L1	SW	PRS
LOSS	16-30	AW	3	L1	SW	PRS
LOSS	16-30	AW	3	L1	SW	PRS
LOSS	31-45+	AW	3	L2	SW	PRS
LOSS	31-45+	AW	3	L2	SW	PRS
LOSS	46-60	AW	3	L>2	SW	PRS
LOSS	46-60	AW	3	L2	SW	PRS
LOSS	61-75	AW	3	L2	SW	PRS
LOSS	76-90+	AW	3	L2	SW	PRS
LOSS	76-90+	AW	3	L2	SW	PRS
LOSS	0-15	AW	3	DR	SW	PRS
LOSS	0-15	AW	3	DR	SW	CNT
LOSS	16-30	AW	3	DR	SW	PRS
LOSS	31-45+	AW	3	L1	SW	PRS
LOSS	46-60	AW	3	L1	SW	PRS
LOSS	46-60	AW	3	L1	SW	PRS
LOSS	46-60	AW	3	L1	SW	PRS
LOSS	46-60	AW	3	L1	SW	CNT
LOSS	46-60	AW	3	L2	SW	CNT

LOSS	46-60	AW	3	L2	SW	PRS
LOSS	61-75	AW	3	L2	SW	PRS
LOSS	76-90+	AW	3	L1	SW	PRS
LOSS	76-90+	AW	3	L1	FT	PRS
LOSS	76-90+	AW	3	L1	SW	PRS
DRAW	0-15	AW	3	DR	SW	PRS
DRAW	0-15	AW	3	DR	SW	PRS
DRAW	16-30	AW	3	DR	SW	PRS
DRAW	31-45+	AW	3	L1	FT	PRS
DRAW	31-45+	AW	3	L1	SW	PRS
DRAW	46-60	AW	3	L1	SW	PRS
DRAW	46-60	AW	3	L1	SW	PRS
DRAW	61-75	AW	3	L1	SW	PRS
DRAW	61-75	AW	3	L1	SW	CNT
DRAW	76-90+	AW	3	L1	SW	PRS
DRAW	76-90+	AW	3	L1	SW	CNT
DRAW	76-90+	AW	3	L1	SW	PRS
LOSS	0-15	AW	3	L1	SW	PRS
LOSS	0-15	AW	3	L1	SW	PRS
LOSS	16-30	AW	3	L1	SW	PRS
LOSS	16-30	AW	3	L1	SW	PRS
LOSS	31-45+	AW	3	L2	SW	PRS
LOSS	31-45+	AW	3	L2	SW	PRS
LOSS	31-45+	AW	3	L2	FT	PRS
LOSS	31-45+	AW	3	L2	SW	PRS
LOSS	46-60	AW	3	L2	FT	PRS
LOSS	46-60	AW	3	L>2	SW	PRS
LOSS	46-60	AW	3	L>2	SW	PRS
LOSS	61-75	AW	3	L>2	FT	PRS
LOSS	61-75	AW	3	L>2	FT	PRS
LOSS	76-90+	AW	3	L>2	FT	PRS
LOSS	0-15	AW	3	DR	SW	PRS
LOSS	0-15	AW	3	DR	SW	PRS
LOSS	16-30	AW	3	L1	SW	PRS
LOSS	16-30	AW	3	L1	SW	PRS
LOSS	16-30	AW	3	L1	SW	PRS
LOSS	16-30	AW	3	L2	SW	PRS
LOSS	16-30	AW	3	L2	SW	PRS
LOSS	31-45+	AW	3	L2	SW	PRS
LOSS	31-45+	AW	3	L2	SW	PRS
LOSS	31-45+	AW	3	L2	SW	PRS
LOSS	31-45+	AW	3	L2	FT	PRS
LOSS	46-60	AW	3	L2	SW	PRS
LOSS	46-60	AW	3	L2	FT	PRS
LOSS	46-60	AW	3	L2	SW	PRS
LOSS	46-60	AW	3	L2	SW	PRS
LOSS	61-75	AW	3	L>2	FT	PRS
LOSS	61-75	AW	3	L>2	FT	PRS
LOSS	76-90+	AW	3	L>2	FT	PRS
LOSS	76-90+	AW	3	L>2	FT	PRS

LOSS	76-90+	AW	3	L>2	FT	PRS
LOSS	76-90+	AW	3	L>2	FT	PRS
LOSS	76-90+	AW	3	L2	SW	PRS
LOSS	0-15	AW	2	DR	SW	CNT
LOSS	0-15	AW	2	DR	SW	CNT
LOSS	0-15	AW	2	DR	SW	PRS
LOSS	0-15	AW	2	DR	SW	CNT
LOSS	16-30	AW	2	DR	FT	CNT
LOSS	16-30	AW	2	DR	SW	PRS
LOSS	16-30	AW	2	DR	SW	PRS
LOSS	16-30	AW	2	DR	SW	PRS
LOSS	16-30	AW	2	DR	SW	PRS
LOSS	16-30	AW	2	DR	SW	PRS
LOSS	31-45+	AW	2	DR	SW	CNT
LOSS	31-45+	AW	2	DR	SW	PRS
LOSS	31-45+	AW	2	DR	SW	PRS
LOSS	31-45+	AW	2	DR	FT	CNT
LOSS	31-45+	AW	2	DR	SW	CNT
LOSS	31-45+	AW	2	DR	SW	PRS
LOSS	46-60	AW	2	DR	SW	PRS
LOSS	61-75	AW	2	DR	SW	CNT
LOSS	76-90+	AW	2	DR	SW	PRS
LOSS	76-90+	AW	2	DR	SW	PRS
LOSS	76-90+	AW	2	DR	SW	PRS
LOSS	76-90+	AW	2	L1	SW	PRS
LOSS	16-30	AW	2	DR	SW	PRS
LOSS	16-30	AW	2	DR	SW	PRS
LOSS	31-45+	AW	2	L1	SW	CNT
LOSS	31-45+	AW	2	L1	SW	CNT
LOSS	46-60	AW	2	L1	FT	CNT
LOSS	46-60	AW	2	L1	FT	CNT
LOSS	46-60	AW	2	L1	SW	PRS
LOSS	46-60	AW	2	L1	SW	CNT
LOSS	46-60	AW	2	L1	SW	CNT
LOSS	46-60	AW	2	L1	SW	PRS
LOSS	61-75	AW	2	L1	SW	CNT
LOSS	61-75	AW	2	L1	SW	CNT
LOSS	76-90+	AW	2	L2	SW	PRS
LOSS	76-90+	AW	2	L2	FT	PRS
LOSS	76-90+	AW	2	L2	FT	CNT
LOSS	0-15	AW	2	DR	SW	PRS
LOSS	0-15	AW	2	DR	SW	CNT
LOSS	16-30	AW	2	DR	SW	PRS
LOSS	16-30	AW	2	DR	SW	CNT
LOSS	16-30	AW	2	DR	SW	PRS
LOSS	16-30	AW	2	DR	FT	PRS
LOSS	16-30	AW	2	DR	SW	PRS
LOSS	16-30	AW	2	DR	SW	CNT
LOSS	16-30	AW	2	DR	SW	CNT
LOSS	31-45+	AW	2	DR	SW	PRS

LOSS	31-45+	AW	2	DR	SW	PRS
LOSS	31-45+	AW	2	L1	SW	PRS
LOSS	46-60	AW	2	L2	SW	CNT
LOSS	46-60	AW	2	L1	SW	PRS
LOSS	46-60	AW	2	L2	SW	PRS
LOSS	46-60	AW	2	L2	SW	PRS
LOSS	61-75	AW	2	L2	FT	CNT
LOSS	61-75	AW	2	L1	SW	PRS
LOSS	61-75	AW	2	L1	SW	PRS
LOSS	76-90+	AW	2	L1	SW	PRS
LOSS	76-90+	AW	2	L2	SW	PRS
LOSS	0-15	HM	2	L1	FT	PRS
LOSS	0-15	HM	2	L1	SW	PRS
LOSS	0-15	HM	2	L1	FT	CNT
LOSS	16-30	HM	2	L1	SW	PRS
LOSS	16-30	HM	2	L1	SW	PRS
LOSS	31-45+	HM	2	L1	SW	PRS
LOSS	31-45+	HM	2	L1	SW	CNT
LOSS	31-45+	HM	2	L1	SW	PRS
LOSS	31-45+	HM	2	L1	SW	PRS
LOSS	46-60	HM	2	L1	FT	PRS
LOSS	46-60	HM	2	L1	FT	PRS
LOSS	46-60	HM	2	L1	FT	PRS
LOSS	46-60	HM	2	L1	FT	PRS
LOSS	46-60	HM	2	L1	FT	PRS
LOSS	61-75	HM	2	L2	SW	PRS
LOSS	61-75	HM	2	L2	FT	CNT
LOSS	76-90+	HM	2	L2	SW	CNT
LOSS	76-90+	HM	2	L2	SW	CNT
LOSS	76-90+	HM	2	L2	FT	CNT
LOSS	76-90+	HM	2	L>2	FT	CNT
LOSS	0-15	HM	2	DR	SW	PRS
LOSS	0-15	HM	2	DR	SW	PRS
LOSS	0-15	HM	2	DR	SW	PRS
LOSS	0-15	HM	2	DR	SW	PRS
LOSS	0-15	HM	2	DR	SW	PRS
LOSS	0-15	HM	2	DR	SW	PRS
LOSS	0-15	HM	2	DR	FT	PRS
LOSS	0-15	HM	2	DR	SW	PRS
LOSS	0-15	HM	2	DR	FT	CNT
LOSS	16-30	HM	2	DR	SW	PRS
LOSS	16-30	HM	2	L1	SW	PRS
LOSS	16-30	HM	2	L1	FT	PRS
LOSS	16-30	HM	2	L1	SW	PRS
LOSS	31-45+	HM	2	L1	FT	PRS
LOSS	31-45+	HM	2	L1	SW	PRS
LOSS	46-60	HM	2	DR	FT	PRS
LOSS	46-60	HM	2	DR	FT	PRS
LOSS	46-60	HM	2	DR	SW	PRS
LOSS	46-60	HM	2	DR	SW	PRS

LOSS	46-60	HM	2	DR	SW	PRS
LOSS	61-75	HM	2	DR	SW	PRS
LOSS	61-75	HM	2	DR	SW	PRS
LOSS	76-90+	HM	2	L1	SW	PRS
LOSS	76-90+	HM	2	L2	FT	CNT
DRAW	0-15	HM	2	DR	SW	PRS
DRAW	16-30	HM	2	DR	FT	PRS
DRAW	16-30	HM	2	DR	FT	CNT
DRAW	16-30	HM	2	DR	SW	PRS
DRAW	16-30	HM	2	DR	SW	PRS
DRAW	31-45+	HM	2	DR	SW	PRS
DRAW	31-45+	HM	2	DR	FT	PRS
DRAW	31-45+	HM	2	DR	SW	PRS
DRAW	46-60	HM	2	DR	SW	PRS
DRAW	46-60	HM	2	DR	SW	PRS
DRAW	46-60	HM	2	DR	SW	PRS
DRAW	61-75	HM	2	DR	SW	PRS
DRAW	61-75	HM	2	DR	FT	PRS
DRAW	61-75	HM	2	DR	FT	PRS
DRAW	76-90+	HM	2	DR	SW	PRS
DRAW	0-15	HM	2	DR	SW	PRS
DRAW	0-15	HM	2	DR	SW	PRS
DRAW	16-30	HM	2	DR	FT	PRS
DRAW	16-30	HM	2	DR	SW	PRS
DRAW	31-45+	HM	2	DR	SW	PRS
DRAW	31-45+	HM	2	DR	FT	PRS
DRAW	46-60	HM	2	DR	SW	PRS
DRAW	46-60	HM	2	DR	SW	PRS
DRAW	61-75	HM	2	DR	SW	PRS
DRAW	76-90+	HM	2	DR	SW	PRS
DRAW	76-90+	HM	2	DR	SW	PRS
DRAW	76-90+	HM	2	DR	SW	PRS
LOSS	0-15	AW	2	DR	SW	PRS
LOSS	0-15	AW	2	DR	SW	PRS
LOSS	0-15	AW	2	DR	SW	PRS
LOSS	0-15	AW	2	DR	SW	PRS
LOSS	0-15	AW	2	DR	SW	PRS
LOSS	16-30	AW	2	DR	SW	PRS
LOSS	16-30	AW	2	DR	SW	PRS
LOSS	16-30	AW	2	DR	SW	PRS
LOSS	16-30	AW	2	DR	SW	PRS
LOSS	16-30	AW	2	DR	SW	PRS
LOSS	31-45+	AW	2	DR	SW	PRS
LOSS	31-45+	AW	2	DR	SW	PRS
LOSS	31-45+	AW	2	DR	SW	PRS
LOSS	31-45+	AW	2	DR	SW	PRS
LOSS	31-45+	AW	2	DR	SW	PRS
LOSS	46-60	AW	2	L1	SW	PRS
LOSS	46-60	AW	2	L1	SW	PRS
LOSS	46-60	AW	2	L1	SW	PRS

LOSS	46-60	AW	2 L1	SW	PRS
LOSS	46-60	AW	2 L1	SW	PRS
LOSS	61-75	AW	2 L2	SW	PRS
LOSS	61-75	AW	2 L2	SW	PRS
LOSS	61-75	AW	2 L2	SW	PRS
LOSS	61-75	AW	2 L2	SW	PRS
LOSS	76-90+	AW	2 L2	SW	PRS
LOSS	76-90+	AW	2 L2	SW	PRS
DRAW	0-15	AW	2 DR	SW	PRS
DRAW	0-15	AW	2 DR	SW	PRS
DRAW	0-15	AW	2 DR	SW	PRS
DRAW	0-15	AW	2 DR	SW	PRS
DRAW	16-30	AW	2 L1	SW	PRS
DRAW	16-30	AW	2 L1	SW	CNT
DRAW	16-30	AW	2 L1	SW	PRS
DRAW	16-30	AW	2 L1	SW	PRS
DRAW	16-30	AW	2 L1	SW	PRS
DRAW	31-45+	AW	2 DR	SW	PRS
DRAW	31-45+	AW	2 L1	SW	PRS
DRAW	31-45+	AW	2 L1	FT	PRS
DRAW	31-45+	AW	2 L1	SW	PRS
DRAW	31-45+	AW	2 L1	SW	PRS
DRAW	31-45+	AW	2 L1	FT	PRS
DRAW	31-45+	AW	2 L1	SW	PRS
DRAW	31-45+	AW	2 L1	SW	PRS
DRAW	31-45+	AW	2 L1	SW	PRS
DRAW	31-45+	AW	2 L1	SW	PRS
DRAW	46-60	AW	2 L1	FT	CNT
DRAW	46-60	AW	2 L1	SW	PRS
DRAW	46-60	AW	2 W1	SW	PRS
DRAW	61-75	AW	2 DR	SW	PRS
DRAW	76-90+	AW	2 DR	SW	PRS
DRAW	76-90+	AW	2 DR	SW	PRS
DRAW	76-90+	AW	2 DR	SW	PRS
DRAW	76-90+	AW	2 DR	FT	CNT
DRAW	76-90+	AW	2 DR	SW	PRS
WIN	0-15	HM	2 DR	SW	PRS
WIN	16-30	HM	2 DR	SW	PRS
WIN	16-30	HM	2 DR	SW	PRS
WIN	16-30	HM	2 DR	SW	PRS
WIN	31-45+	HM	2 W1	SW	PRS
WIN	31-45+	HM	2 W1	SW	PRS
WIN	46-60	HM	2 W1	SW	PRS
WIN	46-60	HM	2 W1	SW	PRS
WIN	46-60	HM	2 W1	SW	PRS
WIN	61-75	HM	2 W1	SW	PRS
WIN	61-75	HM	2 W1	SW	PRS
WIN	76-90+	HM	2 W1	SW	PRS
WIN	76-90+	HM	2 W1	SW	PRS
WIN	76-90+	HM	2 W1	SW	PRS

WIN	76-90+	HM	2	W1	SW	PRS
WIN	76-90+	HM	2	W1	SW	PRS
LOSS	0-15	HM	2	DR	SW	PRS
LOSS	0-15	HM	2	DR	SW	PRS
LOSS	0-15	HM	2	DR	FT	CNT
LOSS	0-15	HM	2	DR	SW	PRS
LOSS	0-15	HM	2	DR	SW	PRS
LOSS	16-30	HM	2	DR	SW	PRS
LOSS	16-30	HM	2	DR	SW	PRS
LOSS	16-30	HM	2	DR	SW	PRS
LOSS	31-45+	HM	2	L1	SW	PRS
LOSS	31-45+	HM	2	L1	SW	PRS
LOSS	31-45+	HM	2	L1	SW	PRS
LOSS	46-60	HM	2	L1	FT	PRS
LOSS	46-60	HM	2	L1	SW	PRS
LOSS	46-60	HM	2	L1	FT	PRS
LOSS	46-60	HM	2	L1	SW	PRS
LOSS	61-75	HM	2	L1	FT	PRS
LOSS	76-90+	HM	2	L1	SW	PRS
LOSS	76-90+	HM	2	L1	FT	PRS
LOSS	76-90+	HM	2	L1	FT	PRS
LOSS	76-90+	HM	2	L1	FT	PRS
LOSS	76-90+	HM	2	L1	SW	PRS
WIN	16-30	HM	2	W1	SW	PRS
WIN	16-30	HM	2	W1	SW	PRS
WIN	16-30	HM	2	W1	FT	PRS
WIN	16-30	HM	2	W1	SW	PRS
WIN	16-30	HM	2	W1	SW	PRS
WIN	16-30	HM	2	W1	SW	PRS
WIN	16-30	HM	2	W1	SW	PRS
WIN	31-45+	HM	2	W1	FT	PRS
WIN	31-45+	HM	2	W1	SW	PRS
WIN	31-45+	HM	2	W1	SW	PRS
WIN	31-45+	HM	2	W1	FT	PRS
WIN	46-60	HM	2	W1	SW	PRS
WIN	46-60	HM	2	W1	SW	PRS
WIN	61-75	HM	2	W1	SW	PRS
WIN	61-75	HM	2	W1	SW	PRS
WIN	76-90+	HM	2	W1	SW	PRS
WIN	76-90+	HM	2	W1	SW	PRS
WIN	76-90+	HM	2	W2	SW	PRS
WIN	76-90+	HM	2	W>2	SW	PRS
WIN	76-90+	HM	2	W>2	SW	PRS
WIN	0-15	HM	2	DR	SW	PRS
WIN	0-15	HM	2	DR	SW	PRS
WIN	0-15	HM	2	DR	SW	PRS
WIN	0-15	HM	2	DR	SW	PRS
WIN	16-30	HM	2	W2	SW	PRS
WIN	16-30	HM	2	W2	SW	PRS
WIN	31-45+	HM	2	W2	FT	PRS

WIN	46-60	HM	2	W2	SW	PRS
WIN	46-60	HM	2	W2	SW	PRS
WIN	46-60	HM	2	W2	SW	PRS
WIN	46-60	HM	2	W2	SW	PRS
WIN	46-60	HM	2	W2	SW	PRS
WIN	61-75	HM	2	W>2	SW	PRS
WIN	76-90+	HM	2	W>2	SW	PRS
WIN	76-90+	HM	2	W>2	SW	PRS
WIN	76-90+	HM	2	W>2	SW	PRS
WIN	76-90+	HM	2	W>2	SW	PRS
LOSS	0-15	HM	2	DR	FT	PRS
LOSS	16-30	HM	2	DR	SW	CNT
LOSS	16-30	HM	2	DR	SW	PRS
LOSS	31-45+	HM	2	DR	SW	PRS
LOSS	31-45+	HM	2	DR	SW	PRS
LOSS	46-60	HM	2	DR	FT	PRS
LOSS	46-60	HM	2	DR	SW	PRS
LOSS	61-75	HM	2	DR	SW	PRS
LOSS	76-90+	HM	2	DR	SW	PRS
LOSS	76-90+	HM	2	L1	SW	PRS
LOSS	76-90+	HM	2	L1	SW	PRS
LOSS	76-90+	HM	2	L1	FT	PRS
WIN	0-15	AW	1	DR	SW	PRS
WIN	0-15	AW	1	DR	SW	PRS
WIN	16-30	AW	1	DR	SW	PRS
WIN	16-30	AW	1	DR	SW	PRS
WIN	16-30	AW	1	DR	SW	CNT
WIN	31-45+	AW	1	DR	SW	CNT
WIN	31-45+	AW	1	DR	SW	CNT
WIN	46-60	AW	1	W1	SW	PRS
WIN	61-75	AW	1	W2	SW	CNT
WIN	61-75	AW	1	W2	SW	PRS
WIN	16-30	HM	1	DR	FT	PRS
WIN	31-45+	HM	1	DR	FT	CNT
WIN	61-75	HM	1	W>2	FT	PRS
WIN	76-90+	HM	1	W>2	FT	CNT
WIN	0-15	HM	1	DR	FT	CNT
WIN	0-15	HM	1	DR	FT	PRS
WIN	0-15	HM	1	DR	SW	PRS
WIN	16-30	HM	1	DR	SW	CNT
WIN	16-30	HM	1	DR	SW	PRS
WIN	16-30	HM	1	DR	FT	PRS
WIN	16-30	HM	1	DR	SW	PRS
WIN	31-45+	HM	1	DR	FT	CNT
WIN	31-45+	HM	1	DR	FT	PRS
WIN	31-45+	HM	1	DR	FT	PRS
WIN	31-45+	HM	1	DR	FT	PRS
WIN	31-45+	HM	1	DR	FT	PRS
WIN	46-60	HM	1	DR	SW	PRS
WIN	46-60	HM	1	DR	SW	PRS

WIN	61-75	HM	1	DR	FT	PRS
WIN	61-75	HM	1	DR	SW	PRS
WIN	61-75	HM	1	W1	SW	PRS
WIN	61-75	HM	1	W1	FT	PRS
WIN	76-90+	HM	1	W1	SW	PRS
WIN	76-90+	HM	1	W2	SW	PRS
WIN	76-90+	HM	1	W2	SW	PRS
WIN	76-90+	HM	1	W2	SW	PRS
WIN	76-90+	HM	1	W2	FT	PRS
WIN	0-15	HM	1	DR	SW	PRS
WIN	0-15	HM	1	DR	SW	PRS
WIN	0-15	HM	1	DR	FT	PRS
WIN	0-15	HM	1	DR	SW	PRS
WIN	0-15	HM	1	DR	SW	PRS
WIN	46-60	HM	1	W1	SW	PRS
WIN	61-75	HM	1	W2	SW	PRS
WIN	76-90+	HM	1	W2	SW	PRS
WIN	76-90+	HM	1	W2	SW	PRS
WIN	76-90+	HM	1	W2	SW	PRS
WIN	76-90+	HM	1	W2	SW	PRS
WIN	76-90+	HM	1	W2	SW	PRS
LOSS	31-45+	HM	1	L2	FT	CNT
LOSS	46-60	HM	1	L>2	FT	PRS
LOSS	76-90+	HM	1	L>2	FT	PRS
WIN	0-15	HM	1	DR	FT	PRS
WIN	0-15	HM	1	DR	FT	PRS
WIN	31-45+	HM	1	DR	SW	PRS
WIN	31-45+	HM	1	W1	FT	PRS
WIN	31-45+	HM	1	W1	SW	PRS
WIN	46-60	HM	1	W1	SW	PRS
WIN	46-60	HM	1	W1	FT	CNT
WIN	61-75	HM	1	W1	FT	PRS
WIN	76-90+	HM	1	W1	SW	PRS
DRAW	0-15	HM	1	DR	FT	CNT
DRAW	16-30	HM	1	DR	FT	CNT
DRAW	31-45+	HM	1	DR	FT	CNT
DRAW	31-45+	HM	1	DR	FT	CNT
DRAW	31-45+	HM	1	DR	SW	CNT
DRAW	31-45+	HM	1	DR	SW	CNT
DRAW	46-60	HM	1	DR	FT	PRS
DRAW	46-60	HM	1	DR	SW	PRS
DRAW	46-60	HM	1	DR	FT	PRS
DRAW	61-75	HM	1	DR	FT	CNT
DRAW	61-75	HM	1	DR	SW	PRS
DRAW	76-90+	HM	1	DR	FT	CNT
DRAW	76-90+	HM	1	DR	FT	PRS
DRAW	76-90+	HM	1	DR	FT	CNT
WIN	0-15	HM	1	DR	FT	CNT
WIN	16-30	HM	1	DR	FT	PRS
WIN	16-30	HM	1	DR	FT	PRS

WIN	16-30	HM	1	DR	FT	PRS
WIN	16-30	HM	1	DR	FT	PRS
WIN	16-30	HM	1	DR	FT	CNT
WIN	46-60	HM	1	W1	FT	CNT
WIN	46-60	HM	1	W1	SW	CNT
WIN	46-60	HM	1	W1	FT	CNT
WIN	61-75	HM	1	W1	FT	PRS
WIN	61-75	HM	1	W2	FT	PRS
WIN	76-90+	HM	1	W2	SW	CNT
WIN	76-90+	HM	1	W2	FT	PRS
WIN	76-90+	HM	1	W2	SW	PRS
WIN	76-90+	HM	1	W2	SW	PRS
WIN	0-15	HM	1	W1	SW	PRS
WIN	0-15	HM	1	W1	FT	PRS
WIN	0-15	HM	1	W1	SW	PRS
WIN	16-30	HM	1	W1	SW	PRS
WIN	31-45+	HM	1	W2	SW	PRS
WIN	31-45+	HM	1	W2	SW	PRS
WIN	31-45+	HM	1	W2	SW	PRS
WIN	31-45+	HM	1	W2	SW	PRS
WIN	31-45+	HM	1	W2	FT	CNT
WIN	31-45+	HM	1	W2	FT	PRS
WIN	46-60	HM	1	W2	SW	PRS
WIN	76-90+	HM	1	W>2	SW	PRS
WIN	76-90+	HM	1	W>2	SW	CNT
WIN	0-15	HM	1	DR	SW	PRS
WIN	0-15	HM	1	L1	FT	CNT
WIN	16-30	HM	1	L1	SW	PRS
WIN	16-30	HM	1	L1	FT	PRS
WIN	16-30	HM	1	L1	FT	CNT
WIN	31-45+	HM	1	L1	FT	PRS
WIN	31-45+	HM	1	L1	FT	PRS
WIN	31-45+	HM	1	L1	FT	PRS
WIN	61-75	HM	1	W1	SW	CNT
WIN	61-75	HM	1	W1	SW	PRS
WIN	61-75	HM	1	W1	SW	PRS
WIN	61-75	HM	1	W1	SW	PRS
WIN	76-90+	HM	1	W>2	FT	CNT
WIN	0-15	AW	1	DR	SW	PRS
WIN	0-15	AW	1	DR	SW	PRS
WIN	0-15	AW	1	DR	SW	PRS
WIN	16-30	AW	1	DR	SW	PRS
WIN	31-45+	AW	1	DR	SW	PRS
WIN	31-45+	AW	1	DR	SW	PRS
WIN	46-60	AW	1	DR	SW	PRS
WIN	46-60	AW	1	DR	SW	PRS
WIN	61-75	AW	1	DR	SW	PRS
WIN	61-75	AW	1	DR	FT	PRS
WIN	61-75	AW	1	DR	SW	PRS
WIN	61-75	AW	1	DR	SW	PRS

WIN	76-90+	AW	1	DR	SW	PRS
WIN	76-90+	AW	1	W1	SW	PRS
DRAW	0-15	AW	1	W1	SW	CNT
DRAW	16-30	AW	1	W1	SW	CNT
DRAW	16-30	AW	1	W1	SW	CNT
DRAW	31-45+	AW	1	W1	SW	PRS
DRAW	61-75	AW	1	W1	SW	PRS
DRAW	61-75	AW	1	W1	SW	PRS
DRAW	61-75	AW	1	W1	SW	PRS
DRAW	76-90+	AW	1	DR	SW	PRS
WIN	0-15	HM	1	DR	SW	PRS
WIN	16-30	HM	1	DR	SW	CNT
WIN	16-30	HM	1	DR	SW	PRS
WIN	31-45+	HM	1	DR	FT	CNT
WIN	46-60	HM	1	DR	SW	PRS
WIN	46-60	HM	1	DR	SW	PRS
WIN	46-60	HM	1	DR	SW	PRS
WIN	46-60	HM	1	DR	SW	PRS
WIN	46-60	HM	1	DR	FT	CNT
WIN	61-75	HM	1	DR	SW	PRS
WIN	61-75	HM	1	DR	SW	PRS
WIN	61-75	HM	1	DR	SW	PRS
WIN	61-75	HM	1	DR	SW	PRS
WIN	76-90+	HM	1	DR	SW	PRS
WIN	76-90+	HM	1	DR	FT	CNT
LOSS	0-15	HM	1	DR	SW	PRS
LOSS	0-15	HM	1	DR	SW	PRS
LOSS	0-15	HM	1	DR	SW	CNT
LOSS	0-15	HM	1	DR	SW	PRS
LOSS	31-45+	HM	1	DR	SW	PRS
LOSS	31-45+	HM	1	DR	SW	PRS
LOSS	46-60	HM	1	DR	SW	PRS
LOSS	46-60	HM	1	L1	SW	PRS
LOSS	61-75	HM	1	L2	FT	CNT
LOSS	76-90+	HM	1	L2	FT	PRS
LOSS	76-90+	HM	1	L2	FT	CNT
LOSS	76-90+	HM	1	L2	FT	CNT
WIN	0-15	AW	1	L1	SW	PRS
WIN	0-15	AW	1	L1	SW	PRS
WIN	0-15	AW	1	L1	FT	PRS
WIN	0-15	AW	1	L1	SW	PRS
WIN	0-15	AW	1	L1	SW	PRS
WIN	0-15	AW	1	L1	FT	PRS
WIN	16-30	AW	1	L1	SW	PRS
WIN	16-30	AW	1	L1	SW	PRS
WIN	16-30	AW	1	L1	FT	CNT
WIN	16-30	AW	1	L1	FT	PRS
WIN	16-30	AW	1	L1	SW	PRS
WIN	16-30	AW	1	L1	SW	PRS
WIN	31-45+	AW	1	DR	SW	CNT

WIN	31-45+	AW	1	DR	FT	PRS
WIN	46-60	AW	1	W1	SW	PRS
WIN	61-75	AW	1	W1	SW	PRS
WIN	76-90+	AW	1	W1	SW	PRS
WIN	76-90+	AW	1	W1	SW	PRS
WIN	76-90+	AW	1	W1	SW	PRS
LOSS	0-15	AW	1	DR	SW	CNT
LOSS	46-60	AW	1	L1	SW	PRS
LOSS	46-60	AW	1	L1	FT	PRS
LOSS	46-60	AW	1	L1	SW	PRS
LOSS	46-60	AW	1	L1	FT	PRS
WIN	0-15	AW	1	DR	SW	PRS
WIN	0-15	AW	1	DR	SW	PRS
WIN	16-30	AW	1	L1	SW	PRS
WIN	16-30	AW	1	L1	FT	CNT
WIN	16-30	AW	1	L1	SW	PRS
WIN	16-30	AW	1	L1	FT	CNT
WIN	31-45+	AW	1	L1	SW	PRS
WIN	31-45+	AW	1	L1	SW	PRS
WIN	61-75	AW	1	W1	SW	PRS
WIN	76-90+	AW	1	DR	SW	CNT
WIN	76-90+	AW	1	W1	SW	PRS
WIN	76-90+	AW	1	W1	SW	PRS
WIN	0-15	HM	1	DR	FT	PRS
WIN	0-15	HM	1	DR	SW	PRS
WIN	0-15	HM	1	DR	SW	PRS
WIN	16-30	HM	1	DR	SW	PRS
WIN	16-30	HM	1	W1	SW	PRS
WIN	16-30	HM	1	W1	SW	PRS
WIN	16-30	HM	1	W1	SW	PRS
WIN	31-45+	HM	1	W1	SW	PRS
WIN	46-60	HM	1	W1	SW	PRS
WIN	61-75	HM	1	W2	SW	PRS
WIN	61-75	HM	1	W2	SW	PRS
WIN	76-90+	HM	1	W2	SW	PRS
WIN	76-90+	HM	1	W2	SW	PRS
WIN	0-15	AW	1	DR	SW	PRS
WIN	0-15	AW	1	DR	SW	PRS
WIN	0-15	AW	1	DR	FT	CNT
WIN	0-15	AW	1	DR	SW	CNT
WIN	0-15	AW	1	DR	SW	PRS
WIN	16-30	AW	1	DR	SW	CNT
WIN	16-30	AW	1	DR	FT	CNT
WIN	16-30	AW	1	DR	SW	PRS
WIN	16-30	AW	1	DR	FT	CNT
WIN	16-30	AW	1	DR	SW	CNT
WIN	31-45+	AW	1	DR	FT	CNT
WIN	31-45+	AW	1	DR	FT	CNT
WIN	46-60	AW	1	DR	SW	PRS
WIN	61-75	AW	1	W1	SW	PRS

WIN	61-75	AW	1	W1	SW	PRS
WIN	61-75	AW	1	W1	SW	PRS
WIN	76-90+	AW	1	W1	SW	PRS
WIN	76-90+	AW	1	W1	SW	CNT
WIN	0-15	AW	1	DR	SW	PRS
WIN	0-15	AW	1	DR	SW	PRS
WIN	0-15	AW	1	DR	SW	PRS
WIN	16-30	AW	1	DR	SW	PRS
WIN	16-30	AW	1	DR	FT	CNT
WIN	31-45+	AW	1	DR	SW	PRS
WIN	31-45+	AW	1	DR	SW	CNT
WIN	31-45+	AW	1	DR	SW	CNT
WIN	31-45+	AW	1	DR	FT	PRS
WIN	46-60	AW	1	DR	SW	CNT
WIN	46-60	AW	1	DR	SW	PRS
WIN	46-60	AW	1	W1	SW	PRS
WIN	61-75	AW	1	W1	SW	PRS
WIN	61-75	AW	1	W1	SW	PRS
WIN	61-75	AW	1	W1	SW	PRS
WIN	61-75	AW	1	W1	SW	PRS
WIN	61-75	AW	1	W1	SW	PRS
WIN	61-75	AW	1	W1	SW	PRS
WIN	76-90+	AW	1	W1	SW	PRS
WIN	76-90+	AW	1	W1	SW	PRS
WIN	76-90+	AW	1	W1	SW	PRS
WIN	76-90+	AW	1	W1	SW	PRS
WIN	76-90+	AW	1	W1	SW	PRS
WIN	76-90+	AW	1	W1	SW	PRS
LOSS	0-15	AW	1	DR	SW	PRS
LOSS	0-15	AW	1	DR	SW	PRS
LOSS	31-45+	AW	1	DR	SW	CNT
LOSS	31-45+	AW	1	DR	SW	PRS
LOSS	46-60	AW	1	DR	SW	PRS
LOSS	76-90+	AW	1	L1	SW	PRS
LOSS	76-90+	AW	1	L1	SW	PRS
DRAW	0-15	AW	1	DR	SW	PRS
DRAW	0-15	AW	1	DR	SW	PRS
DRAW	0-15	AW	1	DR	FT	PRS
DRAW	0-15	AW	1	DR	SW	PRS
DRAW	16-30	AW	1	DR	SW	PRS
DRAW	16-30	AW	1	DR	FT	PRS
DRAW	16-30	AW	1	DR	SW	PRS
DRAW	31-45+	AW	1	DR	FT	PRS
DRAW	31-45+	AW	1	DR	SW	PRS
DRAW	31-45+	AW	1	DR	SW	CNT
DRAW	31-45+	AW	1	DR	SW	PRS
DRAW	31-45+	AW	1	DR	SW	PRS
DRAW	46-60	AW	1	DR	SW	PRS
DRAW	76-90+	AW	1	DR	FT	PRS
DRAW	76-90+	AW	1	DR	FT	PRS

DRAW	76-90+	AW	1 DR	SW	PRS
DRAW	76-90+	AW	1 DR	FT	CNT
DRAW	76-90+	AW	1 DR	FT	PRS
DRAW	76-90+	AW	1 DR	SW	PRS
DRAW	0-15	HM	1 L1	FT	PRS
DRAW	0-15	HM	1 L1	FT	PRS
DRAW	0-15	HM	1 L1	FT	PRS
DRAW	0-15	HM	1 L1	SW	PRS
DRAW	16-30	HM	1 L1	SW	PRS
DRAW	16-30	HM	1 L1	SW	PRS
DRAW	16-30	HM	1 L1	FT	CNT
DRAW	46-60	HM	1 L1	SW	PRS
DRAW	46-60	HM	1 L1	FT	PRS
DRAW	46-60	HM	1 L1	FT	PRS
DRAW	46-60	HM	1 L1	SW	PRS
DRAW	46-60	HM	1 L1	SW	PRS
DRAW	61-75	HM	1 L1	FT	PRS
DRAW	61-75	HM	1 L1	SW	PRS
DRAW	61-75	HM	1 L1	SW	PRS
DRAW	76-90+	HM	1 L1	SW	PRS
DRAW	76-90+	HM	1 L1	SW	PRS
DRAW	76-90+	HM	1 L1	SW	CNT
DRAW	76-90+	HM	1 L1	SW	PRS
DRAW	76-90+	HM	1 L1	FT	PRS
DRAW	76-90+	HM	1 L1	SW	PRS
DRAW	76-90+	HM	1 L1	SW	CNT
DRAW	76-90+	HM	1 DR	FT	PRS
WIN	0-15	HM	1 DR	FT	PRS
WIN	0-15	HM	1 DR	SW	PRS
WIN	0-15	HM	1 DR	SW	PRS
WIN	16-30	HM	1 DR	FT	PRS
WIN	31-45+	HM	1 W1	SW	PRS
WIN	46-60	HM	1 W1	SW	PRS
WIN	46-60	HM	1 W1	SW	PRS
WIN	46-60	HM	1 W1	SW	PRS
WIN	61-75	HM	1 W1	SW	PRS
WIN	76-90+	HM	1 W1	SW	PRS
WIN	76-90+	HM	1 W1	SW	PRS
WIN	76-90+	HM	1 W1	SW	PRS
WIN	76-90+	HM	1 W1	FT	PRS
WIN	76-90+	HM	1 W1	SW	PRS
WIN	76-90+	HM	1 W1	SW	PRS
WIN	76-90+	HM	1 W1	SW	PRS
WIN	76-90+	HM	1 W1	SW	PRS
WIN	76-90+	HM	1 W1	SW	PRS
WIN	0-15	HM	1 DR	SW	PRS
WIN	0-15	HM	1 DR	SW	PRS
WIN	0-15	HM	1 DR	SW	PRS
WIN	0-15	HM	1 DR	SW	PRS
WIN	0-15	HM	1 DR	FT	PRS

WIN	0-15	HM	1	DR	FT	PRS
WIN	0-15	HM	1	DR	FT	PRS
WIN	16-30	HM	1	DR	SW	PRS
WIN	16-30	HM	1	DR	SW	PRS
WIN	16-30	HM	1	W1	FT	PRS
WIN	31-45+	HM	1	W1	SW	PRS
WIN	31-45+	HM	1	W1	SW	PRS
WIN	31-45+	HM	1	W1	SW	PRS
WIN	46-60	HM	1	W2	SW	PRS
WIN	46-60	HM	1	W2	SW	PRS
WIN	46-60	HM	1	W2	SW	PRS
WIN	46-60	HM	1	W2	SW	PRS
WIN	46-60	HM	1	W2	SW	PRS
WIN	46-60	HM	1	W2	SW	PRS
WIN	61-75	HM	1	W2	SW	PRS
WIN	61-75	HM	1	W2	SW	PRS
WIN	76-90+	HM	1	W2	SW	PRS
WIN	76-90+	HM	1	W2	SW	PRS
WIN	76-90+	HM	1	W2	SW	PRS
WIN	76-90+	HM	1	W2	FT	PRS
WIN	0-15	HM	1	DR	SW	PRS
WIN	0-15	HM	1	DR	SW	PRS
WIN	16-30	HM	1	DR	SW	PRS
WIN	16-30	HM	1	L1	SW	PRS
WIN	16-30	HM	1	L1	SW	PRS
WIN	16-30	HM	1	L1	FT	PRS
WIN	31-45+	HM	1	DR	FT	PRS
WIN	31-45+	HM	1	DR	FT	PRS
WIN	61-75	HM	1	W1	SW	PRS
WIN	76-90+	HM	1	W1	SW	PRS
WIN	76-90+	HM	1	W1	SW	PRS
WIN	76-90+	HM	1	DR	SW	PRS
WIN	0-15	HM	1	DR	SW	PRS
WIN	0-15	HM	1	DR	FT	PRS
WIN	0-15	HM	1	DR	SW	PRS
WIN	0-15	HM	1	DR	FT	PRS
WIN	0-15	HM	1	W1	FT	PRS
WIN	0-15	HM	1	W1	SW	PRS
WIN	16-30	HM	1	W2	SW	PRS
WIN	31-45+	HM	1	W2	SW	PRS
WIN	31-45+	HM	1	W2	SW	PRS
WIN	46-60	HM	1	W2	SW	PRS
WIN	46-60	HM	1	W2	SW	PRS
WIN	46-60	HM	1	W>2	SW	PRS
WIN	46-60	HM	1	W>2	SW	PRS
WIN	46-60	HM	1	W2	SW	PRS
WIN	46-60	HM	1	W2	SW	PRS
WIN	0-15	HM	1	DR	SW	PRS
WIN	0-15	HM	1	DR	SW	PRS
WIN	0-15	HM	1	DR	SW	PRS

WIN	0-15	HM	1	DR	SW	PRS
WIN	0-15	HM	1	DR	FT	PRS
WIN	16-30	HM	1	DR	FT	PRS
WIN	16-30	HM	1	DR	SW	PRS
WIN	16-30	HM	1	DR	SW	PRS
WIN	16-30	HM	1	DR	SW	PRS
WIN	31-45+	HM	1	DR	SW	PRS
WIN	31-45+	HM	1	W1	FT	PRS
WIN	46-60	HM	1	W1	SW	PRS
WIN	46-60	HM	1	W1	SW	PRS
WIN	46-60	HM	1	W1	SW	PRS
WIN	61-75	HM	1	W2	SW	PRS
WIN	61-75	HM	1	W2	SW	PRS
WIN	61-75	HM	1	W2	SW	PRS
WIN	61-75	HM	1	W2	SW	PRS
WIN	61-75	HM	1	W2	SW	PRS
WIN	61-75	HM	1	W2	SW	PRS
WIN	61-75	HM	1	W2	SW	PRS
WIN	61-75	HM	1	W2	SW	PRS
WIN	76-90+	HM	1	W2	SW	PRS
WIN	76-90+	HM	1	W2	SW	PRS
WIN	76-90+	HM	1	W2	FT	PRS
WIN	76-90+	HM	1	W2	SW	PRS
DRAW	0-15	AW	1	DR	SW	PRS
DRAW	0-15	AW	1	DR	SW	PRS
DRAW	0-15	AW	1	DR	SW	PRS
DRAW	0-15	AW	1	DR	SW	PRS
DRAW	16-30	AW	1	DR	SW	PRS
DRAW	16-30	AW	1	DR	SW	PRS
DRAW	16-30	AW	1	DR	SW	PRS
DRAW	16-30	AW	1	DR	SW	PRS
DRAW	16-30	AW	1	DR	SW	PRS
DRAW	16-30	AW	1	DR	SW	PRS
DRAW	16-30	AW	1	DR	SW	PRS
DRAW	31-45+	AW	1	L1	SW	PRS
DRAW	31-45+	AW	1	L1	SW	PRS
DRAW	46-60	AW	1	L1	SW	PRS
DRAW	46-60	AW	1	L1	SW	PRS
DRAW	46-60	AW	1	L1	SW	CNT
DRAW	61-75	AW	1	L1	SW	PRS
DRAW	61-75	AW	1	L1	SW	PRS
DRAW	76-90+	AW	1	L1	SW	PRS
DRAW	76-90+	AW	1	L1	SW	PRS
DRAW	76-90+	AW	1	DR	SW	PRS
DRAW	76-90+	AW	1	DR	SW	PRS
DRAW	76-90+	AW	1	DR	SW	PRS
LOSS	0-15	AW	2	DR	SW	PRS
LOSS	0-15	AW	2	DR	SW	PRS
LOSS	0-15	AW	2	DR	SW	CNT
LOSS	0-15	AW	2	DR	SW	PRS

LOSS	0-15	AW	2	DR	SW	CNT
LOSS	16-30	AW	2	DR	SW	PRS
LOSS	16-30	AW	2	DR	SW	PRS
LOSS	16-30	AW	2	DR	SW	PRS
LOSS	16-30	AW	2	W1	SW	PRS
LOSS	16-30	AW	2	W1	SW	PRS
LOSS	31-45+	AW	2	W1	SW	CNT
LOSS	31-45+	AW	2	W1	SW	PRS
LOSS	31-45+	AW	2	W2	SW	PRS
LOSS	46-60	AW	2	W2	SW	PRS
LOSS	46-60	AW	2	W2	SW	PRS
LOSS	46-60	AW	2	W2	SW	PRS
LOSS	76-90+	AW	2	W1	SW	PRS
LOSS	76-90+	AW	2	W1	SW	PRS
LOSS	76-90+	AW	2	W1	SW	PRS
LOSS	76-90+	AW	2	W1	SW	PRS
LOSS	76-90+	AW	2	L1	SW	PRS
LOSS	76-90+	AW	2	L1	SW	PRS
LOSS	0-15	AW	2	W1	SW	PRS
LOSS	0-15	AW	2	W1	SW	CNT
LOSS	0-15	AW	2	W1	SW	PRS
LOSS	0-15	AW	2	DR	SW	PRS
LOSS	16-30	AW	2	DR	SW	PRS
LOSS	31-45+	AW	2	DR	SW	PRS
LOSS	31-45+	AW	2	DR	SW	PRS
LOSS	31-45+	AW	2	L1	SW	PRS
LOSS	31-45+	AW	2	L1	SW	PRS
LOSS	46-60	AW	2	L1	SW	PRS
LOSS	46-60	AW	2	L1	SW	PRS
LOSS	46-60	AW	2	L1	FT	PRS
LOSS	46-60	AW	2	L1	SW	PRS
LOSS	61-75	AW	2	L1	SW	CNT
LOSS	61-75	AW	2	L1	SW	PRS
LOSS	61-75	AW	2	L1	SW	PRS
LOSS	0-15	HM	2	DR	SW	PRS
LOSS	16-30	HM	2	DR	SW	PRS
LOSS	16-30	HM	2	DR	SW	PRS
LOSS	16-30	HM	2	L1	FT	CNT
LOSS	31-45+	HM	2	L2	SW	PRS
LOSS	31-45+	HM	2	L>2	FT	CNT
LOSS	31-45+	HM	2	L>2	SW	PRS
LOSS	31-45+	HM	2	L>2	FT	PRS
LOSS	31-45+	HM	2	L>2	SW	PRS
LOSS	31-45+	HM	2	L>2	SW	PRS
LOSS	46-60	HM	2	L>2	SW	PRS
LOSS	46-60	HM	2	L2	SW	PRS
LOSS	46-60	HM	2	L2	FT	PRS
LOSS	46-60	HM	2	L2	SW	PRS
LOSS	46-60	HM	2	L2	SW	CNT
LOSS	46-60	HM	2	L2	FT	PRS

LOSS	76-90+	HM	2	L>2	SW	PRS
LOSS	76-90+	HM	2	L>2	SW	PRS
LOSS	76-90+	HM	2	L>2	FT	CNT
DRAW	0-15	HM	2	DR	SW	PRS
DRAW	0-15	HM	2	DR	SW	PRS
DRAW	0-15	HM	2	DR	SW	CNT
DRAW	0-15	HM	2	DR	SW	PRS
DRAW	0-15	HM	2	DR	SW	PRS
DRAW	16-30	HM	2	W1	SW	PRS
DRAW	31-45+	HM	2	W1	SW	PRS
DRAW	31-45+	HM	2	W1	SW	PRS
DRAW	31-45+	HM	2	W1	SW	PRS
DRAW	31-45+	HM	2	W1	SW	PRS
DRAW	31-45+	HM	2	W1	SW	PRS
DRAW	31-45+	HM	2	W1	SW	PRS
DRAW	46-60	HM	2	W1	SW	PRS
DRAW	46-60	HM	2	W1	SW	PRS
DRAW	46-60	HM	2	W1	SW	PRS
DRAW	61-75	HM	2	DR	FT	PRS
DRAW	76-90+	HM	2	DR	SW	CNT
DRAW	76-90+	HM	2	DR	FT	CNT
DRAW	76-90+	HM	2	DR	SW	PRS
DRAW	76-90+	HM	2	DR	SW	PRS
LOSS	0-15	AW	2	DR	SW	PRS
LOSS	16-30	AW	2	L1	SW	PRS
LOSS	16-30	AW	2	L1	SW	PRS
LOSS	16-30	AW	2	L1	SW	CNT
LOSS	16-30	AW	2	L1	SW	CNT
LOSS	16-30	AW	2	L1	SW	CNT
LOSS	16-30	AW	2	L1	SW	PRS
LOSS	16-30	AW	2	L1	SW	PRS
LOSS	31-45+	AW	2	L1	SW	PRS
LOSS	31-45+	AW	2	L1	SW	PRS
LOSS	31-45+	AW	2	L1	SW	CNT
LOSS	46-60	AW	2	L1	SW	PRS
LOSS	46-60	AW	2	L1	SW	PRS
LOSS	46-60	AW	2	L1	SW	PRS
LOSS	61-75	AW	2	L2	SW	PRS
LOSS	61-75	AW	2	L2	SW	CNT
LOSS	61-75	AW	2	L>2	SW	PRS
LOSS	76-90+	AW	2	L>2	SW	PRS
LOSS	76-90+	AW	2	L>2	FT	PRS
LOSS	76-90+	AW	2	L>2	SW	PRS
LOSS	76-90+	AW	2	L>2	SW	PRS
LOSS	0-15	AW	2	DR	SW	CNT
LOSS	16-30	AW	2	L2	SW	PRS
LOSS	16-30	AW	2	L2	SW	CNT
LOSS	31-45+	AW	2	L2	SW	PRS
LOSS	46-60	AW	2	L2	SW	PRS
LOSS	46-60	AW	2	L2	SW	PRS

LOSS	46-60	AW	2	L2	SW	PRS
LOSS	61-75	AW	2	L2	SW	CNT
LOSS	76-90+	AW	2	L2	FT	CNT
LOSS	76-90+	AW	2	L2	SW	PRS
WIN	0-15	HM	2	DR	SW	PRS
WIN	0-15	HM	2	DR	SW	PRS
WIN	0-15	HM	2	DR	SW	PRS
WIN	0-15	HM	2	DR	FT	PRS
WIN	0-15	HM	2	DR	FT	PRS
WIN	0-15	HM	2	DR	SW	PRS
WIN	16-30	HM	2	DR	SW	PRS
WIN	16-30	HM	2	DR	SW	PRS
WIN	16-30	HM	2	DR	SW	PRS
WIN	16-30	HM	2	DR	SW	PRS
WIN	16-30	HM	2	DR	FT	PRS
WIN	31-45+	HM	2	W1	SW	PRS
WIN	46-60	HM	2	W1	SW	PRS
WIN	46-60	HM	2	W1	SW	PRS
WIN	46-60	HM	2	W1	SW	PRS
WIN	61-75	HM	2	W2	SW	PRS
DRAW	0-15	AW	1	DR	SW	PRS
DRAW	0-15	AW	1	DR	SW	CNT
DRAW	0-15	AW	1	DR	SW	PRS
DRAW	16-30	AW	1	DR	SW	PRS
DRAW	16-30	AW	1	DR	SW	PRS
DRAW	16-30	AW	1	DR	SW	PRS
DRAW	16-30	AW	1	DR	SW	PRS
DRAW	16-30	AW	1	DR	SW	PRS
DRAW	16-30	AW	1	DR	FT	PRS
DRAW	16-30	AW	1	DR	SW	PRS
DRAW	16-30	AW	1	DR	SW	PRS
DRAW	16-30	AW	1	DR	SW	PRS
DRAW	16-30	AW	1	W1	SW	PRS
DRAW	31-45+	AW	1	W1	SW	PRS
DRAW	31-45+	AW	1	W1	SW	PRS
DRAW	31-45+	AW	1	W1	SW	PRS
DRAW	31-45+	AW	1	W1	SW	PRS
DRAW	31-45+	AW	1	W1	SW	PRS
DRAW	46-60	AW	1	W1	SW	PRS
DRAW	46-60	AW	1	W1	SW	PRS
DRAW	46-60	AW	1	W1	SW	PRS
DRAW	46-60	AW	1	W1	SW	PRS
DRAW	61-75	AW	1	W1	SW	PRS
DRAW	76-90+	AW	1	DR	FT	CNT
DRAW	76-90+	AW	1	DR	SW	PRS
DRAW	76-90+	AW	1	DR	SW	PRS
DRAW	76-90+	AW	1	DR	SW	PRS
DRAW	76-90+	AW	1	DR	FT	PRS
DRAW	76-90+	AW	1	DR	SW	PRS
WIN	0-15	AW	1	DR	SW	PRS

WIN	0-15	AW	1	DR	SW	PRS
WIN	16-30	AW	1	DR	SW	PRS
WIN	16-30	AW	1	DR	SW	PRS
WIN	16-30	AW	1	DR	SW	PRS
WIN	31-45+	AW	1	DR	SW	PRS
WIN	31-45+	AW	1	DR	SW	PRS
WIN	31-45+	AW	1	DR	SW	PRS
WIN	46-60	AW	1	W1	SW	PRS
WIN	46-60	AW	1	W1	SW	PRS
WIN	46-60	AW	1	W1	SW	PRS
WIN	46-60	AW	1	W1	SW	PRS
WIN	61-75	AW	1	W1	SW	PRS
WIN	76-90+	AW	1	W1	SW	PRS
WIN	76-90+	AW	1	W1	SW	PRS
WIN	76-90+	AW	1	W1	SW	PRS
WIN	76-90+	AW	1	W2	SW	PRS
WIN	76-90+	AW	1	W2	SW	PRS
WIN	76-90+	AW	1	W2	SW	PRS
WIN	0-15	HM	1	DR	SW	PRS
WIN	0-15	HM	1	DR	SW	PRS
WIN	0-15	HM	1	DR	SW	PRS
WIN	0-15	HM	1	DR	SW	PRS
WIN	0-15	HM	1	DR	FT	PRS
WIN	16-30	HM	1	W2	SW	PRS
WIN	31-45+	HM	1	W2	SW	PRS
WIN	31-45+	HM	1	W2	FT	CNT
WIN	31-45+	HM	1	W2	FT	PRS
WIN	46-60	HM	1	W2	SW	PRS
WIN	61-75	HM	1	W2	SW	PRS
WIN	76-90+	HM	1	W2	SW	PRS
WIN	76-90+	HM	1	W2	SW	PRS
WIN	76-90+	HM	1	W2	SW	PRS
LOSS	16-30	HM	1	DR	SW	PRS
LOSS	16-30	HM	1	DR	FT	CNT
LOSS	31-45+	HM	1	DR	SW	PRS
LOSS	31-45+	HM	1	DR	SW	PRS
LOSS	31-45+	HM	1	DR	FT	PRS
LOSS	46-60	HM	1	L1	SW	PRS
LOSS	46-60	HM	1	L1	SW	PRS
LOSS	46-60	HM	1	L1	SW	PRS
LOSS	61-75	HM	1	L1	SW	PRS
LOSS	76-90+	HM	1	DR	SW	PRS
LOSS	76-90+	HM	1	DR	FT	PRS
LOSS	76-90+	HM	1	DR	SW	PRS
LOSS	76-90+	HM	1	DR	SW	PRS
LOSS	76-90+	HM	1	L1	SW	PRS
LOSS	76-90+	HM	1	L2	SW	PRS
WIN	0-15	AW	1	DR	SW	PRS
WIN	16-30	AW	1	DR	SW	PRS
WIN	16-30	AW	1	DR	SW	PRS

WIN	16-30	AW	1	DR	SW	PRS
WIN	31-45+	AW	1	W1	SW	PRS
WIN	31-45+	AW	1	W2	SW	PRS
WIN	31-45+	AW	1	W2	SW	PRS
WIN	46-60	AW	1	W2	SW	PRS
WIN	46-60	AW	1	W2	SW	PRS
WIN	61-75	AW	1	W1	SW	PRS
WIN	76-90+	AW	1	W2	SW	PRS

YES	ST	FW	RT	OF	LOST POSSESSION
YES	ME	FW	RT	DF	UNSUCCESFULL
YES	ST	BW	RT	OF	CONTINUED POSSESSION
YES	ME	FW	LT	MO	CONTINUED POSSESSION
YES	ST	FW	LT	OF	LOST POSSESSION
YES	ME	FW	LT	CE	LOST POSSESSION
NO	ST	BW	LT	MO	CONTINUED POSSESSION
YES	ME	FW	RT	MO	LOST POSSESSION
YES	ME	FW	LT	MO	CONTINUED POSSESSION
YES	ME	FW	LT	MD	LOST POSSESSION
YES	ST	FW	RT	CE	LOST POSSESSION
YES	ME	BW	RT	CE	CONTINUED POSSESSION
YES	ST	FW	LT	MO	LOST POSSESSION
YES	ST	BW	LT	CE	LOST POSSESSION
YES	ST	FW	LT	MO	LOST POSSESSION
YES	ME	FW	RT	CE	LOST POSSESSION
YES	ME	FW	LT	MO	LOST POSSESSION
YES	ST	FW	LT	MD	CONTINUED POSSESSION
YES	ME	FW	LT	DF	LOST POSSESSION
NO	ST	BW	LT	MO	CONTINUED POSSESSION
YES	ME	FW	LT	MO	LOST POSSESSION
NO	ME	BW	LT	CE	CONTINUED POSSESSION
YES	ME	FW	LT	OF	LOST POSSESSION
YES	ME	FW	RT	MD	LOST POSSESSION
YES	ME	FW	RT	MD	LOST POSSESSION
YES	ST	FW	LT	OF	LOST POSSESSION
YES	ST	FW	RT	MD	LOST POSSESSION
YES	ST	FW	LT	MO	LOST POSSESSION
YES	ME	FW	LT	MO	LOST POSSESSION
YES	ME	BW	RT	CE	CONTINUED POSSESSION
YES	ME	FW	RT	MD	CONTINUED POSSESSION
YES	ME	FW	RT	MO	CONTINUED POSSESSION
YES	ME	FW	RT	MO	CONTINUED POSSESSION
YES	ME	FW	RT	OF	SUCCESFULL
NO	ME	BW	RT	CE	CONTINUED POSSESSION
YES	ME	FW	RT	MD	LOST POSSESSION
YES	ME	BW	LT	CE	CONTINUED POSSESSION
YES	ST	FW	LT	MO	SUCCESFULL
YES	ST	BW	RT	CE	CONTINUED POSSESSION
YES	ST	FW	RT	MO	CONTINUED POSSESSION
YES	ME	BW	RT	MO	CONTINUED POSSESSION
YES	ST	BW	RT	MO	CONTINUED POSSESSION
YES	ME	FW	RT	MD	LOST POSSESSION
YES	ME	FW	RT	CE	CONTINUED POSSESSION
YES	ME	FW	RT	MO	CONTINUED POSSESSION
YES	ME	FW	LT	MD	LOST POSSESSION
YES	ST	FW	LT	CE	SUCCESFULL
NO	ME	BW	LT	MD	CONTINUED POSSESSION
YES	LG	FW	RT	DF	LOST POSSESSION
YES	ST	FW	RT	OF	LOST POSSESSION

YES	ST	FW	RT	MO	LOST POSSESSION
YES	ST	FW	RT	CE	LOST POSSESSION
YES	ME	FW	RT	MD	CONTINUED POSSESSION
YES	ME	FW	RT	MD	CONTINUED POSSESSION
YES	ST	FW	LT	DF	LOST POSSESSION
YES	ST	FW	RT	CE	LOST POSSESSION
YES	ST	FW	RT	CE	CONTINUED POSSESSION
YES	ST	FW	RT	MO	LOST POSSESSION
YES	ME	FW	RT	MO	SUCCESFULL
NO	ME	FW	RT	OF	LOST POSSESSION
YES	ME	FW	LT	CE	LOST POSSESSION
YES	ST	FW	RT	MO	LOST POSSESSION
YES	ME	FW	RT	MO	LOST POSSESSION
NO	ME	FW	RT	MD	CONTINUED POSSESSION
YES	ST	FW	RT	MO	LOST POSSESSION
YES	ST	FW	RT	OF	LOST POSSESSION
NO	ME	FW	RT	MD	LOST POSSESSION
NO	ME	FW	LT	CE	UNSUCCESFULL
YES	ME	FW	LT	CE	UNSUCCESFULL
YES	ST	BW	LT	MO	CONTINUED POSSESSION
YES	ST	FW	LT	MO	LOST POSSESSION
YES	ST	BW	RT	OF	LOST POSSESSION
YES	ST	FW	RT	CE	CONTINUED POSSESSION
YES	ST	BW	RT	OF	CONTINUED POSSESSION
YES	ST	FW	RT	OF	LOST POSSESSION
YES	ME	FW	RT	MD	CONTINUED POSSESSION
YES	ME	BW	LT	MO	LOST POSSESSION
YES	ST	BW	RT	MO	CONTINUED POSSESSION
YES	ST	FW	LT	CE	CONTINUED POSSESSION
YES	ST	FW	LT	MO	CONTINUED POSSESSION
YES	ME	FW	RT	MD	CONTINUED POSSESSION
YES	ST	FW	RT	OF	SUCCESFULL
YES	ST	FW	LT	MO	LOST POSSESSION
YES	ST	FW	RT	MD	LOST POSSESSION
YES	ME	BW	LT	MD	LOST POSSESSION
YES	ME	BW	LT	OF	LOST POSSESSION
YES	ST	FW	RT	CE	LOST POSSESSION
YES	ME	FW	LT	DF	LOST POSSESSION
YES	ST	FW	LT	CE	CONTINUED POSSESSION
YES	ST	BW	LT	MO	SUCCESFULL
NO	ST	BW	LT	CE	LOST POSSESSION
YES	ME	FW	RT	MD	LOST POSSESSION
YES	ST	BW	LT	CE	CONTINUED POSSESSION
YES	ME	BW	RT	MO	CONTINUED POSSESSION
YES	ST	FW	LT	MO	LOST POSSESSION
YES	ST	FW	LT	CE	CONTINUED POSSESSION
YES	ST	FW	LT	CE	CONTINUED POSSESSION
YES	ME	FW	RT	MD	CONTINUED POSSESSION
YES	ST	FW	RT	MD	CONTINUED POSSESSION
YES	ME	FW	RT	MD	LOST POSSESSION

YES	ME	FW	RT	MD	LOST POSSESSION
YES	ST	FW	LT	MD	LOST POSSESSION
YES	ME	FW	RT	MO	CONTINUED POSSESSION
YES	ME	FW	RT	MO	LOST POSSESSION
YES	ST	FW	LT	OF	CONTINUED POSSESSION
YES	ST	FW	RT	CE	LOST POSSESSION
YES	ST	FW	RT	MO	LOST POSSESSION
YES	ST	FW	LT	CE	LOST POSSESSION
YES	ME	FW	RT	MD	LOST POSSESSION
YES	ST	FW	LT	OF	SUCCESFULL
YES	ST	FW	RT	CE	LOST POSSESSION
YES	ME	FW	RT	MD	LOST POSSESSION
YES	ME	FW	LT	MD	CONTINUED POSSESSION
YES	ME	FW	LT	DF	LOST POSSESSION
YES	ME	BW	LT	OF	CONTINUED POSSESSION
YES	LG	FW	RT	MD	LOST POSSESSION
YES	ME	FW	LT	DF	LOST POSSESSION
YES	ME	FW	RT	MD	LOST POSSESSION
YES	ME	FW	RT	MD	LOST POSSESSION
YES	ME	FW	LT	OF	LOST POSSESSION
YES	LG	FW	LT	OF	LOST POSSESSION
YES	ME	FW	RT	MD	CONTINUED POSSESSION
YES	ME	FW	RT	MD	CONTINUED POSSESSION
YES	ME	FW	LT	MD	LOST POSSESSION
YES	ME	BW	RT	CE	CONTINUED POSSESSION
YES	ME	FW	LT	MO	CONTINUED POSSESSION
YES	ME	BW	RT	MO	CONTINUED POSSESSION
YES	ME	BW	RT	MO	CONTINUED POSSESSION
YES	ME	BW	RT	MD	CONTINUED POSSESSION
YES	ME	FW	RT	MD	CONTINUED POSSESSION
YES	ME	FW	LT	MD	LOST POSSESSION
YES	LG	FW	LT	MD	LOST POSSESSION
YES	ME	BW	RT	CE	LOST POSSESSION
YES	ME	FW	LT	CE	CONTINUED POSSESSION
YES	ST	FW	RT	MO	LOST POSSESSION
YES	ME	FW	RT	OF	CONTINUED POSSESSION
YES	ST	BW	RT	OF	LOST POSSESSION
YES	ST	FW	LT	CE	CONTINUED POSSESSION
YES	LG	FW	RT	MD	LOST POSSESSION
YES	ME	FW	RT	CE	CONTINUED POSSESSION
YES	ST	FW	RT	CE	LOST POSSESSION
YES	ST	FW	RT	OF	LOST POSSESSION
YES	ST	FW	LT	MD	CONTINUED POSSESSION
YES	ME	FW	RT	MO	CONTINUED POSSESSION
YES	ST	FW	LT	OF	LOST POSSESSION
YES	ST	FW	LT	MD	CONTINUED POSSESSION
YES	ST	FW	RT	MD	CONTINUED POSSESSION
YES	ME	FW	RT	MO	CONTINUED POSSESSION
YES	ME	FW	LT	MD	LOST POSSESSION
YES	ST	BW	RT	CE	CONTINUED POSSESSION

YES	ST	BW	RT	CE	LOST POSSESSION
YES	ME	FW	LT	MO	LOST POSSESSION
YES	ME	FW	RT	MO	CONTINUED POSSESSION
NO	ME	BW	LT	CE	CONTINUED POSSESSION
YES	ST	FW	RT	DF	LOST POSSESSION
YES	ME	FW	RT	MD	LOST POSSESSION
YES	ME	FW	LT	MO	CONTINUED POSSESSION
YES	ME	FW	RT	DF	CONTINUED POSSESSION
YES	ST	FW	LT	CE	LOST POSSESSION
YES	ME	FW	LT	DF	LOST POSSESSION
YES	ST	FW	LT	MO	LOST POSSESSION
NO	ST	BW	RT	MO	CONTINUED POSSESSION
YES	ST	FW	LT	MD	CONTINUED POSSESSION
YES	ST	FW	LT	OF	LOST POSSESSION
YES	ME	FW	RT	MO	LOST POSSESSION
YES	ST	BW	RT	MO	SUCCESFULL
YES	ME	BW	LT	CE	SUCCESFULL
YES	ST	BW	LT	CE	CONTINUED POSSESSION
YES	ST	BW	RT	OF	CONTINUED POSSESSION
YES	ST	BW	LT	MO	CONTINUED POSSESSION
NO	ME	BW	RT	CE	CONTINUED POSSESSION
YES	ST	FW	LT	MO	CONTINUED POSSESSION
YES	ST	FW	LT	DF	UNSUCCESFULL
YES	ST	BW	LT	MO	LOST POSSESSION
YES	ME	FW	LT	CE	LOST POSSESSION
YES	ME	FW	RT	MO	CONTINUED POSSESSION
YES	ME	FW	RT	OF	LOST POSSESSION
YES	ST	FW	LT	MD	LOST POSSESSION
YES	ST	FW	LT	MO	CONTINUED POSSESSION
YES	ME	BW	RT	OF	LOST POSSESSION
YES	ST	FW	LT	MO	LOST POSSESSION
YES	ST	FW	RT	CE	LOST POSSESSION
YES	ST	BW	LT	MO	CONTINUED POSSESSION
YES	ST	BW	RT	OF	CONTINUED POSSESSION
YES	ST	FW	RT	MD	CONTINUED POSSESSION
YES	ST	FW	RT	CE	CONTINUED POSSESSION
YES	ST	FW	RT	OF	LOST POSSESSION
YES	ST	FW	LT	MO	CONTINUED POSSESSION
YES	ST	BW	RT	OF	LOST POSSESSION
YES	LG	BW	LT	OF	CONTINUED POSSESSION
YES	ST	FW	RT	MO	CONTINUED POSSESSION
YES	ME	FW	RT	DF	LOST POSSESSION
YES	ME	FW	RT	MO	CONTINUED POSSESSION
YES	LG	FW	RT	OF	CONTINUED POSSESSION
YES	ST	FW	LT	CE	CONTINUED POSSESSION
YES	ST	FW	RT	OF	CONTINUED POSSESSION
YES	ME	FW	LT	MO	LOST POSSESSION
YES	ST	FW	RT	CE	CONTINUED POSSESSION
YES	ST	BW	LT	OF	CONTINUED POSSESSION
YES	ST	BW	RT	CE	CONTINUED POSSESSION

YES	ME	FW	RT	MO	CONTINUED POSSESSION
YES	ST	BW	RT	OF	LOST POSSESSION
YES	ME	BW	RT	CE	CONTINUED POSSESSION
YES	ST	FW	LT	MO	CONTINUED POSSESSION
YES	ME	BW	LT	MO	CONTINUED POSSESSION
YES	ME	BW	RT	MO	LOST POSSESSION
YES	ME	FW	RT	MO	SUCCESFULL
NO	ME	BW	RT	MD	CONTINUED POSSESSION
YES	ST	BW	RT	MO	CONTINUED POSSESSION
YES	ST	BW	RT	MO	CONTINUED POSSESSION
YES	ST	BW	LT	MO	CONTINUED POSSESSION
YES	ME	FW	RT	DF	CONTINUED POSSESSION
YES	ST	FW	LT	MO	LOST POSSESSION
YES	ME	BW	RT	OF	CONTINUED POSSESSION
YES	ST	FW	LT	CE	CONTINUED POSSESSION
YES	ST	BW	LT	OF	CONTINUED POSSESSION
YES	ST	FW	RT	MO	CONTINUED POSSESSION
YES	ST	BW	RT	CE	CONTINUED POSSESSION
YES	ME	BW	RT	MD	CONTINUED POSSESSION
YES	ME	BW	LT	MO	LOST POSSESSION
YES	ME	BW	RT	CE	CONTINUED POSSESSION
YES	ST	FW	RT	MO	LOST POSSESSION
YES	ST	BW	LT	MO	CONTINUED POSSESSION
YES	ME	FW	LT	MO	CONTINUED POSSESSION
YES	ST	FW	LT	OF	LOST POSSESSION
YES	ST	FW	RT	DF	LOST POSSESSION
YES	ST	BW	RT	OF	LOST POSSESSION
YES	ST	BW	LT	MO	CONTINUED POSSESSION
YES	ST	FW	RT	MD	CONTINUED POSSESSION
YES	ST	FW	LT	OF	CONTINUED POSSESSION
YES	ST	BW	LT	CE	CONTINUED POSSESSION
YES	ME	FW	RT	OF	LOST POSSESSION
YES	ST	BW	LT	MO	CONTINUED POSSESSION
YES	ME	FW	LT	MD	LOST POSSESSION
YES	ST	FW	RT	DF	CONTINUED POSSESSION
YES	ST	BW	RT	MD	LOST POSSESSION
YES	ME	BW	LT	OF	CONTINUED POSSESSION
YES	ST	FW	LT	MO	CONTINUED POSSESSION
YES	ST	BW	LT	OF	CONTINUED POSSESSION
YES	ME	FW	LT	OF	CONTINUED POSSESSION
YES	ME	FW	LT	MO	CONTINUED POSSESSION
YES	ST	BW	RT	OF	CONTINUED POSSESSION
YES	ME	BW	LT	OF	SUCCESFULL
YES	ME	BW	LT	MD	CONTINUED POSSESSION
YES	ST	BW	RT	MO	CONTINUED POSSESSION
YES	ST	BW	LT	MO	CONTINUED POSSESSION
YES	ST	BW	LT	OF	LOST POSSESSION
YES	ME	BW	LT	MO	CONTINUED POSSESSION
YES	ST	FW	RT	MO	SUCCESFULL
YES	ST	FW	LT	MD	CONTINUED POSSESSION

YES	ST	BW	LT	MO	CONTINUED POSSESSION
YES	LG	FW	RT	OF	CONTINUED POSSESSION
YES	ST	FW	LT	MO	SUCCESFULL
YES	ME	FW	LT	CE	CONTINUED POSSESSION
YES	LG	FW	RT	OF	SUCCESFULL
YES	ME	BW	RT	CE	CONTINUED POSSESSION
YES	ME	BW	RT	CE	LOST POSSESSION
YES	ST	FW	RT	MO	LOST POSSESSION
YES	ME	BW	RT	MD	CONTINUED POSSESSION
YES	ST	FW	LT	MO	LOST POSSESSION
YES	ME	BW	RT	CE	CONTINUED POSSESSION
YES	ME	FW	LT	MO	LOST POSSESSION
YES	ST	FW	LT	MD	CONTINUED POSSESSION
YES	ST	FW	LT	MD	SUCCESFULL
YES	ME	BW	LT	OF	CONTINUED POSSESSION
YES	ST	FW	LT	MO	LOST POSSESSION
YES	ST	BW	LT	MD	CONTINUED POSSESSION
YES	ST	FW	LT	MD	CONTINUED POSSESSION
YES	ST	BW	RT	MO	CONTINUED POSSESSION
YES	ST	FW	LT	OF	CONTINUED POSSESSION
YES	ST	BW	RT	OF	CONTINUED POSSESSION
YES	ST	FW	LT	MD	LOST POSSESSION
NO	ST	BW	RT	OF	CONTINUED POSSESSION
YES	ME	BW	LT	MO	CONTINUED POSSESSION
YES	ST	BW	LT	MO	CONTINUED POSSESSION
YES	ST	BW	LT	MO	CONTINUED POSSESSION
YES	ST	FW	LT	OF	CONTINUED POSSESSION
YES	ST	FW	RT	OF	LOST POSSESSION
YES	ST	BW	LT	MO	CONTINUED POSSESSION
YES	ST	FW	RT	CE	SUCCESFULL
YES	ST	FW	RT	MO	LOST POSSESSION
YES	ST	FW	RT	CE	CONTINUED POSSESSION
YES	ME	BW	LT	MO	CONTINUED POSSESSION
YES	ST	FW	LT	MO	LOST POSSESSION
YES	ST	FW	LT	CE	CONTINUED POSSESSION
YES	ST	BW	RT	MO	CONTINUED POSSESSION
YES	LG	BW	RT	OF	CONTINUED POSSESSION
YES	ST	FW	RT	MD	LOST POSSESSION
YES	ME	FW	LT	MO	LOST POSSESSION
YES	ME	BW	RT	MO	SUCCESFULL
YES	ST	FW	LT	CE	CONTINUED POSSESSION
YES	ST	FW	LT	CE	CONTINUED POSSESSION
YES	ST	BW	LT	MO	SUCCESFULL
YES	ME	FW	RT	CE	LOST POSSESSION
YES	ST	FW	LT	MO	LOST POSSESSION
YES	ME	FW	LT	MD	LOST POSSESSION
YES	ST	FW	RT	MD	LOST POSSESSION
YES	ST	FW	LT	MD	LOST POSSESSION
NO	LG	FW	RT	MD	LOST POSSESSION
YES	ME	FW	LT	MO	CONTINUED POSSESSION

NO	ME	FW	RT	MD	LOST POSSESSION
YES	ST	BW	RT	MD	SUCCESFULL
YES	ME	FW	LT	CE	LOST POSSESSION
NO	ME	FW	RT	MD	LOST POSSESSION
YES	ST	FW	LT	CE	CONTINUED POSSESSION
YES	ST	FW	RT	DF	CONTINUED POSSESSION
YES	ME	FW	RT	MD	UNSUCCESFULL
YES	ME	FW	RT	MD	CONTINUED POSSESSION
YES	ST	FW	LT	OF	LOST POSSESSION
YES	LG	FW	RT	MD	CONTINUED POSSESSION
YES	ME	FW	RT	CE	CONTINUED POSSESSION
YES	ME	BW	RT	OF	CONTINUED POSSESSION
YES	ST	BW	RT	MO	CONTINUED POSSESSION
YES	ST	FW	RT	MD	LOST POSSESSION
YES	ST	BW	RT	MO	CONTINUED POSSESSION
YES	ST	FW	LT	CE	LOST POSSESSION
YES	ME	FW	RT	MO	LOST POSSESSION
YES	ST	FW	LT	MO	CONTINUED POSSESSION
YES	ST	FW	LT	MD	CONTINUED POSSESSION
YES	ST	FW	LT	CE	LOST POSSESSION
YES	ST	FW	RT	MO	CONTINUED POSSESSION
YES	ST	FW	RT	MO	LOST POSSESSION
YES	ST	FW	LT	MD	LOST POSSESSION
YES	ME	FW	LT	CE	LOST POSSESSION
YES	ST	FW	RT	CE	LOST POSSESSION
YES	ME	FW	LT	MD	LOST POSSESSION
YES	ME	FW	LT	MD	CONTINUED POSSESSION
YES	ST	FW	LT	OF	LOST POSSESSION
YES	ST	FW	LT	CE	LOST POSSESSION
YES	ST	FW	RT	MD	LOST POSSESSION
YES	ME	FW	LT	MD	LOST POSSESSION
YES	ST	FW	LT	DF	SUCCESFULL
YES	ME	FW	RT	CE	CONTINUED POSSESSION
YES	ME	FW	RT	DF	CONTINUED POSSESSION
YES	ST	BW	RT	MO	CONTINUED POSSESSION
YES	ME	FW	RT	MD	CONTINUED POSSESSION
YES	ME	FW	RT	MO	CONTINUED POSSESSION
NO	ST	BW	RT	MO	CONTINUED POSSESSION
NO	ME	BW	RT	CE	CONTINUED POSSESSION
NO	ST	BW	LT	CE	CONTINUED POSSESSION
NO	LG	BW	LT	MO	CONTINUED POSSESSION
NO	LG	BW	LT	MO	CONTINUED POSSESSION
YES	ME	BW	LT	MO	CONTINUED POSSESSION
YES	ST	FW	RT	MO	CONTINUED POSSESSION
YES	ST	FW	RT	MO	CONTINUED POSSESSION
YES	ST	FW	RT	CE	CONTINUED POSSESSION
YES	ST	BW	LT	MO	SUCCESFULL
YES	ST	BW	LT	MO	LOST POSSESSION
NO	ST	BW	RT	MD	CONTINUED POSSESSION
YES	ST	FW	RT	MO	LOST POSSESSION

NO	ME	BW	RT	MO	CONTINUED POSSESSION
NO	ME	BW	LT	CE	LOST POSSESSION
YES	ST	BW	LT	MO	CONTINUED POSSESSION
YES	ST	BW	RT	OF	LOST POSSESSION
YES	ST	BW	RT	MO	CONTINUED POSSESSION
YES	ST	FW	RT	OF	CONTINUED POSSESSION
YES	ST	FW	LT	MO	CONTINUED POSSESSION
YES	ST	BW	RT	MO	CONTINUED POSSESSION
YES	ST	FW	LT	OF	LOST POSSESSION
YES	LG	BW	RT	MO	CONTINUED POSSESSION
YES	ME	FW	RT	MD	CONTINUED POSSESSION
YES	ST	FW	RT	CE	CONTINUED POSSESSION
YES	ST	FW	RT	MO	CONTINUED POSSESSION
YES	ME	FW	RT	CE	CONTINUED POSSESSION
YES	ME	FW	RT	CE	CONTINUED POSSESSION
YES	ST	BW	RT	MO	LOST POSSESSION
YES	ME	FW	RT	CE	CONTINUED POSSESSION
YES	ST	FW	RT	MO	CONTINUED POSSESSION
YES	ME	FW	RT	MD	CONTINUED POSSESSION
YES	ME	BW	RT	MO	SUCCESFULL
YES	ME	FW	LT	OF	CONTINUED POSSESSION
YES	ST	FW	RT	CE	LOST POSSESSION
YES	ME	FW	LT	OF	SUCCESFULL
YES	ME	BW	LT	MD	LOST POSSESSION
YES	ME	FW	RT	MO	LOST POSSESSION
YES	ST	FW	LT	CE	LOST POSSESSION
YES	ME	FW	RT	OF	CONTINUED POSSESSION
YES	ST	BW	LT	DF	LOST POSSESSION
YES	ME	FW	RT	MD	LOST POSSESSION
YES	ME	FW	RT	CE	LOST POSSESSION
YES	ST	FW	RT	MO	CONTINUED POSSESSION
YES	LG	BW	RT	OF	LOST POSSESSION
YES	ME	FW	LT	MD	LOST POSSESSION
YES	ST	BW	RT	DF	LOST POSSESSION
YES	ST	FW	LT	MD	UNSUCCESFULL
YES	ST	BW	LT	MD	CONTINUED POSSESSION
YES	ST	BW	RT	CE	CONTINUED POSSESSION
NO	ST	BW	RT	CE	CONTINUED POSSESSION
YES	ST	BW	LT	OF	CONTINUED POSSESSION
YES	ST	BW	LT	OF	CONTINUED POSSESSION
YES	ME	FW	RT	CE	CONTINUED POSSESSION
YES	ME	FW	LT	DF	CONTINUED POSSESSION
YES	ME	FW	RT	MD	CONTINUED POSSESSION
YES	ST	FW	LT	MD	CONTINUED POSSESSION
YES	ST	FW	LT	MO	CONTINUED POSSESSION
YES	ST	BW	RT	OF	CONTINUED POSSESSION
YES	ME	BW	RT	CE	CONTINUED POSSESSION
YES	ME	FW	LT	CE	CONTINUED POSSESSION
YES	ME	FW	LT	MO	SUCCESFULL
YES	ST	BW	RT	DF	LOST POSSESSION

YES	ST	BW	LT	MD	CONTINUED POSSESSION
YES	ST	BW	LT	MO	LOST POSSESSION
YES	ST	FW	RT	MO	LOST POSSESSION
YES	ST	FW	LT	MD	CONTINUED POSSESSION
YES	ST	BW	LT	MO	CONTINUED POSSESSION
YES	ST	BW	RT	CE	CONTINUED POSSESSION
YES	ST	BW	LT	OF	LOST POSSESSION
YES	ST	BW	LT	MD	LOST POSSESSION
YES	ST	BW	RT	OF	CONTINUED POSSESSION
YES	LG	FW	RT	MD	LOST POSSESSION
YES	ME	FW	LT	CE	CONTINUED POSSESSION
YES	ME	FW	LT	MO	CONTINUED POSSESSION
YES	ME	FW	LT	MD	LOST POSSESSION
YES	ME	FW	RT	MO	LOST POSSESSION
YES	ME	FW	RT	CE	LOST POSSESSION
YES	ME	FW	LT	DF	LOST POSSESSION
YES	ST	FW	LT	DF	LOST POSSESSION
YES	ST	FW	RT	CE	LOST POSSESSION
YES	ST	BW	LT	MO	SUCCESFULL
YES	ME	BW	RT	MD	LOST POSSESSION
YES	ME	FW	LT	DF	LOST POSSESSION
YES	ST	FW	RT	CE	CONTINUED POSSESSION
YES	ME	FW	RT	CE	SUCCESFULL
YES	ME	FW	RT	MD	LOST POSSESSION
YES	ME	FW	RT	OF	CONTINUED POSSESSION
YES	ST	BW	RT	CE	LOST POSSESSION
YES	ST	BW	RT	OF	CONTINUED POSSESSION
YES	ST	BW	RT	OF	LOST POSSESSION
YES	ST	BW	LT	MO	LOST POSSESSION
YES	ST	FW	RT	MD	LOST POSSESSION
YES	ST	BW	RT	OF	CONTINUED POSSESSION
YES	ST	FW	RT	MO	SUCCESFULL
YES	ST	FW	RT	MO	CONTINUED POSSESSION
YES	ME	BW	RT	OF	SUCCESFULL
YES	ST	BW	RT	MO	CONTINUED POSSESSION
YES	ST	FW	RT	MO	SUCCESFULL
YES	ME	FW	LT	MD	LOST POSSESSION
YES	ST	BW	RT	OF	LOST POSSESSION
YES	LG	FW	RT	CE	LOST POSSESSION
YES	ST	FW	RT	MO	CONTINUED POSSESSION
YES	ST	BW	RT	OF	LOST POSSESSION
YES	LG	FW	RT	MO	LOST POSSESSION
YES	LG	FW	RT	MD	CONTINUED POSSESSION
YES	LG	FW	RT	OF	CONTINUED POSSESSION
YES	ST	FW	LT	MO	LOST POSSESSION
YES	ST	FW	RT	MD	CONTINUED POSSESSION
YES	LG	FW	RT	DF	CONTINUED POSSESSION
YES	ST	BW	RT	OF	LOST POSSESSION
YES	LG	FW	RT	CE	CONTINUED POSSESSION
YES	ST	BW	LT	DF	LOST POSSESSION

YES	ME	FW	LT	MO	LOST POSSESSION
YES	ME	FW	LT	CE	CONTINUED POSSESSION
YES	ME	FW	LT	MD	CONTINUED POSSESSION
YES	LG	FW	RT	MD	LOST POSSESSION
YES	ST	FW	RT	MO	LOST POSSESSION
YES	LG	FW	LT	MO	CONTINUED POSSESSION
YES	LG	FW	LT	CE	LOST POSSESSION
YES	LG	FW	LT	OF	CONTINUED POSSESSION
YES	ME	FW	LT	MO	LOST POSSESSION
YES	ST	FW	RT	MO	CONTINUED POSSESSION
YES	ST	FW	RT	MO	SUCCESFULL
YES	LG	FW	LT	MO	LOST POSSESSION
YES	ME	FW	LT	MO	CONTINUED POSSESSION
YES	LG	FW	RT	CE	CONTINUED POSSESSION
YES	ST	BW	RT	MD	LOST POSSESSION
YES	ST	FW	RT	MD	CONTINUED POSSESSION
YES	ST	FW	RT	CE	CONTINUED POSSESSION
YES	ST	BW	LT	CE	LOST POSSESSION
YES	LG	FW	RT	MD	CONTINUED POSSESSION
YES	ST	BW	LT	MD	SUCCESFULL
YES	ST	FW	LT	MO	LOST POSSESSION
YES	ST	BW	RT	CE	CONTINUED POSSESSION
YES	LG	FW	RT	CE	LOST POSSESSION
YES	LG	FW	RT	MD	CONTINUED POSSESSION
YES	ME	FW	RT	MD	LOST POSSESSION
YES	ST	BW	LT	CE	LOST POSSESSION
YES	ST	FW	LT	MO	CONTINUED POSSESSION
YES	LG	FW	RT	CE	CONTINUED POSSESSION
YES	ST	BW	LT	MO	CONTINUED POSSESSION
YES	ME	BW	LT	MO	CONTINUED POSSESSION
YES	ST	BW	LT	OF	CONTINUED POSSESSION
YES	ST	BW	RT	CE	CONTINUED POSSESSION
YES	ST	BW	RT	MD	CONTINUED POSSESSION
YES	ST	BW	LT	MO	CONTINUED POSSESSION
YES	ME	BW	LT	MD	CONTINUED POSSESSION
YES	ST	FW	LT	MD	CONTINUED POSSESSION
YES	ST	BW	RT	OF	CONTINUED POSSESSION
YES	ST	FW	LT	DF	LOST POSSESSION
YES	ST	FW	RT	MD	CONTINUED POSSESSION
YES	ME	BW	LT	CE	CONTINUED POSSESSION
NO	ME	BW	LT	MO	CONTINUED POSSESSION
YES	ST	BW	LT	CE	SUCCESFULL
YES	ST	BW	RT	MO	CONTINUED POSSESSION
YES	ST	FW	RT	MD	CONTINUED POSSESSION
YES	ST	FW	LT	MD	CONTINUED POSSESSION
YES	ST	FW	RT	CE	CONTINUED POSSESSION
YES	ME	FW	LT	DF	LOST POSSESSION
YES	ME	BW	RT	CE	CONTINUED POSSESSION
YES	ST	BW	RT	OF	CONTINUED POSSESSION
YES	ST	BW	LT	MO	CONTINUED POSSESSION

YES	ME	FW	RT	MD	LOST POSSESSION
YES	ME	BW	LT	MD	CONTINUED POSSESSION
YES	ME	FW	RT	MO	LOST POSSESSION
YES	ME	FW	RT	MD	LOST POSSESSION
YES	ST	FW	RT	MO	CONTINUED POSSESSION
YES	ST	BW	LT	CE	CONTINUED POSSESSION
NO	ST	BW	RT	MO	CONTINUED POSSESSION
YES	ST	BW	LT	MO	SUCCESFULL
YES	ST	FW	LT	CE	CONTINUED POSSESSION
YES	ST	BW	LT	MO	CONTINUED POSSESSION
YES	ST	FW	RT	MD	CONTINUED POSSESSION
YES	ST	BW	RT	MD	CONTINUED POSSESSION
YES	ME	BW	LT	OF	CONTINUED POSSESSION
NO	ST	BW	RT	CE	CONTINUED POSSESSION
YES	ST	BW	LT	OF	SUCCESFULL
YES	ST	FW	LT	OF	CONTINUED POSSESSION
YES	ME	BW	RT	OF	CONTINUED POSSESSION
YES	ST	BW	LT	CE	CONTINUED POSSESSION
YES	ME	BW	RT	CE	CONTINUED POSSESSION
YES	ME	BW	RT	MO	SUCCESFULL
YES	ST	BW	LT	MO	CONTINUED POSSESSION
YES	ST	FW	LT	MO	SUCCESFULL
YES	ST	FW	LT	MD	LOST POSSESSION
YES	ST	BW	LT	MO	CONTINUED POSSESSION
YES	ME	BW	RT	CE	CONTINUED POSSESSION
YES	ST	BW	LT	DF	CONTINUED POSSESSION
YES	ST	BW	RT	MO	CONTINUED POSSESSION
YES	ST	BW	LT	OF	LOST POSSESSION
YES	ST	FW	LT	MO	LOST POSSESSION
YES	ME	FW	LT	MD	LOST POSSESSION
NO	ST	BW	LT	MO	CONTINUED POSSESSION
YES	ST	BW	RT	MD	CONTINUED POSSESSION
YES	ST	BW	RT	MO	CONTINUED POSSESSION
YES	ST	FW	LT	CE	UNSUCCESFULL
YES	ME	BW	RT	MD	CONTINUED POSSESSION
YES	LG	BW	RT	CE	SUCCESFULL
YES	ME	BW	LT	CE	CONTINUED POSSESSION
YES	ME	BW	RT	MO	SUCCESFULL
YES	ST	FW	LT	MD	CONTINUED POSSESSION
YES	ME	BW	RT	MD	CONTINUED POSSESSION
YES	ST	BW	LT	MO	CONTINUED POSSESSION
YES	ST	BW	RT	MO	CONTINUED POSSESSION
YES	ST	BW	RT	MD	CONTINUED POSSESSION
YES	ME	FW	LT	MD	LOST POSSESSION
YES	ST	BW	RT	OF	CONTINUED POSSESSION
YES	ME	BW	RT	OF	SUCCESFULL
YES	ST	FW	LT	DF	LOST POSSESSION
YES	ME	FW	LT	CE	LOST POSSESSION
NO	ST	BW	LT	MD	CONTINUED POSSESSION
YES	ME	BW	LT	MD	CONTINUED POSSESSION

YES	ST	FW	RT	MD	CONTINUED POSSESSION
NO	ST	BW	LT	MD	CONTINUED POSSESSION
YES	ME	BW	RT	CE	CONTINUED POSSESSION
YES	ME	FW	RT	MD	CONTINUED POSSESSION
YES	ST	BW	RT	CE	CONTINUED POSSESSION
YES	ST	FW	LT	MD	SUCCESFULL
YES	ST	FW	LT	MD	LOST POSSESSION
YES	ST	FW	LT	MO	CONTINUED POSSESSION
YES	ME	BW	RT	MO	CONTINUED POSSESSION
YES	ST	BW	RT	OF	CONTINUED POSSESSION
YES	ME	FW	RT	MD	LOST POSSESSION
YES	ME	BW	RT	OF	LOST POSSESSION
YES	ST	BW	RT	MO	CONTINUED POSSESSION
YES	ST	BW	RT	MO	CONTINUED POSSESSION
YES	ST	BW	RT	MO	CONTINUED POSSESSION
YES	ST	BW	LT	MD	LOST POSSESSION
YES	ST	BW	LT	MO	LOST POSSESSION
YES	ST	FW	RT	MO	CONTINUED POSSESSION
YES	ST	FW	RT	MD	CONTINUED POSSESSION
YES	ST	BW	RT	MD	LOST POSSESSION
YES	ME	FW	RT	MD	SUCCESFULL
YES	ST	FW	RT	OF	SUCCESFULL
YES	ST	FW	LT	MO	CONTINUED POSSESSION
YES	ME	FW	RT	CE	CONTINUED POSSESSION
YES	ME	BW	LT	CE	LOST POSSESSION
YES	ME	FW	RT	MO	SUCCESFULL
NO	ST	BW	LT	MD	CONTINUED POSSESSION
YES	ME	FW	RT	MO	LOST POSSESSION
YES	ST	FW	RT	MO	CONTINUED POSSESSION
YES	ST	BW	RT	OF	LOST POSSESSION
NO	ME	BW	LT	CE	CONTINUED POSSESSION
NO	ST	BW	LT	MD	CONTINUED POSSESSION
YES	ST	FW	RT	MD	LOST POSSESSION
YES	ST	FW	RT	MD	LOST POSSESSION
YES	ST	BW	LT	DF	LOST POSSESSION
NO	ME	BW	LT	MD	CONTINUED POSSESSION
YES	ME	FW	LT	CE	CONTINUED POSSESSION
YES	ME	BW	RT	DF	SUCCESFULL
YES	ME	FW	RT	CE	CONTINUED POSSESSION
YES	ME	FW	LT	MO	CONTINUED POSSESSION
YES	ST	FW	LT	MO	CONTINUED POSSESSION
YES	ST	FW	RT	CE	UNSUCCESFULL
YES	ST	BW	LT	MO	CONTINUED POSSESSION
YES	ST	FW	LT	OF	CONTINUED POSSESSION
YES	ST	FW	LT	MD	CONTINUED POSSESSION
YES	ST	BW	LT	OF	LOST POSSESSION
YES	ST	FW	LT	MO	CONTINUED POSSESSION
NO	ME	BW	RT	MO	CONTINUED POSSESSION
YES	ST	FW	LT	MO	LOST POSSESSION
YES	ST	FW	RT	MD	LOST POSSESSION

YES	ST	FW	LT	OF	LOST POSSESSION
YES	ME	BW	RT	MO	CONTINUED POSSESSION
YES	ST	FW	LT	MD	CONTINUED POSSESSION
YES	ST	BW	RT	OF	CONTINUED POSSESSION
YES	ME	FW	RT	CE	SUCCESFULL
YES	ME	FW	RT	MO	CONTINUED POSSESSION
YES	ME	FW	RT	MD	LOST POSSESSION
YES	ST	BW	RT	MO	CONTINUED POSSESSION
YES	ST	BW	RT	MO	SUCCESFULL
YES	ST	BW	RT	CE	CONTINUED POSSESSION
YES	LG	FW	RT	DF	LOST POSSESSION
YES	ST	FW	RT	MD	SUCCESFULL
YES	ME	BW	LT	OF	CONTINUED POSSESSION
YES	ME	BW	LT	CE	CONTINUED POSSESSION
YES	ST	FW	LT	DF	CONTINUED POSSESSION
YES	ME	BW	LT	MD	CONTINUED POSSESSION
YES	ST	FW	RT	MO	CONTINUED POSSESSION
YES	ST	BW	LT	CE	CONTINUED POSSESSION
YES	ME	BW	LT	MO	CONTINUED POSSESSION
YES	ST	FW	RT	DF	CONTINUED POSSESSION
YES	ST	FW	RT	MD	LOST POSSESSION
YES	ME	BW	LT	CE	CONTINUED POSSESSION
YES	ME	BW	LT	CE	CONTINUED POSSESSION
YES	ST	BW	LT	MO	CONTINUED POSSESSION
YES	ME	FW	LT	MD	CONTINUED POSSESSION
YES	ME	FW	LT	MD	LOST POSSESSION
YES	ME	BW	LT	MO	CONTINUED POSSESSION
YES	ME	FW	RT	MO	LOST POSSESSION
YES	ST	BW	LT	CE	CONTINUED POSSESSION
YES	ME	FW	RT	MD	LOST POSSESSION
YES	LG	FW	LT	OF	LOST POSSESSION
YES	LG	FW	LT	MD	LOST POSSESSION
YES	ST	FW	RT	MO	CONTINUED POSSESSION
YES	ME	FW	RT	MD	LOST POSSESSION
YES	LG	BW	LT	CE	CONTINUED POSSESSION
YES	ME	BW	LT	MO	CONTINUED POSSESSION
YES	ME	FW	RT	MD	CONTINUED POSSESSION
YES	ST	FW	RT	MD	CONTINUED POSSESSION
YES	ST	BW	RT	CE	CONTINUED POSSESSION
YES	ST	FW	RT	MO	LOST POSSESSION
YES	ST	BW	RT	MO	LOST POSSESSION
YES	LG	FW	LT	CE	CONTINUED POSSESSION
YES	LG	FW	LT	MO	LOST POSSESSION
YES	ST	BW	RT	MO	CONTINUED POSSESSION
YES	ME	FW	RT	MD	LOST POSSESSION
YES	ST	BW	RT	OF	CONTINUED POSSESSION
YES	ME	BW	LT	MD	CONTINUED POSSESSION
YES	ME	BW	RT	CE	CONTINUED POSSESSION
YES	ST	FW	LT	CE	LOST POSSESSION
YES	ME	BW	RT	OF	LOST POSSESSION

YES	ST	BW	RT	MO	CONTINUED POSSESSION
YES	ME	FW	LT	MD	LOST POSSESSION
NO	ME	BW	LT	MD	CONTINUED POSSESSION
NO	ME	BW	LT	CE	CONTINUED POSSESSION
YES	ST	FW	LT	MD	LOST POSSESSION
YES	ME	FW	LT	MO	LOST POSSESSION
YES	ST	BW	LT	MO	SUCCESFULL
YES	ME	FW	RT	MD	LOST POSSESSION
YES	ST	FW	LT	MD	CONTINUED POSSESSION
YES	ME	FW	RT	CE	LOST POSSESSION
YES	ST	FW	LT	MO	CONTINUED POSSESSION
NO	LG	BW	LT	DF	LOST POSSESSION
YES	ME	BW	RT	MD	LOST POSSESSION
YES	ME	FW	RT	MD	LOST POSSESSION
YES	ME	BW	RT	MO	CONTINUED POSSESSION
YES	LG	FW	LT	MO	CONTINUED POSSESSION
YES	LG	FW	LT	MO	CONTINUED POSSESSION
YES	ME	BW	LT	CE	LOST POSSESSION
YES	LG	FW	LT	MD	LOST POSSESSION
YES	ME	FW	RT	MO	CONTINUED POSSESSION
YES	LG	FW	LT	MO	LOST POSSESSION
YES	ME	FW	RT	MD	CONTINUED POSSESSION
YES	ST	FW	RT	MO	LOST POSSESSION
YES	ME	FW	LT	CE	LOST POSSESSION
NO	ME	FW	RT	CE	LOST POSSESSION
YES	ME	BW	LT	CE	CONTINUED POSSESSION
YES	ST	FW	LT	MD	LOST POSSESSION
YES	ME	BW	RT	CE	CONTINUED POSSESSION
YES	ME	FW	LT	MO	CONTINUED POSSESSION
YES	ME	FW	RT	MO	CONTINUED POSSESSION
YES	LG	BW	LT	OF	CONTINUED POSSESSION
YES	ST	BW	RT	MO	CONTINUED POSSESSION
YES	ME	BW	LT	MD	CONTINUED POSSESSION
YES	LG	FW	RT	MD	UNSUCCESFULL
YES	ST	BW	LT	MD	CONTINUED POSSESSION
YES	ME	BW	LT	CE	CONTINUED POSSESSION
YES	ME	FW	RT	MD	CONTINUED POSSESSION
YES	ST	FW	RT	CE	CONTINUED POSSESSION
YES	ME	BW	LT	MD	CONTINUED POSSESSION
YES	LG	FW	LT	CE	CONTINUED POSSESSION
YES	ST	FW	LT	MO	CONTINUED POSSESSION
YES	ST	FW	LT	MD	CONTINUED POSSESSION
YES	ME	FW	LT	MD	LOST POSSESSION
NO	ST	BW	LT	MD	CONTINUED POSSESSION
YES	ST	BW	RT	MO	CONTINUED POSSESSION
NO	LG	BW	RT	MO	CONTINUED POSSESSION
YES	ST	FW	RT	MO	CONTINUED POSSESSION
YES	ME	BW	LT	CE	CONTINUED POSSESSION
YES	LG	BW	RT	MD	CONTINUED POSSESSION
YES	ME	BW	LT	OF	SUCCESFULL

YES	ME	FW	RT	MO	LOST POSSESSION
YES	ST	BW	LT	MO	CONTINUED POSSESSION
YES	ST	BW	RT	MO	SUCCESFULL
YES	ST	BW	LT	MD	CONTINUED POSSESSION
YES	ST	BW	LT	MD	CONTINUED POSSESSION
NO	ME	BW	RT	CE	CONTINUED POSSESSION
YES	ST	BW	LT	MD	CONTINUED POSSESSION
YES	ST	BW	LT	MO	UNSUCCESFULL
NO	ST	BW	LT	MO	CONTINUED POSSESSION
YES	LG	BW	LT	CE	CONTINUED POSSESSION
YES	ME	BW	RT	CE	CONTINUED POSSESSION
NO	ME	BW	LT	MD	CONTINUED POSSESSION
YES	ME	FW	RT	MO	LOST POSSESSION
YES	LG	FW	LT	DF	LOST POSSESSION
YES	ST	BW	RT	CE	CONTINUED POSSESSION
YES	ST	BW	RT	OF	LOST POSSESSION
YES	ME	BW	RT	CE	CONTINUED POSSESSION
YES	ST	BW	RT	OF	CONTINUED POSSESSION
YES	ST	BW	LT	MD	CONTINUED POSSESSION
YES	ST	BW	LT	OF	CONTINUED POSSESSION
YES	ME	BW	LT	MO	SUCCESFULL
YES	ME	BW	RT	CE	LOST POSSESSION
YES	ST	BW	RT	OF	CONTINUED POSSESSION
YES	ST	BW	LT	MO	CONTINUED POSSESSION
YES	ME	FW	LT	MD	UNSUCCESFULL
YES	ST	FW	RT	OF	LOST POSSESSION
YES	ME	FW	LT	CE	CONTINUED POSSESSION
YES	ST	BW	LT	OF	CONTINUED POSSESSION
YES	ST	BW	RT	OF	CONTINUED POSSESSION
YES	ME	BW	RT	MD	CONTINUED POSSESSION
YES	ST	FW	RT	MD	LOST POSSESSION
YES	ME	BW	RT	MD	CONTINUED POSSESSION
YES	ST	FW	RT	MO	CONTINUED POSSESSION
YES	ME	FW	RT	OF	SUCCESFULL
YES	ME	BW	RT	MO	CONTINUED POSSESSION
YES	ME	FW	RT	MD	LOST POSSESSION
YES	ME	BW	LT	MD	CONTINUED POSSESSION
YES	ME	FW	LT	CE	CONTINUED POSSESSION
YES	ME	FW	LT	CE	LOST POSSESSION
YES	ME	FW	LT	MD	CONTINUED POSSESSION
YES	ST	FW	RT	OF	SUCCESFULL
YES	ME	FW	LT	MO	CONTINUED POSSESSION
YES	ME	FW	LT	MO	LOST POSSESSION
YES	ST	FW	LT	MO	CONTINUED POSSESSION
YES	ST	FW	LT	MO	CONTINUED POSSESSION
YES	ST	FW	LT	MD	CONTINUED POSSESSION
YES	ME	FW	RT	CE	LOST POSSESSION
YES	ST	BW	LT	OF	LOST POSSESSION
YES	ME	BW	RT	OF	LOST POSSESSION
YES	ST	BW	RT	CE	SUCCESFULL

NO	ME	BW	RT	MD	CONTINUED POSSESSION
YES	ME	FW	RT	MO	CONTINUED POSSESSION
YES	ST	BW	LT	CE	SUCCESFULL
YES	ME	FW	LT	MO	CONTINUED POSSESSION
YES	ME	BW	LT	MD	CONTINUED POSSESSION
YES	ST	BW	LT	OF	CONTINUED POSSESSION
YES	ME	BW	LT	MO	CONTINUED POSSESSION
YES	ST	FW	LT	OF	LOST POSSESSION
YES	ME	FW	LT	MO	LOST POSSESSION
YES	ME	FW	RT	CE	LOST POSSESSION
YES	ME	BW	RT	CE	CONTINUED POSSESSION
YES	ST	FW	LT	OF	CONTINUED POSSESSION
YES	ME	FW	LT	MO	CONTINUED POSSESSION
YES	ST	FW	LT	OF	LOST POSSESSION
YES	LG	FW	LT	MD	LOST POSSESSION
YES	ST	FW	RT	CE	CONTINUED POSSESSION
NO	ME	FW	RT	MD	LOST POSSESSION
YES	ME	FW	LT	CE	CONTINUED POSSESSION
YES	ST	FW	RT	CE	CONTINUED POSSESSION
YES	ME	FW	LT	CE	CONTINUED POSSESSION
YES	ME	FW	RT	CE	CONTINUED POSSESSION
YES	ME	BW	RT	OF	LOST POSSESSION
YES	ME	FW	LT	MD	SUCCESFULL
YES	ME	FW	RT	MO	LOST POSSESSION
YES	ST	BW	RT	MO	LOST POSSESSION
YES	ME	FW	LT	MD	CONTINUED POSSESSION
YES	ME	BW	LT	MO	CONTINUED POSSESSION
YES	ME	BW	RT	MD	CONTINUED POSSESSION
YES	ST	BW	LT	MO	CONTINUED POSSESSION
YES	ME	FW	LT	MO	LOST POSSESSION
YES	ME	BW	LT	CE	CONTINUED POSSESSION
YES	ME	FW	RT	CE	LOST POSSESSION
YES	ME	BW	LT	MO	LOST POSSESSION
YES	LG	FW	LT	CE	CONTINUED POSSESSION
YES	ST	FW	LT	MO	LOST POSSESSION
YES	ME	FW	RT	MD	LOST POSSESSION
YES	ME	FW	LT	MD	LOST POSSESSION
YES	ME	BW	RT	MO	CONTINUED POSSESSION
YES	ME	FW	RT	MO	LOST POSSESSION
YES	ME	FW	LT	MD	CONTINUED POSSESSION
NO	LG	FW	LT	CE	LOST POSSESSION
YES	ME	FW	LT	MO	CONTINUED POSSESSION
YES	ST	BW	LT	MO	CONTINUED POSSESSION
YES	ST	FW	RT	MO	CONTINUED POSSESSION
YES	ST	BW	RT	MO	CONTINUED POSSESSION
YES	ME	FW	LT	OF	CONTINUED POSSESSION
YES	ME	FW	RT	CE	LOST POSSESSION
YES	ME	FW	LT	MD	CONTINUED POSSESSION
YES	ME	FW	LT	MD	CONTINUED POSSESSION
YES	ST	FW	LT	CE	CONTINUED POSSESSION

YES	ME	FW	RT	MO	CONTINUED POSSESSION
YES	ME	FW	RT	CE	LOST POSSESSION
NO	ST	BW	LT	MD	UNSUCCESFULL
YES	ST	FW	LT	OF	LOST POSSESSION
YES	ME	FW	RT	MD	CONTINUED POSSESSION
YES	LG	BW	RT	CE	LOST POSSESSION
YES	ME	FW	LT	CE	CONTINUED POSSESSION
YES	ST	BW	LT	CE	CONTINUED POSSESSION
YES	ST	FW	LT	CE	CONTINUED POSSESSION
YES	ME	FW	LT	MO	SUCCESFULL
YES	ME	FW	RT	DF	LOST POSSESSION
YES	ME	FW	RT	MD	CONTINUED POSSESSION
YES	ST	FW	LT	MD	CONTINUED POSSESSION
YES	ST	FW	RT	CE	CONTINUED POSSESSION
YES	ST	FW	LT	MO	LOST POSSESSION
YES	ST	FW	LT	OF	LOST POSSESSION
NO	ME	BW	LT	CE	CONTINUED POSSESSION
YES	ST	BW	RT	MO	LOST POSSESSION
YES	ME	BW	RT	CE	CONTINUED POSSESSION
YES	ST	FW	LT	OF	LOST POSSESSION
YES	ME	FW	RT	MD	LOST POSSESSION
YES	ST	FW	RT	CE	CONTINUED POSSESSION
YES	ME	FW	LT	CE	CONTINUED POSSESSION
YES	ME	BW	LT	MO	LOST POSSESSION
YES	ST	FW	LT	CE	CONTINUED POSSESSION
YES	ST	BW	LT	CE	LOST POSSESSION
YES	ME	FW	LT	CE	LOST POSSESSION
YES	ST	FW	LT	OF	LOST POSSESSION
YES	ME	BW	LT	CE	CONTINUED POSSESSION
YES	LG	FW	LT	CE	CONTINUED POSSESSION
YES	ST	FW	RT	CE	LOST POSSESSION
YES	LG	FW	LT	MD	LOST POSSESSION
YES	LG	FW	RT	CE	LOST POSSESSION
YES	ME	BW	LT	CE	CONTINUED POSSESSION
YES	LG	FW	LT	MD	LOST POSSESSION
YES	ME	BW	LT	CE	CONTINUED POSSESSION
YES	ME	FW	RT	MD	CONTINUED POSSESSION
YES	ME	FW	RT	CE	CONTINUED POSSESSION
YES	ST	BW	RT	DF	LOST POSSESSION
YES	ME	FW	RT	MO	CONTINUED POSSESSION
NO	ST	BW	LT	CE	CONTINUED POSSESSION
YES	ST	BW	LT	MO	LOST POSSESSION
YES	ME	FW	RT	MD	LOST POSSESSION
YES	ST	FW	LT	OF	CONTINUED POSSESSION
YES	ST	FW	LT	OF	LOST POSSESSION
YES	ME	FW	LT	MD	LOST POSSESSION
YES	ME	FW	LT	MD	CONTINUED POSSESSION
YES	ST	FW	LT	CE	CONTINUED POSSESSION
YES	ME	FW	RT	DF	LOST POSSESSION
YES	ST	BW	LT	OF	CONTINUED POSSESSION

YES	LG	FW	RT	OF	CONTINUED POSSESSION
YES	ME	FW	RT	MD	LOST POSSESSION
YES	ME	FW	RT	MO	CONTINUED POSSESSION
YES	LG	FW	RT	CE	LOST POSSESSION
YES	ME	FW	LT	DF	LOST POSSESSION
YES	LG	FW	RT	MO	CONTINUED POSSESSION
YES	LG	FW	RT	CE	LOST POSSESSION
YES	ST	FW	RT	MD	LOST POSSESSION
YES	ME	FW	LT	CE	LOST POSSESSION
YES	ST	FW	LT	OF	CONTINUED POSSESSION
YES	LG	FW	LT	MO	SUCCESFULL
YES	ME	FW	LT	CE	LOST POSSESSION
YES	ME	FW	LT	CE	LOST POSSESSION
YES	ME	BW	RT	MO	LOST POSSESSION
YES	LG	FW	RT	OF	LOST POSSESSION
YES	ME	FW	RT	MD	LOST POSSESSION
YES	LG	FW	LT	MD	CONTINUED POSSESSION
YES	LG	FW	LT	OF	CONTINUED POSSESSION
YES	LG	FW	LT	CE	LOST POSSESSION
YES	ME	BW	LT	CE	CONTINUED POSSESSION
YES	LG	FW	RT	MO	LOST POSSESSION
YES	ST	BW	LT	CE	CONTINUED POSSESSION
YES	ST	BW	LT	MO	LOST POSSESSION
YES	ME	BW	LT	MO	CONTINUED POSSESSION
YES	LG	FW	RT	OF	SUCCESFULL
YES	ME	BW	RT	MD	CONTINUED POSSESSION
YES	ST	BW	LT	CE	CONTINUED POSSESSION
YES	ST	BW	RT	MD	CONTINUED POSSESSION
YES	ME	BW	RT	CE	CONTINUED POSSESSION
YES	ST	BW	RT	MD	CONTINUED POSSESSION
YES	LG	FW	RT	MD	SUCCESFULL
YES	LG	FW	RT	MO	LOST POSSESSION
YES	LG	FW	RT	OF	LOST POSSESSION
YES	ST	BW	LT	CE	CONTINUED POSSESSION
YES	LG	FW	RT	MO	CONTINUED POSSESSION
YES	ME	FW	RT	CE	CONTINUED POSSESSION
YES	ST	BW	RT	CE	CONTINUED POSSESSION
YES	ME	BW	LT	CE	CONTINUED POSSESSION
YES	LG	FW	RT	CE	LOST POSSESSION
YES	ME	FW	RT	CE	LOST POSSESSION
YES	ME	FW	RT	DF	CONTINUED POSSESSION
YES	ME	FW	RT	MO	LOST POSSESSION
YES	ST	FW	RT	OF	LOST POSSESSION
YES	ME	FW	LT	MD	LOST POSSESSION
YES	ST	BW	RT	MO	LOST POSSESSION
YES	LG	FW	LT	MO	LOST POSSESSION
YES	ME	BW	RT	OF	LOST POSSESSION
YES	ST	FW	LT	MO	CONTINUED POSSESSION
YES	ST	BW	LT	MO	CONTINUED POSSESSION
YES	ME	FW	RT	MO	CONTINUED POSSESSION

YES	ST	BW	RT	CE	CONTINUED POSSESSION
YES	ME	BW	LT	CE	CONTINUED POSSESSION
YES	ST	FW	LT	MD	CONTINUED POSSESSION
YES	ME	FW	RT	MO	LOST POSSESSION
YES	ST	BW	RT	OF	SUCCESFULL
YES	ME	FW	LT	CE	LOST POSSESSION
YES	ST	FW	LT	MO	LOST POSSESSION
YES	ME	FW	LT	CE	LOST POSSESSION
YES	ME	FW	LT	CE	SUCCESFULL
YES	ME	FW	LT	CE	LOST POSSESSION
YES	ST	FW	RT	MO	LOST POSSESSION
YES	LG	FW	LT	MO	LOST POSSESSION
YES	ME	FW	RT	CE	CONTINUED POSSESSION
YES	ME	BW	LT	CE	LOST POSSESSION
YES	ST	FW	RT	MO	LOST POSSESSION
YES	ME	FW	RT	MD	CONTINUED POSSESSION
YES	ME	FW	LT	MD	CONTINUED POSSESSION
YES	ME	FW	LT	MD	LOST POSSESSION
YES	ME	FW	LT	MD	LOST POSSESSION
YES	ME	FW	LT	MD	LOST POSSESSION
YES	ME	FW	RT	MO	CONTINUED POSSESSION
YES	ME	FW	RT	MO	CONTINUED POSSESSION
YES	ST	FW	LT	OF	LOST POSSESSION
YES	ME	FW	LT	MO	LOST POSSESSION
YES	ME	FW	RT	MO	CONTINUED POSSESSION
YES	ME	FW	RT	MO	CONTINUED POSSESSION
YES	ST	FW	LT	MO	CONTINUED POSSESSION
YES	ME	BW	LT	MO	CONTINUED POSSESSION
YES	ST	FW	LT	OF	LOST POSSESSION
YES	ST	FW	LT	MO	CONTINUED POSSESSION
YES	ME	FW	LT	CE	LOST POSSESSION
YES	ME	FW	LT	MD	LOST POSSESSION
YES	ME	FW	RT	CE	CONTINUED POSSESSION
YES	ME	FW	LT	MO	LOST POSSESSION
YES	ME	FW	LT	MD	LOST POSSESSION
YES	LG	FW	LT	CE	CONTINUED POSSESSION
YES	ME	FW	RT	MD	LOST POSSESSION
YES	ST	FW	LT	OF	LOST POSSESSION
YES	ME	FW	LT	MD	CONTINUED POSSESSION
YES	ME	FW	LT	CE	CONTINUED POSSESSION
YES	ME	FW	LT	OF	LOST POSSESSION
YES	LG	FW	LT	MD	LOST POSSESSION
YES	LG	BW	RT	MD	LOST POSSESSION
YES	ME	FW	LT	MD	CONTINUED POSSESSION
YES	LG	FW	LT	CE	LOST POSSESSION
YES	ME	FW	LT	MD	LOST POSSESSION
YES	LG	FW	RT	DF	LOST POSSESSION
YES	ST	FW	LT	MD	SUCCESFULL
YES	ME	FW	RT	CE	LOST POSSESSION
YES	ST	FW	RT	OF	CONTINUED POSSESSION

YES	ST	FW	LT	MD	CONTINUED POSSESSION
YES	ME	FW	LT	MD	LOST POSSESSION
YES	ME	FW	LT	MO	SUCCESFULL
YES	ST	FW	LT	OF	LOST POSSESSION
YES	ST	FW	LT	MO	CONTINUED POSSESSION
YES	ME	FW	LT	CE	SUCCESFULL
YES	ME	FW	LT	MO	CONTINUED POSSESSION
YES	ST	FW	LT	MO	CONTINUED POSSESSION
YES	ST	FW	LT	OF	CONTINUED POSSESSION
YES	ST	FW	RT	OF	LOST POSSESSION
YES	ST	FW	LT	OF	SUCCESFULL
YES	ST	FW	RT	OF	LOST POSSESSION
YES	ME	BW	RT	DF	LOST POSSESSION
NO	ST	BW	RT	CE	CONTINUED POSSESSION
YES	ME	BW	RT	DF	CONTINUED POSSESSION
YES	ST	FW	LT	OF	LOST POSSESSION
YES	ME	FW	LT	DF	LOST POSSESSION
YES	ME	FW	LT	MD	LOST POSSESSION
YES	ST	FW	RT	CE	UNSUCCESFULL
YES	ME	FW	LT	MD	CONTINUED POSSESSION
YES	LG	FW	RT	MD	LOST POSSESSION
YES	ME	FW	RT	MD	LOST POSSESSION
YES	ME	BW	RT	MD	CONTINUED POSSESSION
YES	ME	FW	RT	CE	LOST POSSESSION
YES	ME	BW	RT	CE	CONTINUED POSSESSION
YES	LG	FW	RT	DF	LOST POSSESSION
YES	ME	BW	RT	MD	CONTINUED POSSESSION
YES	LG	BW	RT	CE	CONTINUED POSSESSION
YES	ME	BW	LT	CE	CONTINUED POSSESSION
YES	ST	BW	RT	CE	CONTINUED POSSESSION
YES	ME	FW	RT	MO	CONTINUED POSSESSION
YES	LG	BW	RT	CE	CONTINUED POSSESSION
YES	ST	BW	RT	MO	SUCCESFULL
YES	LG	FW	LT	MO	LOST POSSESSION
YES	ME	BW	LT	MO	LOST POSSESSION
YES	ME	BW	LT	MO	CONTINUED POSSESSION
YES	ST	FW	RT	MO	CONTINUED POSSESSION
YES	ST	FW	RT	MO	LOST POSSESSION
YES	LG	FW	LT	MO	CONTINUED POSSESSION
YES	LG	FW	LT	MO	LOST POSSESSION
YES	ME	FW	LT	MO	LOST POSSESSION
YES	ST	FW	LT	CE	CONTINUED POSSESSION
YES	ST	FW	RT	MD	CONTINUED POSSESSION
YES	ST	FW	LT	MO	LOST POSSESSION
YES	ME	BW	LT	CE	CONTINUED POSSESSION
YES	ME	FW	LT	MD	CONTINUED POSSESSION
YES	ME	BW	LT	CE	CONTINUED POSSESSION
YES	ME	FW	LT	OF	LOST POSSESSION
YES	ME	BW	LT	CE	CONTINUED POSSESSION
YES	ME	BW	LT	CE	CONTINUED POSSESSION

YES	LG	FW	RT	OF	LOST POSSESSION
YES	ST	FW	LT	CE	CONTINUED POSSESSION
YES	ME	BW	RT	CE	CONTINUED POSSESSION
YES	ME	FW	LT	MO	LOST POSSESSION
YES	LG	FW	LT	MD	LOST POSSESSION
YES	ST	FW	RT	CE	LOST POSSESSION
YES	ST	FW	RT	CE	CONTINUED POSSESSION
YES	ST	BW	LT	OF	CONTINUED POSSESSION
YES	ST	BW	LT	OF	UNSUCCESFULL
YES	ST	FW	RT	OF	CONTINUED POSSESSION
YES	ME	BW	LT	MD	CONTINUED POSSESSION
YES	ME	BW	LT	MO	LOST POSSESSION
YES	ME	BW	RT	CE	CONTINUED POSSESSION
YES	ST	BW	RT	MO	CONTINUED POSSESSION
YES	ST	FW	RT	CE	LOST POSSESSION
YES	ST	FW	RT	CE	LOST POSSESSION
YES	ST	FW	RT	MD	CONTINUED POSSESSION
YES	ST	FW	LT	MO	SUCCESFULL
YES	ST	FW	RT	MO	CONTINUED POSSESSION
YES	ST	FW	RT	OF	LOST POSSESSION
YES	ST	FW	LT	MO	LOST POSSESSION
YES	ST	FW	LT	MO	LOST POSSESSION
YES	ME	FW	LT	CE	LOST POSSESSION
YES	ST	FW	RT	OF	LOST POSSESSION
YES	ME	FW	LT	MD	LOST POSSESSION
YES	ST	FW	LT	CE	LOST POSSESSION
YES	ST	FW	RT	MD	CONTINUED POSSESSION
YES	ME	BW	RT	CE	CONTINUED POSSESSION
YES	ME	BW	LT	MD	CONTINUED POSSESSION
NO	ME	BW	RT	MD	CONTINUED POSSESSION
NO	ME	BW	RT	MD	CONTINUED POSSESSION
YES	ST	FW	LT	MD	CONTINUED POSSESSION
NO	ME	BW	RT	MD	CONTINUED POSSESSION
YES	ME	BW	RT	MD	CONTINUED POSSESSION
YES	ME	BW	LT	MD	CONTINUED POSSESSION
YES	ME	FW	RT	MD	CONTINUED POSSESSION
YES	ST	FW	RT	OF	CONTINUED POSSESSION
YES	ST	FW	LT	OF	LOST POSSESSION
YES	ME	BW	RT	OF	CONTINUED POSSESSION
YES	ME	FW	RT	OF	LOST POSSESSION
YES	ST	BW	LT	MO	CONTINUED POSSESSION
YES	ME	BW	RT	CE	CONTINUED POSSESSION
YES	ST	FW	LT	MD	CONTINUED POSSESSION
YES	ME	BW	RT	OF	CONTINUED POSSESSION
YES	ST	BW	LT	OF	SUCCESFULL
YES	ME	BW	RT	MD	CONTINUED POSSESSION
YES	ST	FW	LT	MD	UNSUCCESFULL
YES	ME	BW	RT	MO	CONTINUED POSSESSION
YES	ST	FW	RT	MO	CONTINUED POSSESSION
YES	ST	BW	LT	MD	CONTINUED POSSESSION

YES	ST	BW	LT	MO	CONTINUED POSSESSION
YES	ST	FW	RT	MO	SUCCESFULL
YES	ST	FW	RT	CE	CONTINUED POSSESSION
YES	ST	FW	RT	CE	LOST POSSESSION
YES	ST	BW	RT	MO	CONTINUED POSSESSION
YES	ST	BW	RT	MO	LOST POSSESSION
YES	ST	FW	RT	MO	LOST POSSESSION
YES	ME	FW	RT	CE	SUCCESFULL
YES	ST	FW	RT	CE	LOST POSSESSION
YES	ME	BW	RT	OF	LOST POSSESSION
YES	ST	FW	RT	MO	CONTINUED POSSESSION
YES	ME	BW	RT	OF	CONTINUED POSSESSION
YES	ST	BW	RT	MO	LOST POSSESSION
YES	ME	BW	RT	CE	LOST POSSESSION
YES	ST	FW	RT	MD	LOST POSSESSION
YES	ME	BW	RT	OF	CONTINUED POSSESSION
YES	ST	BW	RT	CE	CONTINUED POSSESSION
YES	ME	FW	LT	CE	CONTINUED POSSESSION
YES	ST	BW	LT	MD	CONTINUED POSSESSION
YES	ST	FW	LT	MO	SUCCESFULL
YES	ME	BW	RT	CE	CONTINUED POSSESSION
YES	ME	FW	RT	MO	LOST POSSESSION
YES	ME	BW	RT	MO	SUCCESFULL
YES	ME	BW	RT	CE	CONTINUED POSSESSION
YES	ST	BW	RT	OF	CONTINUED POSSESSION
YES	ME	FW	RT	DF	UNSUCCESFULL
YES	ST	BW	LT	MO	CONTINUED POSSESSION
YES	ME	BW	RT	MO	LOST POSSESSION
YES	ST	BW	LT	CE	LOST POSSESSION
YES	ME	FW	RT	OF	CONTINUED POSSESSION
YES	ST	FW	RT	MD	CONTINUED POSSESSION
YES	ME	BW	RT	MD	CONTINUED POSSESSION
YES	ST	BW	RT	MD	CONTINUED POSSESSION
YES	ST	BW	RT	MO	CONTINUED POSSESSION
YES	ST	FW	RT	CE	CONTINUED POSSESSION
YES	ST	BW	LT	MD	CONTINUED POSSESSION
YES	ME	BW	RT	MO	CONTINUED POSSESSION
YES	ST	BW	RT	MD	CONTINUED POSSESSION
YES	ST	BW	RT	OF	CONTINUED POSSESSION
YES	LG	BW	RT	OF	SUCCESFULL
YES	ST	BW	RT	MD	CONTINUED POSSESSION
YES	ST	BW	LT	MO	CONTINUED POSSESSION
YES	ST	FW	LT	MD	CONTINUED POSSESSION
YES	ME	BW	LT	CE	CONTINUED POSSESSION
YES	ME	BW	LT	CE	CONTINUED POSSESSION
YES	ME	BW	LT	MD	CONTINUED POSSESSION
YES	ST	BW	LT	MD	CONTINUED POSSESSION
YES	ST	BW	RT	MO	LOST POSSESSION
YES	ME	BW	LT	MO	SUCCESFULL
YES	ME	FW	RT	OF	CONTINUED POSSESSION

YES	ME	BW	RT	MD	CONTINUED POSSESSION
YES	ST	BW	LT	MO	SUCCESFULL
YES	ST	BW	LT	CE	LOST POSSESSION
YES	ST	BW	LT	CE	LOST POSSESSION
YES	ST	BW	LT	MO	CONTINUED POSSESSION
YES	ST	BW	RT	MO	CONTINUED POSSESSION
YES	ME	BW	RT	OF	CONTINUED POSSESSION
YES	ST	BW	RT	MD	CONTINUED POSSESSION
YES	ST	BW	RT	MO	CONTINUED POSSESSION
YES	ST	FW	LT	CE	CONTINUED POSSESSION
YES	ME	BW	RT	CE	CONTINUED POSSESSION
YES	ME	BW	LT	MO	CONTINUED POSSESSION
YES	ST	BW	LT	MD	CONTINUED POSSESSION
YES	ST	FW	RT	MO	CONTINUED POSSESSION
YES	ST	BW	RT	OF	CONTINUED POSSESSION
YES	ME	BW	RT	OF	CONTINUED POSSESSION
YES	ST	FW	RT	MO	CONTINUED POSSESSION
YES	ST	BW	LT	DF	LOST POSSESSION
YES	ME	BW	LT	MD	CONTINUED POSSESSION
YES	ME	FW	RT	CE	CONTINUED POSSESSION
YES	ME	BW	RT	MO	CONTINUED POSSESSION
YES	ME	FW	RT	OF	SUCCESFULL
YES	ST	FW	RT	OF	CONTINUED POSSESSION
YES	ST	BW	RT	MO	CONTINUED POSSESSION
YES	ME	BW	RT	MD	CONTINUED POSSESSION
YES	ST	BW	LT	MO	CONTINUED POSSESSION
YES	ME	FW	LT	CE	LOST POSSESSION
YES	ST	BW	RT	CE	CONTINUED POSSESSION
YES	ST	FW	RT	MD	CONTINUED POSSESSION
YES	ST	FW	RT	MD	CONTINUED POSSESSION
YES	ST	BW	RT	MO	CONTINUED POSSESSION
YES	ME	FW	RT	MO	LOST POSSESSION
YES	ST	FW	RT	MD	LOST POSSESSION
YES	ST	FW	RT	DF	LOST POSSESSION
YES	ST	FW	RT	OF	LOST POSSESSION
YES	ST	FW	LT	MO	LOST POSSESSION
YES	ME	BW	RT	CE	CONTINUED POSSESSION
YES	ME	BW	LT	CE	CONTINUED POSSESSION
YES	ME	FW	LT	MO	CONTINUED POSSESSION
YES	ME	FW	LT	CE	LOST POSSESSION
YES	ST	FW	RT	CE	CONTINUED POSSESSION
YES	ME	FW	RT	CE	LOST POSSESSION
YES	ME	BW	LT	MO	SUCCESFULL
YES	ME	FW	LT	DF	CONTINUED POSSESSION
YES	ME	FW	LT	MD	LOST POSSESSION
YES	ME	FW	LT	MO	LOST POSSESSION
YES	ST	BW	LT	MO	CONTINUED POSSESSION
YES	ST	FW	RT	MO	CONTINUED POSSESSION
YES	ME	FW	LT	CE	LOST POSSESSION
YES	ST	FW	RT	CE	LOST POSSESSION

YES	ME	FW	RT	MD	LOST POSSESSION
YES	ST	BW	RT	MO	CONTINUED POSSESSION
YES	ST	FW	LT	CE	CONTINUED POSSESSION
YES	ST	FW	RT	OF	CONTINUED POSSESSION
YES	ST	FW	RT	OF	CONTINUED POSSESSION
YES	ME	FW	LT	MD	LOST POSSESSION
YES	ST	FW	RT	MD	LOST POSSESSION
YES	ME	FW	RT	CE	LOST POSSESSION
YES	ME	BW	LT	MO	CONTINUED POSSESSION
YES	ME	FW	LT	MD	LOST POSSESSION
YES	ST	FW	LT	OF	CONTINUED POSSESSION
YES	ME	FW	LT	MO	LOST POSSESSION
YES	ME	BW	LT	MO	CONTINUED POSSESSION
YES	LG	FW	RT	OF	SUCCESFULL
YES	ST	BW	RT	CE	LOST POSSESSION
YES	ME	BW	RT	DF	LOST POSSESSION
YES	ME	BW	LT	CE	LOST POSSESSION
YES	ME	FW	RT	CE	SUCCESFULL
YES	ST	BW	LT	OF	CONTINUED POSSESSION
YES	ST	FW	RT	OF	CONTINUED POSSESSION
YES	ST	BW	LT	MD	CONTINUED POSSESSION
YES	LG	BW	RT	MD	CONTINUED POSSESSION
YES	ME	BW	LT	CE	CONTINUED POSSESSION
YES	ST	FW	RT	DF	CONTINUED POSSESSION
YES	ST	FW	RT	CE	LOST POSSESSION
YES	ME	BW	LT	CE	CONTINUED POSSESSION
YES	ME	BW	LT	MO	CONTINUED POSSESSION
YES	ST	FW	RT	MO	CONTINUED POSSESSION
YES	ME	FW	RT	OF	CONTINUED POSSESSION
YES	ST	FW	RT	CE	CONTINUED POSSESSION
YES	ME	BW	LT	CE	CONTINUED POSSESSION
YES	ME	BW	RT	MD	LOST POSSESSION
YES	ME	BW	LT	MD	CONTINUED POSSESSION
YES	ST	FW	LT	MD	CONTINUED POSSESSION
YES	ME	BW	LT	MO	CONTINUED POSSESSION
YES	ST	BW	LT	OF	CONTINUED POSSESSION
YES	ST	BW	LT	CE	CONTINUED POSSESSION
YES	ME	BW	RT	MD	CONTINUED POSSESSION
YES	ST	FW	RT	OF	CONTINUED POSSESSION
YES	ST	BW	RT	MD	CONTINUED POSSESSION
YES	ST	FW	LT	MO	LOST POSSESSION
YES	ME	BW	RT	MD	CONTINUED POSSESSION
NO	ME	BW	LT	CE	CONTINUED POSSESSION
YES	ST	BW	RT	MO	SUCCESFULL
NO	ME	BW	RT	MO	CONTINUED POSSESSION
YES	ST	BW	RT	MO	LOST POSSESSION
YES	ME	BW	RT	MO	LOST POSSESSION
YES	ST	FW	RT	MO	LOST POSSESSION
YES	ME	FW	RT	MD	LOST POSSESSION
YES	ST	FW	LT	MO	CONTINUED POSSESSION

YES	ST	FW	LT	MO	LOST POSSESSION
YES	ST	FW	LT	CE	LOST POSSESSION
YES	ST	FW	LT	CE	LOST POSSESSION
YES	ST	FW	LT	MD	CONTINUED POSSESSION
YES	ST	FW	LT	MD	CONTINUED POSSESSION
YES	ST	BW	LT	MO	CONTINUED POSSESSION
YES	ST	FW	LT	MD	LOST POSSESSION
YES	ST	FW	LT	CE	LOST POSSESSION
YES	ST	FW	RT	CE	CONTINUED POSSESSION
YES	ME	FW	LT	CE	SUCCESFULL
YES	ST	FW	RT	MO	LOST POSSESSION
YES	ST	BW	RT	OF	LOST POSSESSION
YES	ST	BW	LT	MO	SUCCESFULL
YES	ME	FW	RT	CE	LOST POSSESSION
YES	ME	BW	RT	CE	CONTINUED POSSESSION
YES	ME	BW	RT	MD	CONTINUED POSSESSION
YES	ST	FW	LT	MO	CONTINUED POSSESSION
YES	ME	BW	RT	CE	CONTINUED POSSESSION
YES	ME	BW	RT	MO	CONTINUED POSSESSION
YES	ST	FW	LT	MO	LOST POSSESSION
YES	ST	BW	LT	CE	CONTINUED POSSESSION
YES	ST	FW	LT	DF	LOST POSSESSION
YES	ST	FW	RT	CE	CONTINUED POSSESSION
YES	ST	BW	RT	CE	CONTINUED POSSESSION
YES	ME	FW	RT	MO	LOST POSSESSION
NO	ME	BW	LT	MD	CONTINUED POSSESSION
YES	ME	FW	LT	DF	CONTINUED POSSESSION
YES	ST	BW	LT	MO	CONTINUED POSSESSION
YES	ME	BW	LT	MO	CONTINUED POSSESSION
YES	ST	FW	RT	CE	LOST POSSESSION
YES	ST	BW	LT	OF	CONTINUED POSSESSION
YES	ME	FW	RT	MO	LOST POSSESSION
YES	ME	FW	LT	CE	LOST POSSESSION
YES	ST	FW	RT	CE	LOST POSSESSION
YES	ST	FW	RT	MD	LOST POSSESSION
YES	ST	FW	LT	MD	CONTINUED POSSESSION
YES	ME	FW	LT	CE	LOST POSSESSION
YES	ME	FW	RT	CE	CONTINUED POSSESSION
YES	ME	FW	RT	DF	CONTINUED POSSESSION
NO	ME	FW	RT	MD	LOST POSSESSION
YES	ME	BW	RT	MD	CONTINUED POSSESSION
YES	ME	FW	RT	MO	CONTINUED POSSESSION
YES	ST	BW	LT	OF	CONTINUED POSSESSION
YES	ME	FW	LT	MO	LOST POSSESSION
YES	ME	BW	RT	CE	CONTINUED POSSESSION
YES	ME	BW	RT	MO	CONTINUED POSSESSION
YES	ME	BW	RT	CE	CONTINUED POSSESSION
NO	ST	BW	RT	MD	LOST POSSESSION
YES	ST	FW	RT	MD	CONTINUED POSSESSION
YES	ME	FW	RT	MO	LOST POSSESSION

YES	ST	BW	RT	CE	LOST POSSESSION
YES	ST	FW	RT	OF	CONTINUED POSSESSION
YES	ST	FW	RT	MD	CONTINUED POSSESSION
YES	ME	FW	LT	MD	LOST POSSESSION
YES	ST	FW	LT	MD	LOST POSSESSION
YES	ST	FW	LT	CE	CONTINUED POSSESSION
YES	ME	FW	RT	OF	LOST POSSESSION
NO	ST	BW	LT	MD	CONTINUED POSSESSION
YES	ME	BW	RT	MO	CONTINUED POSSESSION
YES	ST	BW	LT	MD	UNSUCCESFULL
YES	ST	FW	RT	MO	SUCCESFULL
YES	ST	FW	LT	MO	CONTINUED POSSESSION
YES	ST	BW	LT	MO	LOST POSSESSION
YES	ST	BW	RT	MO	LOST POSSESSION
YES	ST	FW	LT	MD	SUCCESFULL
NO	ME	BW	RT	CE	CONTINUED POSSESSION
YES	ST	FW	LT	DF	LOST POSSESSION
NO	ME	BW	LT	MO	CONTINUED POSSESSION
YES	ST	FW	LT	MO	CONTINUED POSSESSION
YES	ST	FW	LT	MO	LOST POSSESSION
NO	ST	BW	LT	MO	CONTINUED POSSESSION
YES	ME	BW	RT	MD	LOST POSSESSION
YES	ST	FW	RT	OF	SUCCESFULL
NO	ME	FW	RT	CE	CONTINUED POSSESSION
NO	ST	BW	LT	MD	LOST POSSESSION
YES	ST	FW	LT	DF	LOST POSSESSION
YES	ST	FW	LT	CE	CONTINUED POSSESSION
YES	ST	BW	RT	MO	SUCCESFULL
YES	ME	FW	RT	CE	CONTINUED POSSESSION
YES	ME	BW	RT	MO	SUCCESFULL
YES	ST	FW	LT	MD	CONTINUED POSSESSION
YES	ST	BW	LT	MO	CONTINUED POSSESSION
NO	ME	BW	RT	MD	CONTINUED POSSESSION
YES	ST	FW	RT	MO	LOST POSSESSION
YES	ST	BW	LT	MO	CONTINUED POSSESSION
YES	ME	FW	RT	MO	CONTINUED POSSESSION
YES	ST	FW	RT	OF	LOST POSSESSION
YES	ME	FW	RT	OF	LOST POSSESSION
YES	ST	BW	RT	OF	CONTINUED POSSESSION
YES	ST	BW	RT	OF	LOST POSSESSION
YES	ST	BW	LT	OF	CONTINUED POSSESSION
YES	ST	FW	LT	MO	LOST POSSESSION
NO	ME	BW	RT	MO	CONTINUED POSSESSION
YES	ST	FW	LT	MO	CONTINUED POSSESSION
YES	ST	FW	RT	MO	CONTINUED POSSESSION
YES	ST	FW	RT	MO	CONTINUED POSSESSION
YES	ST	BW	RT	CE	CONTINUED POSSESSION
NO	ST	BW	LT	MD	CONTINUED POSSESSION
NO	ST	BW	LT	MD	CONTINUED POSSESSION
YES	ST	FW	LT	CE	CONTINUED POSSESSION

YES	ST	FW	RT	MO	LOST POSSESSION
NO	ME	BW	RT	MO	CONTINUED POSSESSION
YES	LG	BW	LT	MO	CONTINUED POSSESSION
YES	ST	BW	RT	CE	CONTINUED POSSESSION
YES	ST	FW	LT	CE	UNSUCCESFULL
YES	ST	BW	RT	MO	CONTINUED POSSESSION
YES	ST	FW	LT	OF	UNSUCCESFULL
YES	ME	FW	RT	MO	LOST POSSESSION
YES	ST	FW	RT	MO	LOST POSSESSION
YES	ME	FW	RT	OF	LOST POSSESSION
YES	ST	FW	LT	OF	LOST POSSESSION
YES	ME	FW	RT	MD	CONTINUED POSSESSION
YES	ST	BW	LT	OF	CONTINUED POSSESSION
YES	LG	FW	RT	OF	CONTINUED POSSESSION
YES	ST	FW	RT	MO	CONTINUED POSSESSION
YES	ST	FW	LT	MD	CONTINUED POSSESSION
YES	ST	FW	LT	MO	CONTINUED POSSESSION
YES	ST	BW	LT	OF	CONTINUED POSSESSION
YES	ST	FW	RT	CE	CONTINUED POSSESSION
YES	ST	BW	RT	MO	CONTINUED POSSESSION
YES	ST	FW	RT	MD	LOST POSSESSION
NO	ME	BW	LT	MO	CONTINUED POSSESSION
NO	ST	BW	RT	CE	CONTINUED POSSESSION
YES	ST	FW	LT	DF	LOST POSSESSION
NO	ST	BW	RT	CE	CONTINUED POSSESSION
YES	ST	BW	LT	OF	CONTINUED POSSESSION
YES	ST	FW	RT	CE	CONTINUED POSSESSION
YES	ME	FW	LT	OF	CONTINUED POSSESSION
YES	ST	BW	LT	MO	CONTINUED POSSESSION
YES	ME	FW	LT	MO	CONTINUED POSSESSION
YES	ME	FW	RT	MO	LOST POSSESSION
YES	ST	FW	RT	CE	CONTINUED POSSESSION
YES	ST	FW	RT	MO	LOST POSSESSION
YES	ST	FW	LT	MO	LOST POSSESSION
YES	LG	FW	RT	MD	CONTINUED POSSESSION
YES	ME	FW	RT	MD	LOST POSSESSION
YES	ME	BW	RT	CE	CONTINUED POSSESSION
YES	ME	FW	RT	CE	LOST POSSESSION
YES	LG	FW	LT	MO	CONTINUED POSSESSION
YES	LG	FW	LT	CE	LOST POSSESSION
YES	ME	BW	RT	CE	CONTINUED POSSESSION
YES	ME	FW	RT	MD	CONTINUED POSSESSION
YES	ME	BW	LT	CE	LOST POSSESSION
NO	LG	BW	LT	DF	CONTINUED POSSESSION
YES	ME	BW	LT	CE	CONTINUED POSSESSION
YES	ST	BW	LT	MD	CONTINUED POSSESSION
YES	ME	BW	RT	MO	CONTINUED POSSESSION
YES	ME	BW	LT	MO	CONTINUED POSSESSION
YES	ME	BW	RT	OF	LOST POSSESSION
YES	LG	FW	LT	CE	LOST POSSESSION

YES	ME	BW	RT	MD	CONTINUED POSSESSION
NO	ST	BW	LT	MD	CONTINUED POSSESSION
NO	ST	BW	RT	DF	CONTINUED POSSESSION
YES	ME	FW	RT	MO	SUCCESFULL
YES	ST	FW	RT	DF	LOST POSSESSION
YES	ST	FW	RT	MD	LOST POSSESSION
YES	ST	FW	LT	MD	CONTINUED POSSESSION
YES	ST	FW	LT	MD	LOST POSSESSION
YES	ST	FW	RT	MD	LOST POSSESSION
YES	ME	FW	LT	MO	LOST POSSESSION
YES	ST	BW	LT	CE	CONTINUED POSSESSION
YES	ME	FW	LT	MO	LOST POSSESSION
YES	ME	FW	LT	CE	LOST POSSESSION
YES	ST	BW	RT	OF	SUCCESFULL
YES	ST	FW	RT	DF	LOST POSSESSION
YES	ME	FW	LT	MD	LOST POSSESSION
YES	ST	FW	LT	CE	LOST POSSESSION
YES	ST	FW	LT	MD	LOST POSSESSION
YES	ST	BW	LT	MO	CONTINUED POSSESSION
YES	ST	FW	RT	OF	SUCCESFULL
NO	ME	BW	LT	MO	SUCCESFULL
YES	ST	FW	RT	OF	CONTINUED POSSESSION
YES	ST	FW	RT	MD	CONTINUED POSSESSION
YES	ST	FW	RT	CE	SUCCESFULL
YES	ST	BW	RT	MO	LOST POSSESSION
YES	ST	FW	RT	MO	CONTINUED POSSESSION
YES	ST	FW	RT	OF	CONTINUED POSSESSION
YES	ST	BW	LT	MD	LOST POSSESSION
YES	ME	FW	LT	CE	CONTINUED POSSESSION
YES	ST	FW	RT	DF	LOST POSSESSION
YES	ST	BW	RT	MO	CONTINUED POSSESSION
YES	ST	BW	LT	MD	CONTINUED POSSESSION
YES	ME	FW	LT	MO	CONTINUED POSSESSION
YES	ME	FW	LT	MO	SUCCESFULL
YES	ST	FW	LT	MO	LOST POSSESSION
YES	ST	FW	RT	MO	LOST POSSESSION
YES	ST	FW	LT	CE	SUCCESFULL
YES	ST	BW	RT	OF	SUCCESFULL
YES	ST	FW	RT	DF	SUCCESFULL
YES	ME	FW	RT	CE	LOST POSSESSION
YES	ST	FW	LT	MD	LOST POSSESSION
NO	LG	BW	LT	MO	CONTINUED POSSESSION
YES	ST	BW	RT	MD	LOST POSSESSION
YES	ME	FW	LT	MO	LOST POSSESSION
YES	ST	FW	RT	OF	CONTINUED POSSESSION
YES	ST	BW	LT	MD	UNSUCCESFULL
NO	ST	BW	LT	MD	CONTINUED POSSESSION
YES	ST	FW	LT	OF	SUCCESFULL
YES	ME	FW	RT	MD	CONTINUED POSSESSION
YES	ME	BW	RT	CE	CONTINUED POSSESSION

YES	ME	FW	RT	CE	CONTINUED POSSESSION
YES	ME	FW	RT	MO	LOST POSSESSION
YES	ME	FW	LT	OF	CONTINUED POSSESSION
YES	ME	FW	LT	MO	CONTINUED POSSESSION
YES	ST	FW	RT	CE	LOST POSSESSION
YES	ST	BW	LT	CE	LOST POSSESSION
YES	ST	BW	LT	CE	LOST POSSESSION
YES	ME	FW	RT	MD	LOST POSSESSION
YES	ME	FW	RT	MO	CONTINUED POSSESSION
YES	ME	FW	RT	MO	LOST POSSESSION
YES	ST	BW	LT	MD	LOST POSSESSION
YES	ME	FW	RT	MD	CONTINUED POSSESSION
YES	ME	FW	RT	CE	UNSUCCESFULL
YES	ME	FW	RT	CE	CONTINUED POSSESSION
YES	ST	FW	RT	CE	LOST POSSESSION
YES	ME	FW	RT	MD	LOST POSSESSION
YES	ST	FW	RT	MO	LOST POSSESSION
YES	ME	FW	RT	MD	LOST POSSESSION
YES	ME	FW	RT	MD	LOST POSSESSION
YES	ME	FW	RT	CE	LOST POSSESSION
YES	ST	FW	LT	MO	LOST POSSESSION
YES	ST	FW	RT	MD	CONTINUED POSSESSION
YES	ST	BW	LT	MO	LOST POSSESSION
YES	ST	BW	RT	MD	CONTINUED POSSESSION
YES	ST	BW	LT	CE	LOST POSSESSION
YES	ME	BW	RT	CE	CONTINUED POSSESSION
YES	ST	FW	LT	OF	LOST POSSESSION
YES	ST	FW	LT	MD	LOST POSSESSION
YES	ST	FW	LT	MO	CONTINUED POSSESSION
YES	ME	BW	LT	CE	CONTINUED POSSESSION
YES	ME	FW	RT	OF	LOST POSSESSION
YES	ME	FW	RT	MD	LOST POSSESSION
YES	ST	FW	LT	MO	CONTINUED POSSESSION
YES	ST	FW	LT	MO	CONTINUED POSSESSION
YES	ST	BW	LT	MD	SUCCESFULL
YES	ME	BW	LT	OF	SUCCESFULL
YES	LG	FW	LT	CE	CONTINUED POSSESSION
YES	ME	FW	RT	MO	LOST POSSESSION
YES	ME	FW	RT	CE	LOST POSSESSION
YES	ME	FW	LT	MO	LOST POSSESSION
YES	ME	FW	LT	MO	LOST POSSESSION
YES	ME	FW	RT	DF	LOST POSSESSION
YES	ST	FW	RT	MO	CONTINUED POSSESSION
YES	LG	FW	RT	CE	CONTINUED POSSESSION
YES	LG	FW	RT	MO	LOST POSSESSION
YES	ME	FW	RT	MD	CONTINUED POSSESSION
YES	LG	FW	RT	MO	LOST POSSESSION
YES	ST	BW	RT	MO	CONTINUED POSSESSION
YES	LG	FW	RT	MO	LOST POSSESSION
NO	LG	BW	RT	MD	CONTINUED POSSESSION

YES	ST	FW	LT	CE	CONTINUED POSSESSION
YES	ST	BW	RT	DF	CONTINUED POSSESSION
YES	LG	FW	RT	CE	CONTINUED POSSESSION
YES	ME	FW	RT	MO	CONTINUED POSSESSION
YES	ST	BW	RT	OF	LOST POSSESSION
YES	ME	FW	RT	CE	LOST POSSESSION
YES	ME	BW	LT	OF	CONTINUED POSSESSION
YES	ME	FW	LT	MO	SUCCESFULL
YES	ME	FW	RT	MD	UNSUCCESFULL
YES	ME	BW	LT	MD	CONTINUED POSSESSION
YES	ST	BW	RT	OF	LOST POSSESSION
YES	ME	FW	RT	MO	LOST POSSESSION
YES	ST	FW	LT	MO	CONTINUED POSSESSION
YES	LG	FW	RT	OF	LOST POSSESSION
YES	LG	FW	RT	OF	LOST POSSESSION
YES	LG	FW	RT	OF	LOST POSSESSION
YES	ME	BW	RT	MO	CONTINUED POSSESSION
YES	ST	FW	RT	CE	LOST POSSESSION
YES	ME	FW	RT	CE	CONTINUED POSSESSION
YES	LG	FW	RT	MD	LOST POSSESSION
YES	ME	FW	RT	MO	LOST POSSESSION
YES	ME	FW	LT	CE	CONTINUED POSSESSION
YES	ME	BW	LT	CE	CONTINUED POSSESSION
YES	ME	BW	LT	OF	CONTINUED POSSESSION
YES	ME	BW	RT	OF	CONTINUED POSSESSION
YES	ME	FW	RT	MO	LOST POSSESSION
YES	ST	FW	RT	MD	LOST POSSESSION
YES	ME	BW	LT	CE	CONTINUED POSSESSION
YES	ME	BW	LT	CE	CONTINUED POSSESSION
YES	LG	FW	RT	DF	LOST POSSESSION
YES	ME	FW	RT	MD	LOST POSSESSION
YES	ST	BW	RT	OF	LOST POSSESSION
YES	ST	FW	LT	MD	LOST POSSESSION
YES	ME	FW	RT	MD	LOST POSSESSION
YES	ME	BW	RT	CE	CONTINUED POSSESSION
YES	ST	FW	RT	MO	LOST POSSESSION
YES	ST	FW	LT	MD	CONTINUED POSSESSION
YES	ME	FW	LT	MD	CONTINUED POSSESSION
YES	ME	FW	LT	DF	UNSUCCESFULL
YES	ST	FW	LT	MD	CONTINUED POSSESSION
YES	ME	FW	LT	MD	LOST POSSESSION
YES	ME	FW	LT	MD	LOST POSSESSION
YES	ME	FW	RT	DF	LOST POSSESSION
YES	ST	BW	RT	MD	LOST POSSESSION
YES	ME	FW	RT	MO	LOST POSSESSION
YES	ST	BW	RT	OF	CONTINUED POSSESSION
YES	ST	FW	LT	MD	LOST POSSESSION
YES	ME	FW	RT	CE	LOST POSSESSION
YES	ME	FW	LT	MO	LOST POSSESSION
YES	ME	FW	RT	CE	UNSUCCESFULL

YES	LG	FW	LT	MD	CONTINUED POSSESSION
YES	ME	FW	LT	CE	LOST POSSESSION
YES	ST	FW	LT	OF	SUCCESFULL
YES	ST	FW	RT	OF	CONTINUED POSSESSION
YES	ST	FW	LT	CE	LOST POSSESSION
YES	ST	FW	LT	MD	LOST POSSESSION
YES	ST	FW	RT	CE	LOST POSSESSION
YES	ST	BW	LT	CE	CONTINUED POSSESSION
YES	ST	BW	LT	CE	CONTINUED POSSESSION
YES	ME	FW	LT	MD	LOST POSSESSION
YES	ME	FW	RT	MD	CONTINUED POSSESSION
YES	ST	FW	LT	MD	LOST POSSESSION
YES	ST	BW	RT	MO	LOST POSSESSION
YES	ME	BW	RT	CE	LOST POSSESSION
YES	ST	FW	RT	DF	UNSUCCESFULL
YES	ST	BW	RT	MD	CONTINUED POSSESSION
YES	ST	FW	RT	DF	LOST POSSESSION
YES	ME	FW	RT	DF	UNSUCCESFULL
YES	ST	FW	LT	MO	LOST POSSESSION
YES	ST	FW	RT	CE	CONTINUED POSSESSION
YES	ST	FW	LT	OF	CONTINUED POSSESSION
YES	ME	FW	LT	CE	LOST POSSESSION
YES	ME	FW	RT	MO	LOST POSSESSION
YES	ST	FW	LT	OF	LOST POSSESSION
YES	ME	FW	RT	MD	LOST POSSESSION
YES	ST	FW	RT	CE	LOST POSSESSION
YES	ST	FW	RT	CE	CONTINUED POSSESSION
YES	ST	BW	RT	MD	CONTINUED POSSESSION
YES	LG	FW	RT	MD	LOST POSSESSION
YES	LG	FW	RT	DF	LOST POSSESSION
YES	ST	FW	RT	MO	LOST POSSESSION
YES	ST	BW	LT	MO	CONTINUED POSSESSION
YES	ME	BW	LT	CE	CONTINUED POSSESSION
YES	ST	FW	LT	MO	LOST POSSESSION
YES	ME	FW	LT	MO	CONTINUED POSSESSION
YES	ST	FW	LT	CE	CONTINUED POSSESSION
YES	ST	BW	RT	OF	CONTINUED POSSESSION
YES	ST	BW	RT	OF	SUCCESFULL
YES	ME	BW	RT	CE	CONTINUED POSSESSION
YES	ME	BW	LT	CE	CONTINUED POSSESSION
YES	ST	BW	LT	MO	CONTINUED POSSESSION
YES	ST	BW	LT	CE	CONTINUED POSSESSION
NO	ME	BW	LT	MD	CONTINUED POSSESSION
YES	ME	BW	LT	MO	CONTINUED POSSESSION
YES	ST	BW	LT	MO	SUCCESFULL
NO	ME	BW	RT	MD	CONTINUED POSSESSION
YES	ME	BW	LT	CE	CONTINUED POSSESSION
YES	ME	BW	RT	OF	LOST POSSESSION
YES	ME	FW	LT	MD	LOST POSSESSION
YES	LG	FW	LT	MD	CONTINUED POSSESSION

YES	LG	FW	RT	DF	LOST POSSESSION
YES	ME	FW	LT	MD	CONTINUED POSSESSION
NO	LG	BW	LT	MD	CONTINUED POSSESSION
YES	ST	BW	RT	MD	LOST POSSESSION
YES	ST	BW	RT	CE	LOST POSSESSION
YES	ST	BW	LT	MO	SUCCESFULL
YES	ME	BW	RT	MD	CONTINUED POSSESSION
YES	ME	BW	RT	CE	CONTINUED POSSESSION
YES	ME	BW	RT	CE	LOST POSSESSION
YES	ST	BW	LT	CE	CONTINUED POSSESSION
YES	ME	BW	RT	CE	CONTINUED POSSESSION
YES	LG	FW	LT	CE	LOST POSSESSION
YES	ST	FW	LT	MO	LOST POSSESSION
YES	ME	FW	RT	CE	LOST POSSESSION
YES	ME	FW	RT	MD	LOST POSSESSION
YES	ME	FW	LT	MD	SUCCESFULL
YES	ME	FW	LT	MO	SUCCESFULL
YES	ME	FW	RT	DF	LOST POSSESSION
YES	ME	FW	RT	DF	CONTINUED POSSESSION
YES	ME	FW	RT	CE	LOST POSSESSION
YES	ST	FW	LT	CE	LOST POSSESSION
YES	LG	FW	LT	DF	LOST POSSESSION
YES	ME	FW	RT	MD	LOST POSSESSION
YES	ME	FW	RT	MO	LOST POSSESSION
YES	LG	FW	RT	DF	LOST POSSESSION
YES	ME	BW	RT	CE	CONTINUED POSSESSION
YES	ME	BW	LT	MO	CONTINUED POSSESSION
YES	LG	FW	LT	MD	LOST POSSESSION
YES	ST	BW	LT	MD	CONTINUED POSSESSION
YES	ST	BW	RT	MD	CONTINUED POSSESSION
YES	ST	BW	RT	CE	CONTINUED POSSESSION
YES	ME	BW	LT	MD	CONTINUED POSSESSION
YES	ST	FW	LT	DF	CONTINUED POSSESSION
YES	ST	BW	RT	OF	SUCCESFULL
YES	ST	FW	LT	MD	LOST POSSESSION
YES	ME	BW	RT	MD	CONTINUED POSSESSION
YES	ST	FW	RT	MO	CONTINUED POSSESSION
YES	ME	FW	RT	MD	CONTINUED POSSESSION
YES	ST	FW	RT	CE	CONTINUED POSSESSION
YES	ST	FW	RT	MO	LOST POSSESSION
YES	ST	BW	RT	OF	CONTINUED POSSESSION
YES	ST	FW	RT	CE	CONTINUED POSSESSION
YES	ST	FW	LT	MO	LOST POSSESSION
YES	ST	FW	LT	MD	CONTINUED POSSESSION
YES	ST	FW	LT	MD	LOST POSSESSION
YES	ST	BW	RT	MD	CONTINUED POSSESSION
YES	ME	FW	LT	MD	LOST POSSESSION
YES	ST	FW	RT	OF	CONTINUED POSSESSION
YES	ME	FW	RT	OF	LOST POSSESSION
YES	ST	FW	RT	MO	LOST POSSESSION

YES	ST	FW	LT	CE	LOST POSSESSION
YES	ST	FW	LT	CE	LOST POSSESSION
YES	ST	FW	RT	DF	CONTINUED POSSESSION
YES	ME	FW	RT	MD	SUCCESFULL
YES	ST	FW	RT	OF	CONTINUED POSSESSION
YES	ST	BW	RT	MD	CONTINUED POSSESSION
YES	ME	BW	RT	CE	CONTINUED POSSESSION
YES	ST	BW	RT	MO	CONTINUED POSSESSION
YES	ST	FW	LT	CE	LOST POSSESSION
YES	ST	BW	RT	OF	CONTINUED POSSESSION
YES	ST	BW	RT	MO	LOST POSSESSION
YES	ST	BW	LT	CE	CONTINUED POSSESSION
NO	ME	BW	LT	CE	CONTINUED POSSESSION
YES	ME	BW	LT	CE	CONTINUED POSSESSION
YES	ME	BW	RT	MD	LOST POSSESSION
YES	ME	BW	RT	CE	CONTINUED POSSESSION
YES	ME	BW	RT	MD	LOST POSSESSION
YES	ME	BW	RT	MO	CONTINUED POSSESSION
NO	ME	BW	LT	CE	CONTINUED POSSESSION
NO	ME	BW	LT	MO	CONTINUED POSSESSION
NO	ME	BW	LT	MD	CONTINUED POSSESSION
YES	ME	BW	LT	MD	UNSUCCESFULL
YES	ME	FW	LT	DF	LOST POSSESSION
YES	ME	FW	LT	MD	CONTINUED POSSESSION
YES	LG	BW	RT	MO	CONTINUED POSSESSION
YES	ME	BW	RT	MO	CONTINUED POSSESSION
YES	ME	BW	RT	MO	LOST POSSESSION
YES	LG	BW	RT	OF	LOST POSSESSION
YES	ME	BW	RT	CE	CONTINUED POSSESSION
YES	LG	BW	RT	OF	CONTINUED POSSESSION
YES	ST	BW	RT	MO	SUCCESFULL
YES	ME	FW	LT	CE	LOST POSSESSION
YES	ME	FW	RT	CE	LOST POSSESSION
YES	ST	FW	LT	MD	CONTINUED POSSESSION
YES	ST	BW	RT	MO	CONTINUED POSSESSION
YES	ST	FW	LT	OF	CONTINUED POSSESSION
YES	ST	BW	LT	CE	CONTINUED POSSESSION
YES	ST	BW	RT	MD	LOST POSSESSION
YES	ME	FW	RT	CE	CONTINUED POSSESSION
YES	ST	FW	RT	OF	CONTINUED POSSESSION
YES	ST	FW	RT	DF	LOST POSSESSION
YES	ST	BW	LT	MO	CONTINUED POSSESSION
YES	ST	BW	RT	OF	LOST POSSESSION
YES	ST	BW	RT	CE	LOST POSSESSION
NO	ST	BW	LT	MD	CONTINUED POSSESSION
NO	ST	BW	LT	MD	CONTINUED POSSESSION
YES	ST	FW	RT	OF	SUCCESFULL
YES	ST	FW	LT	MD	LOST POSSESSION
NO	ME	BW	RT	MO	CONTINUED POSSESSION
YES	ST	FW	RT	MO	LOST POSSESSION

YES	ST	FW	LT	MO	LOST POSSESSION
NO	ME	BW	RT	CE	SUCCESFULL
YES	ST	FW	LT	OF	SUCCESFULL
YES	ST	FW	LT	MO	CONTINUED POSSESSION
YES	ST	FW	LT	MO	CONTINUED POSSESSION
YES	ST	FW	LT	MO	CONTINUED POSSESSION
YES	ST	FW	LT	MO	LOST POSSESSION
YES	ST	FW	LT	MO	CONTINUED POSSESSION
YES	ST	FW	LT	MO	LOST POSSESSION
YES	ST	FW	LT	MO	LOST POSSESSION
YES	ST	FW	RT	MO	CONTINUED POSSESSION
YES	ST	FW	RT	MO	LOST POSSESSION
YES	ST	FW	LT	MD	CONTINUED POSSESSION
YES	ST	FW	RT	OF	LOST POSSESSION
NO	ME	BW	RT	CE	CONTINUED POSSESSION
NO	ME	BW	RT	CE	CONTINUED POSSESSION
YES	LG	BW	RT	MO	CONTINUED POSSESSION
YES	LG	BW	LT	CE	CONTINUED POSSESSION
YES	ST	BW	RT	OF	LOST POSSESSION
YES	ME	BW	LT	MO	CONTINUED POSSESSION
YES	ME	BW	RT	OF	LOST POSSESSION
YES	ST	BW	LT	DF	CONTINUED POSSESSION
YES	ST	BW	LT	MO	LOST POSSESSION
YES	ME	BW	LT	OF	SUCCESFULL
YES	ST	FW	LT	CE	LOST POSSESSION
YES	ST	FW	LT	MD	LOST POSSESSION
YES	LG	FW	RT	MD	LOST POSSESSION
YES	ME	BW	RT	MD	CONTINUED POSSESSION
YES	ME	FW	LT	MO	CONTINUED POSSESSION
YES	ME	FW	RT	CE	LOST POSSESSION
YES	ME	FW	RT	DF	LOST POSSESSION
YES	ME	FW	RT	MO	LOST POSSESSION
YES	ME	BW	LT	CE	CONTINUED POSSESSION
YES	ST	FW	LT	MD	LOST POSSESSION
YES	ST	FW	LT	OF	CONTINUED POSSESSION
YES	ST	BW	LT	OF	CONTINUED POSSESSION
YES	ST	FW	LT	MO	LOST POSSESSION
YES	ST	FW	RT	CE	LOST POSSESSION
YES	ST	FW	RT	MO	LOST POSSESSION
YES	ME	BW	RT	CE	CONTINUED POSSESSION
YES	ST	FW	RT	MD	LOST POSSESSION
YES	ME	FW	LT	DF	LOST POSSESSION
YES	ST	BW	RT	MO	CONTINUED POSSESSION
YES	ST	FW	LT	CE	CONTINUED POSSESSION
YES	ME	FW	RT	MD	LOST POSSESSION
YES	ME	FW	LT	MD	LOST POSSESSION
YES	ME	FW	LT	MO	LOST POSSESSION
YES	ME	BW	LT	MD	CONTINUED POSSESSION
YES	ME	BW	RT	CE	CONTINUED POSSESSION
YES	ST	BW	RT	CE	UNSUCCESFULL

YES	ST	FW	LT	CE	LOST POSSESSION
YES	ST	BW	LT	OF	LOST POSSESSION
YES	ME	BW	RT	MO	CONTINUED POSSESSION
YES	ME	BW	LT	MO	CONTINUED POSSESSION
YES	ST	BW	LT	MD	CONTINUED POSSESSION
YES	ME	FW	RT	MD	LOST POSSESSION
YES	ST	FW	RT	MD	CONTINUED POSSESSION
YES	ST	FW	LT	MD	LOST POSSESSION
YES	ST	BW	RT	OF	CONTINUED POSSESSION
YES	ME	FW	LT	MD	LOST POSSESSION
YES	ST	FW	LT	MO	LOST POSSESSION
YES	LG	FW	RT	MO	LOST POSSESSION
YES	LG	FW	LT	OF	LOST POSSESSION
YES	ME	FW	LT	MD	CONTINUED POSSESSION
YES	ME	FW	LT	MO	LOST POSSESSION
YES	ME	BW	LT	MD	CONTINUED POSSESSION
YES	LG	BW	RT	MO	LOST POSSESSION
YES	ME	FW	LT	CE	LOST POSSESSION
YES	ME	FW	LT	OF	SUCCESFULL
YES	ME	FW	LT	CE	CONTINUED POSSESSION
YES	ST	FW	RT	CE	LOST POSSESSION
YES	ST	FW	RT	MD	CONTINUED POSSESSION
YES	ST	FW	RT	MD	UNSUCCESFULL
YES	ST	BW	RT	CE	LOST POSSESSION
YES	ST	FW	RT	CE	UNSUCCESFULL
YES	ME	BW	RT	CE	UNSUCCESFULL
YES	ME	FW	RT	CE	LOST POSSESSION
YES	ME	FW	RT	OF	LOST POSSESSION
YES	ST	BW	LT	MO	CONTINUED POSSESSION
YES	ST	FW	LT	MO	LOST POSSESSION
YES	ME	FW	RT	CE	SUCCESFULL
YES	ME	BW	RT	CE	CONTINUED POSSESSION
YES	LG	FW	LT	OF	LOST POSSESSION
YES	ME	FW	LT	MD	CONTINUED POSSESSION
YES	LG	FW	LT	OF	CONTINUED POSSESSION
YES	LG	FW	LT	OF	UNSUCCESFULL
YES	ST	FW	LT	CE	CONTINUED POSSESSION
YES	LG	FW	LT	MO	SUCCESFULL
YES	LG	BW	RT	CE	CONTINUED POSSESSION
YES	LG	BW	RT	MO	LOST POSSESSION
YES	ME	BW	LT	MD	LOST POSSESSION
YES	ME	FW	RT	MD	CONTINUED POSSESSION
YES	LG	FW	RT	MO	LOST POSSESSION
YES	ST	BW	LT	MD	CONTINUED POSSESSION
YES	ME	FW	RT	MD	SUCCESFULL
YES	ST	FW	LT	MO	LOST POSSESSION
YES	ME	FW	RT	MO	LOST POSSESSION
YES	ME	BW	RT	MO	CONTINUED POSSESSION
YES	ME	BW	RT	MD	CONTINUED POSSESSION
YES	ME	BW	RT	MO	CONTINUED POSSESSION

YES	ME	FW	LT	MO	LOST POSSESSION
YES	ST	FW	RT	CE	CONTINUED POSSESSION
YES	LG	FW	RT	MO	LOST POSSESSION
YES	ME	BW	LT	MD	LOST POSSESSION
YES	ME	BW	LT	CE	CONTINUED POSSESSION
YES	ST	FW	LT	MO	CONTINUED POSSESSION
YES	ST	BW	LT	MO	CONTINUED POSSESSION
YES	ST	BW	LT	MD	CONTINUED POSSESSION
YES	ST	FW	LT	OF	LOST POSSESSION
YES	ME	FW	LT	MD	CONTINUED POSSESSION
YES	ME	FW	LT	DF	LOST POSSESSION
YES	ST	FW	LT	DF	LOST POSSESSION
YES	ME	BW	LT	MO	CONTINUED POSSESSION
YES	ST	BW	LT	CE	SUCCESFULL
YES	ME	FW	LT	OF	LOST POSSESSION
YES	ME	FW	LT	MD	UNSUCCESFULL
YES	ST	BW	LT	MO	CONTINUED POSSESSION
NO	ST	BW	RT	CE	CONTINUED POSSESSION
YES	ST	FW	RT	OF	CONTINUED POSSESSION
YES	LG	FW	LT	MD	LOST POSSESSION
YES	ME	BW	LT	CE	CONTINUED POSSESSION
YES	LG	FW	RT	CE	CONTINUED POSSESSION
YES	ME	FW	RT	MO	CONTINUED POSSESSION
YES	LG	FW	LT	CE	SUCCESFULL
YES	ME	FW	LT	MD	SUCCESFULL
YES	ME	FW	RT	DF	LOST POSSESSION
YES	ME	BW	RT	MD	CONTINUED POSSESSION
YES	ST	BW	LT	MO	CONTINUED POSSESSION
YES	ME	BW	RT	MD	LOST POSSESSION
NO	ST	BW	LT	MD	CONTINUED POSSESSION
YES	ST	BW	LT	OF	CONTINUED POSSESSION
YES	ST	FW	LT	OF	CONTINUED POSSESSION
YES	ST	BW	LT	MO	LOST POSSESSION
YES	LG	BW	RT	MO	CONTINUED POSSESSION
YES	ME	FW	LT	MO	SUCCESFULL
YES	ME	BW	RT	CE	CONTINUED POSSESSION
YES	ST	BW	LT	CE	CONTINUED POSSESSION
YES	ST	BW	LT	OF	LOST POSSESSION
YES	ME	FW	RT	MO	LOST POSSESSION
YES	ME	BW	LT	CE	CONTINUED POSSESSION
YES	ME	FW	RT	DF	LOST POSSESSION
YES	ME	BW	RT	MO	CONTINUED POSSESSION
YES	ST	BW	LT	MO	CONTINUED POSSESSION
YES	ST	FW	RT	MO	CONTINUED POSSESSION
YES	ST	BW	LT	MO	CONTINUED POSSESSION
YES	ME	FW	LT	MO	LOST POSSESSION
NO	ME	FW	LT	DF	LOST POSSESSION
YES	ST	BW	LT	MO	CONTINUED POSSESSION
YES	ST	FW	RT	CE	SUCCESFULL
YES	ST	BW	LT	DF	LOST POSSESSION

YES	ME	FW	LT	MD	LOST POSSESSION
YES	ME	FW	RT	MD	CONTINUED POSSESSION
YES	ME	BW	RT	MO	CONTINUED POSSESSION
YES	ME	BW	LT	MO	CONTINUED POSSESSION
YES	LG	BW	LT	MO	CONTINUED POSSESSION
YES	ME	FW	LT	OF	LOST POSSESSION
NO	ME	BW	RT	MD	CONTINUED POSSESSION
YES	ME	FW	RT	MD	LOST POSSESSION
YES	ME	FW	LT	MO	LOST POSSESSION
YES	LG	BW	LT	OF	LOST POSSESSION
YES	ME	FW	LT	MD	CONTINUED POSSESSION
YES	ST	FW	RT	CE	LOST POSSESSION
YES	ME	FW	LT	DF	CONTINUED POSSESSION
NO	ST	BW	RT	MO	CONTINUED POSSESSION
YES	ST	FW	LT	MD	LOST POSSESSION
YES	ST	FW	LT	CE	CONTINUED POSSESSION
YES	ST	BW	LT	OF	CONTINUED POSSESSION
NO	ST	BW	LT	CE	CONTINUED POSSESSION
YES	ST	FW	LT	MO	CONTINUED POSSESSION
YES	ST	BW	LT	CE	CONTINUED POSSESSION
YES	ST	FW	LT	MO	CONTINUED POSSESSION
YES	ST	BW	RT	MO	CONTINUED POSSESSION
YES	ST	BW	LT	MO	CONTINUED POSSESSION
YES	ST	BW	RT	OF	CONTINUED POSSESSION
YES	ST	FW	RT	OF	SUCCESFULL
YES	ME	FW	LT	OF	SUCCESFULL
NO	ST	BW	LT	MO	CONTINUED POSSESSION
NO	ST	BW	LT	MO	LOST POSSESSION
NO	ME	BW	LT	CE	CONTINUED POSSESSION
NO	ST	BW	LT	MO	CONTINUED POSSESSION
NO	ST	FW	LT	MD	CONTINUED POSSESSION
YES	ST	FW	LT	MO	LOST POSSESSION
YES	ST	BW	LT	MO	CONTINUED POSSESSION
YES	ST	BW	LT	MO	SUCCESFULL
YES	ST	FW	RT	MD	CONTINUED POSSESSION
YES	ME	FW	RT	MD	CONTINUED POSSESSION
YES	ME	BW	RT	CE	CONTINUED POSSESSION
YES	ST	BW	RT	OF	LOST POSSESSION
YES	ST	FW	RT	CE	CONTINUED POSSESSION
NO	ST	BW	RT	OF	SUCCESFULL
YES	ST	BW	RT	OF	CONTINUED POSSESSION
YES	ST	FW	LT	CE	LOST POSSESSION
YES	ST	FW	RT	CE	CONTINUED POSSESSION
YES	ST	FW	LT	MO	CONTINUED POSSESSION
YES	ST	FW	LT	MD	LOST POSSESSION
YES	ST	FW	RT	MO	SUCCESFULL
YES	ST	BW	LT	OF	CONTINUED POSSESSION
YES	ST	FW	LT	MO	CONTINUED POSSESSION
YES	ST	FW	RT	MO	CONTINUED POSSESSION
YES	ST	FW	RT	CE	SUCCESFULL

YES	ME	FW	LT	MD	LOST POSSESSION
YES	ME	FW	RT	MD	LOST POSSESSION
YES	ST	FW	RT	MO	CONTINUED POSSESSION
YES	ME	FW	LT	MD	UNSUCCESFULL
NO	ME	BW	RT	CE	CONTINUED POSSESSION
YES	ST	BW	LT	OF	SUCCESFULL
YES	ST	BW	RT	MO	CONTINUED POSSESSION
NO	ST	BW	RT	CE	CONTINUED POSSESSION
YES	ME	FW	RT	DF	LOST POSSESSION
YES	ST	BW	RT	OF	LOST POSSESSION
YES	ST	FW	RT	MO	CONTINUED POSSESSION
YES	ST	FW	LT	MO	CONTINUED POSSESSION
YES	ST	BW	RT	DF	LOST POSSESSION
YES	ST	FW	LT	MO	LOST POSSESSION
YES	ST	FW	RT	OF	CONTINUED POSSESSION
YES	ST	BW	RT	OF	SUCCESFULL
YES	ME	FW	RT	CE	CONTINUED POSSESSION
YES	ST	BW	RT	OF	CONTINUED POSSESSION
YES	ST	BW	LT	MO	SUCCESFULL
YES	ME	FW	RT	CE	LOST POSSESSION
YES	ST	BW	LT	OF	LOST POSSESSION
YES	ME	FW	RT	CE	LOST POSSESSION
YES	ST	BW	LT	MO	CONTINUED POSSESSION
YES	ST	FW	LT	MO	LOST POSSESSION
YES	ME	FW	LT	DF	UNSUCCESFULL
YES	ST	FW	RT	MO	SUCCESFULL
YES	ME	FW	RT	CE	LOST POSSESSION
YES	ME	FW	RT	DF	LOST POSSESSION
YES	ME	FW	LT	CE	LOST POSSESSION
YES	ME	FW	RT	MD	CONTINUED POSSESSION
YES	ME	FW	RT	MD	SUCCESFULL
YES	ME	FW	RT	MD	LOST POSSESSION
YES	ME	FW	RT	DF	LOST POSSESSION
YES	ME	FW	RT	MD	LOST POSSESSION
YES	LG	FW	RT	CE	LOST POSSESSION
YES	LG	FW	LT	DF	LOST POSSESSION
YES	LG	FW	LT	MO	LOST POSSESSION
YES	ME	FW	RT	CE	LOST POSSESSION
YES	ME	FW	RT	MO	LOST POSSESSION
YES	ME	FW	RT	CE	LOST POSSESSION
YES	LG	FW	RT	MO	LOST POSSESSION
YES	LG	FW	RT	OF	LOST POSSESSION
YES	LG	FW	LT	OF	LOST POSSESSION
YES	LG	FW	LT	OF	LOST POSSESSION
YES	LG	FW	LT	MO	CONTINUED POSSESSION
YES	ME	FW	LT	MO	CONTINUED POSSESSION
YES	ME	FW	RT	CE	LOST POSSESSION
YES	LG	FW	LT	MD	LOST POSSESSION
YES	ME	FW	LT	CE	CONTINUED POSSESSION
YES	ME	FW	LT	CE	CONTINUED POSSESSION

YES	LG	FW	RT	OF	LOST POSSESSION
YES	ME	BW	LT	MO	LOST POSSESSION
YES	LG	FW	LT	MD	LOST POSSESSION
YES	LG	FW	LT	MD	LOST POSSESSION
YES	ME	FW	LT	MD	CONTINUED POSSESSION
YES	ME	FW	RT	CE	CONTINUED POSSESSION
YES	ST	BW	LT	MO	UNSUCCESFULL
YES	LG	BW	RT	CE	CONTINUED POSSESSION
YES	ME	BW	RT	MO	CONTINUED POSSESSION
YES	ME	FW	LT	MD	LOST POSSESSION
YES	ME	FW	RT	MO	LOST POSSESSION
YES	ME	FW	LT	MO	LOST POSSESSION
YES	ME	FW	RT	MO	CONTINUED POSSESSION
YES	ME	BW	RT	MO	CONTINUED POSSESSION
YES	ME	FW	RT	CE	LOST POSSESSION
YES	ME	FW	RT	MD	LOST POSSESSION
YES	ST	FW	RT	MO	CONTINUED POSSESSION
YES	ME	FW	RT	OF	LOST POSSESSION
YES	ST	FW	LT	MD	CONTINUED POSSESSION
YES	ME	BW	RT	CE	CONTINUED POSSESSION
YES	LG	FW	LT	MD	LOST POSSESSION
YES	ST	BW	LT	MD	CONTINUED POSSESSION
YES	ME	BW	RT	MO	LOST POSSESSION
YES	ST	FW	RT	MD	CONTINUED POSSESSION
YES	ME	FW	RT	MO	LOST POSSESSION
YES	ST	BW	LT	MD	CONTINUED POSSESSION
YES	ST	FW	LT	MO	CONTINUED POSSESSION
NO	ST	BW	RT	MD	LOST POSSESSION
YES	ME	BW	LT	MD	LOST POSSESSION
YES	ST	FW	LT	MO	CONTINUED POSSESSION
YES	ME	BW	LT	MO	LOST POSSESSION
YES	ST	FW	LT	DF	CONTINUED POSSESSION
YES	ME	FW	LT	MD	CONTINUED POSSESSION
YES	ME	FW	LT	CE	CONTINUED POSSESSION
YES	ST	BW	LT	MO	CONTINUED POSSESSION
YES	LG	FW	RT	MO	LOST POSSESSION
YES	ST	BW	RT	MO	LOST POSSESSION
YES	LG	FW	RT	CE	LOST POSSESSION
YES	LG	FW	RT	DF	CONTINUED POSSESSION
YES	LG	FW	RT	MD	LOST POSSESSION
YES	ME	FW	RT	MO	LOST POSSESSION
YES	ME	FW	LT	MD	CONTINUED POSSESSION
YES	ME	FW	LT	CE	LOST POSSESSION
YES	ME	FW	RT	CE	LOST POSSESSION
YES	LG	FW	RT	MO	SUCCESFULL
YES	ME	FW	LT	CE	LOST POSSESSION
YES	LG	FW	RT	MO	LOST POSSESSION
YES	LG	FW	RT	CE	CONTINUED POSSESSION
YES	ME	FW	RT	MO	LOST POSSESSION
YES	LG	FW	LT	DF	LOST POSSESSION

YES	ME	FW	LT	CE	UNSUCCESFULL
YES	LG	FW	LT	MD	SUCCESFULL
YES	ST	BW	RT	MO	CONTINUED POSSESSION
YES	ST	FW	LT	CE	CONTINUED POSSESSION
YES	ME	BW	RT	MO	CONTINUED POSSESSION
YES	LG	BW	LT	CE	CONTINUED POSSESSION
YES	LG	BW	RT	CE	CONTINUED POSSESSION
YES	ME	BW	LT	MD	CONTINUED POSSESSION
YES	ST	BW	LT	CE	CONTINUED POSSESSION
YES	ME	BW	RT	CE	CONTINUED POSSESSION
YES	ME	BW	LT	MD	LOST POSSESSION
YES	LG	BW	RT	CE	CONTINUED POSSESSION
YES	LG	FW	RT	OF	CONTINUED POSSESSION
YES	ME	FW	LT	MO	SUCCESFULL
YES	ME	FW	LT	MO	LOST POSSESSION
YES	ST	BW	LT	OF	LOST POSSESSION
YES	ST	BW	RT	OF	CONTINUED POSSESSION
YES	ME	BW	RT	CE	CONTINUED POSSESSION
YES	ME	FW	RT	OF	LOST POSSESSION
YES	ME	BW	RT	OF	LOST POSSESSION
YES	ST	FW	RT	MD	CONTINUED POSSESSION
YES	ME	BW	LT	MD	CONTINUED POSSESSION
YES	LG	FW	RT	MO	LOST POSSESSION
YES	ME	BW	LT	OF	CONTINUED POSSESSION
YES	ME	FW	RT	MD	CONTINUED POSSESSION
YES	LG	FW	RT	CE	LOST POSSESSION
YES	ME	FW	RT	MO	CONTINUED POSSESSION
YES	ME	FW	RT	DF	LOST POSSESSION
YES	ST	BW	RT	MO	CONTINUED POSSESSION
YES	ME	BW	RT	MO	LOST POSSESSION
YES	ME	BW	RT	MD	LOST POSSESSION
YES	ST	BW	LT	CE	LOST POSSESSION
YES	ME	BW	RT	MO	CONTINUED POSSESSION
YES	ST	BW	LT	MO	CONTINUED POSSESSION
YES	LG	FW	RT	CE	CONTINUED POSSESSION
YES	ST	FW	RT	MO	LOST POSSESSION
YES	LG	FW	LT	MD	LOST POSSESSION
YES	ME	FW	RT	CE	CONTINUED POSSESSION
YES	ME	FW	RT	MO	CONTINUED POSSESSION
YES	LG	FW	RT	OF	CONTINUED POSSESSION
YES	LG	FW	LT	MD	LOST POSSESSION
YES	ME	FW	LT	MD	CONTINUED POSSESSION
YES	ST	BW	RT	OF	LOST POSSESSION
YES	ME	FW	LT	CE	CONTINUED POSSESSION
YES	ST	BW	LT	MO	CONTINUED POSSESSION
YES	ST	FW	RT	MO	LOST POSSESSION
YES	LG	FW	RT	MD	CONTINUED POSSESSION
YES	ME	FW	LT	MD	LOST POSSESSION
YES	ME	BW	LT	CE	SUCCESFULL
YES	ME	FW	RT	MO	SUCCESFULL

YES	ME	FW	RT	MD	CONTINUED POSSESSION
YES	ME	BW	LT	MO	SUCCESFULL
YES	LG	FW	RT	MO	CONTINUED POSSESSION
YES	ST	BW	RT	MO	CONTINUED POSSESSION
YES	ME	FW	RT	MO	CONTINUED POSSESSION
YES	ME	FW	RT	MO	CONTINUED POSSESSION
YES	ME	FW	RT	MD	LOST POSSESSION
YES	ME	FW	RT	MD	CONTINUED POSSESSION
YES	ST	FW	LT	OF	CONTINUED POSSESSION
YES	ST	FW	LT	MO	LOST POSSESSION
YES	ME	FW	LT	MO	SUCCESFULL
NO	ME	BW	LT	CE	LOST POSSESSION
YES	ME	FW	LT	MD	CONTINUED POSSESSION
YES	LG	FW	LT	CE	LOST POSSESSION
YES	ME	FW	LT	MD	LOST POSSESSION
YES	ME	FW	RT	CE	LOST POSSESSION
YES	ME	FW	RT	DF	LOST POSSESSION
YES	ME	FW	RT	CE	LOST POSSESSION
YES	ME	BW	RT	CE	CONTINUED POSSESSION
YES	ME	BW	LT	MD	LOST POSSESSION
YES	ME	BW	RT	MD	CONTINUED POSSESSION
YES	ME	FW	RT	CE	LOST POSSESSION
YES	ST	FW	RT	CE	CONTINUED POSSESSION
YES	ST	BW	RT	OF	CONTINUED POSSESSION
YES	LG	BW	RT	MO	CONTINUED POSSESSION
YES	ST	FW	RT	OF	CONTINUED POSSESSION
YES	ST	BW	RT	CE	CONTINUED POSSESSION
YES	ME	BW	LT	MO	CONTINUED POSSESSION
NO	LG	BW	RT	MD	CONTINUED POSSESSION
YES	ST	FW	RT	CE	CONTINUED POSSESSION
YES	LG	BW	LT	MO	CONTINUED POSSESSION
YES	ME	FW	RT	MO	LOST POSSESSION
YES	ST	BW	RT	CE	CONTINUED POSSESSION
NO	ST	BW	RT	MD	CONTINUED POSSESSION
YES	ST	FW	RT	MD	CONTINUED POSSESSION
NO	ST	BW	LT	CE	CONTINUED POSSESSION
NO	LG	BW	RT	MD	CONTINUED POSSESSION
YES	ST	BW	LT	OF	LOST POSSESSION
YES	LG	BW	RT	MO	CONTINUED POSSESSION
NO	ME	BW	LT	MO	CONTINUED POSSESSION
YES	ST	FW	LT	DF	CONTINUED POSSESSION
YES	ST	FW	RT	MO	CONTINUED POSSESSION
YES	ST	FW	RT	OF	CONTINUED POSSESSION
NO	ST	BW	RT	MO	CONTINUED POSSESSION
YES	ST	FW	LT	MO	LOST POSSESSION
YES	ST	FW	RT	MD	CONTINUED POSSESSION
YES	ST	BW	RT	MO	CONTINUED POSSESSION
YES	ST	BW	LT	OF	CONTINUED POSSESSION
YES	ST	FW	LT	DF	CONTINUED POSSESSION
YES	ME	BW	RT	MO	SUCCESFULL

YES	ST	BW	LT	MO	CONTINUED POSSESSION
YES	ST	FW	LT	MO	SUCCESFULL
YES	ST	FW	LT	OF	SUCCESFULL
YES	ST	BW	RT	OF	CONTINUED POSSESSION
YES	ST	FW	LT	MD	LOST POSSESSION
YES	ME	FW	RT	CE	CONTINUED POSSESSION
YES	ST	FW	RT	MO	LOST POSSESSION
YES	ST	FW	LT	MD	CONTINUED POSSESSION
YES	ST	BW	LT	OF	CONTINUED POSSESSION
YES	ST	BW	RT	MO	CONTINUED POSSESSION
YES	ME	BW	LT	MO	CONTINUED POSSESSION
YES	ST	BW	LT	MO	CONTINUED POSSESSION
YES	ME	BW	LT	CE	CONTINUED POSSESSION
YES	ST	FW	RT	DF	CONTINUED POSSESSION
YES	ME	BW	RT	MO	CONTINUED POSSESSION
YES	ME	FW	RT	MD	CONTINUED POSSESSION
YES	ST	FW	RT	MD	LOST POSSESSION
YES	ST	FW	LT	MD	CONTINUED POSSESSION
YES	ST	BW	RT	MO	CONTINUED POSSESSION
YES	ST	FW	RT	CE	LOST POSSESSION
YES	ST	FW	RT	DF	LOST POSSESSION
NO	ST	BW	LT	MO	LOST POSSESSION
YES	ST	FW	RT	MO	CONTINUED POSSESSION
YES	ST	FW	LT	MO	LOST POSSESSION
YES	ST	BW	LT	CE	CONTINUED POSSESSION
YES	ST	FW	RT	MO	SUCCESFULL
YES	ST	BW	RT	DF	CONTINUED POSSESSION
YES	ST	FW	LT	MO	CONTINUED POSSESSION
YES	ST	FW	LT	DF	CONTINUED POSSESSION
YES	ST	FW	LT	MD	CONTINUED POSSESSION
YES	ST	BW	LT	OF	SUCCESFULL
YES	ST	BW	RT	MO	CONTINUED POSSESSION
YES	ME	BW	LT	MD	LOST POSSESSION
YES	ST	FW	LT	MD	CONTINUED POSSESSION
NO	ST	BW	RT	CE	CONTINUED POSSESSION
NO	ME	BW	RT	CE	CONTINUED POSSESSION
NO	ST	BW	LT	MD	CONTINUED POSSESSION
NO	LG	BW	RT	MD	CONTINUED POSSESSION
NO	ST	BW	LT	MO	CONTINUED POSSESSION
YES	ST	FW	LT	CE	CONTINUED POSSESSION
YES	ST	FW	RT	MO	CONTINUED POSSESSION
YES	ST	BW	RT	MO	CONTINUED POSSESSION
NO	ST	BW	RT	MD	CONTINUED POSSESSION
YES	ST	FW	RT	MD	CONTINUED POSSESSION
NO	ST	BW	RT	MO	SUCCESFULL
YES	ST	FW	RT	CE	SUCCESFULL
NO	ST	BW	LT	MD	CONTINUED POSSESSION
NO	ME	BW	LT	MD	CONTINUED POSSESSION
YES	ST	BW	LT	MO	CONTINUED POSSESSION
YES	ST	FW	LT	CE	CONTINUED POSSESSION

YES	ST	FW	RT	MO	CONTINUED POSSESSION
YES	ST	BW	RT	MD	LOST POSSESSION
NO	ME	BW	LT	MD	CONTINUED POSSESSION
NO	ME	BW	RT	CE	CONTINUED POSSESSION
NO	ST	BW	RT	MD	CONTINUED POSSESSION
NO	ME	BW	RT	MO	CONTINUED POSSESSION
YES	ME	BW	LT	MO	CONTINUED POSSESSION
YES	ST	FW	LT	CE	CONTINUED POSSESSION
NO	ST	BW	RT	CE	CONTINUED POSSESSION
YES	ST	BW	RT	MO	SUCCESFULL
YES	ME	FW	RT	DF	LOST POSSESSION
YES	ST	BW	RT	MO	CONTINUED POSSESSION
YES	ME	BW	LT	MO	CONTINUED POSSESSION
YES	ST	BW	RT	OF	CONTINUED POSSESSION
YES	ST	FW	RT	CE	CONTINUED POSSESSION
YES	ST	FW	LT	CE	LOST POSSESSION
YES	LG	FW	RT	MO	CONTINUED POSSESSION
YES	ST	BW	RT	MO	CONTINUED POSSESSION
YES	ST	FW	RT	OF	CONTINUED POSSESSION
YES	ST	BW	RT	OF	CONTINUED POSSESSION
NO	ME	BW	RT	MD	CONTINUED POSSESSION
YES	ME	BW	RT	MO	CONTINUED POSSESSION
YES	ME	BW	LT	MD	CONTINUED POSSESSION
YES	LG	BW	LT	MD	CONTINUED POSSESSION
NO	LG	BW	RT	MD	CONTINUED POSSESSION
YES	ST	FW	LT	MO	CONTINUED POSSESSION
NO	ST	BW	LT	MO	CONTINUED POSSESSION
YES	ST	FW	LT	DF	CONTINUED POSSESSION
YES	ST	FW	RT	MO	CONTINUED POSSESSION
NO	ME	BW	RT	MO	SUCCESFULL
YES	ST	BW	LT	MO	SUCCESFULL
YES	ST	BW	RT	OF	CONTINUED POSSESSION
YES	ST	FW	LT	OF	CONTINUED POSSESSION
NO	LG	BW	RT	MD	CONTINUED POSSESSION
YES	ST	FW	LT	MD	LOST POSSESSION
YES	ST	FW	RT	CE	CONTINUED POSSESSION
YES	ST	FW	RT	MD	CONTINUED POSSESSION
NO	ST	FW	RT	MD	CONTINUED POSSESSION
YES	ST	BW	LT	CE	CONTINUED POSSESSION
YES	ME	FW	RT	DF	LOST POSSESSION
YES	ST	FW	LT	MO	LOST POSSESSION
YES	ST	BW	LT	MD	CONTINUED POSSESSION
YES	LG	BW	RT	MO	CONTINUED POSSESSION
YES	ST	FW	RT	MO	CONTINUED POSSESSION
YES	ST	BW	RT	MO	LOST POSSESSION
YES	ST	FW	RT	OF	CONTINUED POSSESSION
YES	ST	FW	LT	OF	SUCCESFULL
YES	ME	BW	RT	CE	CONTINUED POSSESSION
YES	ME	BW	LT	CE	CONTINUED POSSESSION
YES	ME	BW	RT	MO	CONTINUED POSSESSION

YES	ST	FW	RT	CE	CONTINUED POSSESSION
YES	ME	FW	LT	MD	LOST POSSESSION
YES	ME	BW	RT	MO	CONTINUED POSSESSION
NO	ME	BW	LT	MD	CONTINUED POSSESSION
YES	ME	BW	RT	MO	CONTINUED POSSESSION
YES	ME	FW	RT	MD	LOST POSSESSION
YES	ME	FW	LT	CE	CONTINUED POSSESSION
YES	ME	FW	LT	CE	LOST POSSESSION
YES	ME	FW	LT	MO	LOST POSSESSION
YES	ME	FW	RT	CE	LOST POSSESSION
YES	ST	BW	LT	OF	CONTINUED POSSESSION
NO	LG	BW	LT	DF	CONTINUED POSSESSION
YES	LG	FW	LT	MO	LOST POSSESSION
YES	ME	BW	LT	MD	CONTINUED POSSESSION
YES	ST	BW	RT	OF	LOST POSSESSION
YES	ME	FW	RT	MD	LOST POSSESSION
YES	ME	FW	RT	MO	CONTINUED POSSESSION
YES	ST	BW	RT	MO	LOST POSSESSION
YES	ST	BW	RT	CE	CONTINUED POSSESSION
YES	ME	FW	RT	MO	LOST POSSESSION
YES	ST	FW	RT	MD	CONTINUED POSSESSION
YES	ST	BW	LT	OF	CONTINUED POSSESSION
YES	ST	FW	LT	MO	CONTINUED POSSESSION
YES	ME	FW	RT	MD	CONTINUED POSSESSION
YES	ST	BW	LT	OF	CONTINUED POSSESSION
YES	ST	FW	RT	CE	CONTINUED POSSESSION
YES	ME	FW	LT	MD	LOST POSSESSION
YES	ST	BW	LT	OF	CONTINUED POSSESSION
YES	ME	FW	RT	MO	LOST POSSESSION
YES	ST	FW	RT	MD	LOST POSSESSION
YES	ST	FW	RT	MD	CONTINUED POSSESSION
YES	ST	FW	LT	CE	CONTINUED POSSESSION
YES	ME	FW	RT	MO	CONTINUED POSSESSION
YES	ST	BW	LT	MO	CONTINUED POSSESSION
YES	ST	BW	RT	OF	SUCCESFULL
YES	ST	FW	RT	MO	CONTINUED POSSESSION
YES	ME	FW	RT	MO	CONTINUED POSSESSION
YES	ST	FW	LT	CE	CONTINUED POSSESSION
YES	ME	FW	LT	CE	CONTINUED POSSESSION
YES	ME	FW	LT	MO	LOST POSSESSION
YES	ME	FW	LT	OF	CONTINUED POSSESSION
YES	ST	BW	LT	OF	CONTINUED POSSESSION
YES	ME	FW	LT	OF	CONTINUED POSSESSION
YES	ME	FW	LT	MO	SUCCESFULL
YES	ST	FW	LT	OF	CONTINUED POSSESSION
NO	ME	BW	LT	MD	CONTINUED POSSESSION
YES	ST	FW	LT	MO	SUCCESFULL
YES	ST	FW	LT	MO	CONTINUED POSSESSION
YES	ST	BW	LT	MO	LOST POSSESSION
NO	ME	BW	RT	CE	CONTINUED POSSESSION

YES	ST	FW	LT	CE	CONTINUED POSSESSION
YES	ST	FW	RT	CE	CONTINUED POSSESSION
YES	ST	FW	RT	CE	SUCCESFULL
YES	ST	FW	LT	CE	SUCCESFULL
YES	ST	FW	RT	MD	CONTINUED POSSESSION
YES	ME	FW	RT	MO	LOST POSSESSION
NO	ME	BW	LT	MO	LOST POSSESSION
YES	ST	BW	RT	MO	CONTINUED POSSESSION
YES	ST	FW	RT	CE	CONTINUED POSSESSION
YES	ST	FW	RT	CE	CONTINUED POSSESSION
YES	ST	BW	RT	MO	LOST POSSESSION
YES	ST	BW	RT	OF	LOST POSSESSION
YES	ME	FW	LT	MO	LOST POSSESSION
YES	ME	FW	RT	MD	LOST POSSESSION
YES	ST	BW	LT	DF	CONTINUED POSSESSION
YES	ST	FW	RT	CE	LOST POSSESSION
NO	ME	BW	RT	CE	CONTINUED POSSESSION
YES	ME	FW	LT	DF	LOST POSSESSION
YES	ME	FW	LT	OF	LOST POSSESSION
YES	ME	FW	LT	CE	LOST POSSESSION
NO	LG	BW	LT	MO	SUCCESFULL
YES	ME	FW	RT	MD	LOST POSSESSION
YES	ME	FW	RT	MO	LOST POSSESSION
YES	ST	FW	RT	CE	CONTINUED POSSESSION
YES	ME	FW	LT	MO	LOST POSSESSION
YES	ME	FW	RT	MO	LOST POSSESSION
YES	ME	FW	LT	CE	LOST POSSESSION
YES	ME	FW	LT	CE	CONTINUED POSSESSION
YES	LG	FW	LT	DF	CONTINUED POSSESSION
YES	ME	BW	RT	CE	CONTINUED POSSESSION
YES	ME	FW	LT	OF	CONTINUED POSSESSION
YES	LG	BW	RT	MD	LOST POSSESSION
YES	LG	FW	RT	DF	LOST POSSESSION
YES	ME	FW	RT	MO	CONTINUED POSSESSION
YES	ME	FW	LT	OF	LOST POSSESSION
YES	LG	FW	RT	MD	SUCCESFULL
YES	ST	BW	LT	MO	CONTINUED POSSESSION
YES	LG	FW	LT	OF	LOST POSSESSION
YES	ST	FW	RT	CE	CONTINUED POSSESSION
YES	LG	BW	RT	CE	UNSUCCESFULL
YES	ME	FW	RT	DF	LOST POSSESSION
YES	ME	BW	LT	CE	CONTINUED POSSESSION
YES	ME	BW	RT	MD	CONTINUED POSSESSION
YES	ST	BW	RT	MO	CONTINUED POSSESSION
YES	ST	BW	RT	MO	CONTINUED POSSESSION
YES	ME	BW	RT	MO	CONTINUED POSSESSION
YES	ST	BW	RT	OF	SUCCESFULL
YES	ST	BW	LT	OF	CONTINUED POSSESSION
YES	LG	BW	RT	CE	CONTINUED POSSESSION
YES	ST	BW	RT	OF	CONTINUED POSSESSION

YES	ME	FW	LT	DF	SUCCESFULL
YES	ME	FW	RT	MD	CONTINUED POSSESSION
YES	ME	FW	RT	CE	LOST POSSESSION
YES	LG	BW	RT	CE	LOST POSSESSION
YES	ST	BW	RT	MD	CONTINUED POSSESSION
YES	ST	FW	LT	OF	LOST POSSESSION
YES	ST	BW	LT	CE	LOST POSSESSION
YES	ST	BW	LT	MO	CONTINUED POSSESSION
YES	ST	BW	RT	MD	CONTINUED POSSESSION
YES	ME	FW	RT	MO	LOST POSSESSION
YES	ST	BW	RT	MO	UNSUCCESFULL
YES	ST	BW	RT	MO	CONTINUED POSSESSION
YES	ST	FW	LT	MO	CONTINUED POSSESSION
YES	ME	BW	RT	CE	CONTINUED POSSESSION
YES	ST	FW	LT	CE	UNSUCCESFULL
YES	ME	FW	RT	MO	LOST POSSESSION
YES	ST	FW	RT	DF	CONTINUED POSSESSION
YES	ME	FW	RT	MD	CONTINUED POSSESSION
YES	ST	BW	RT	MO	CONTINUED POSSESSION
YES	ME	FW	LT	MO	LOST POSSESSION
YES	ME	FW	LT	CE	CONTINUED POSSESSION
YES	ST	FW	LT	OF	LOST POSSESSION
YES	ST	FW	RT	MO	LOST POSSESSION
YES	LG	FW	LT	CE	CONTINUED POSSESSION
YES	ST	FW	LT	OF	LOST POSSESSION
YES	ME	FW	RT	MD	CONTINUED POSSESSION
YES	ME	FW	RT	MD	CONTINUED POSSESSION
YES	ST	FW	LT	CE	SUCCESFULL
YES	ST	FW	RT	MD	CONTINUED POSSESSION
YES	ST	FW	LT	MD	CONTINUED POSSESSION
YES	ME	BW	RT	OF	CONTINUED POSSESSION
YES	ST	FW	RT	MD	LOST POSSESSION
YES	ST	FW	LT	OF	CONTINUED POSSESSION
YES	LG	FW	LT	DF	LOST POSSESSION
NO	ME	BW	LT	CE	CONTINUED POSSESSION
YES	ST	BW	LT	MO	CONTINUED POSSESSION
YES	ST	FW	RT	OF	LOST POSSESSION
YES	ST	BW	RT	CE	CONTINUED POSSESSION
YES	ME	FW	LT	MD	LOST POSSESSION
YES	ME	FW	RT	MD	CONTINUED POSSESSION
YES	ST	BW	LT	MD	CONTINUED POSSESSION
YES	ME	FW	RT	MD	LOST POSSESSION
YES	ST	BW	RT	MO	CONTINUED POSSESSION
YES	ST	FW	LT	MD	UNSUCCESFULL
NO	ME	BW	RT	CE	CONTINUED POSSESSION
YES	ST	FW	LT	MO	CONTINUED POSSESSION
YES	ST	FW	RT	OF	SUCCESFULL
YES	ME	FW	LT	CE	CONTINUED POSSESSION
YES	ST	FW	LT	MO	LOST POSSESSION
YES	ST	BW	LT	MO	CONTINUED POSSESSION

YES	ST	FW	LT	OF	CONTINUED POSSESSION
NO	ME	BW	LT	OF	CONTINUED POSSESSION
YES	ST	FW	RT	OF	SUCCESFULL
YES	ME	FW	LT	MD	LOST POSSESSION
YES	ST	BW	RT	MD	CONTINUED POSSESSION
YES	ST	BW	RT	OF	CONTINUED POSSESSION
YES	ST	BW	RT	MO	LOST POSSESSION
YES	ST	FW	LT	MD	LOST POSSESSION
YES	ST	BW	LT	OF	LOST POSSESSION
YES	ST	BW	RT	DF	CONTINUED POSSESSION
NO	ST	BW	LT	MD	CONTINUED POSSESSION
YES	ME	FW	LT	OF	LOST POSSESSION
YES	ST	FW	RT	CE	CONTINUED POSSESSION
YES	ME	FW	RT	MO	CONTINUED POSSESSION
YES	ME	FW	RT	OF	CONTINUED POSSESSION
YES	ME	FW	RT	OF	SUCCESFULL
YES	ST	BW	RT	OF	SUCCESFULL
YES	ME	FW	RT	MD	CONTINUED POSSESSION
YES	ME	FW	RT	MD	LOST POSSESSION
YES	ST	BW	LT	MO	CONTINUED POSSESSION
YES	ME	FW	RT	OF	SUCCESFULL
NO	ST	BW	LT	MD	CONTINUED POSSESSION
YES	ST	BW	RT	CE	CONTINUED POSSESSION
YES	ME	FW	RT	CE	CONTINUED POSSESSION
YES	ME	BW	RT	MO	SUCCESFULL
NO	ST	BW	LT	CE	LOST POSSESSION
YES	ME	FW	RT	MD	LOST POSSESSION
YES	ST	BW	RT	MO	CONTINUED POSSESSION
YES	ME	FW	LT	MO	LOST POSSESSION
YES	LG	FW	RT	MD	LOST POSSESSION
YES	ME	BW	LT	CE	CONTINUED POSSESSION
YES	LG	FW	RT	OF	LOST POSSESSION
YES	ST	FW	RT	OF	CONTINUED POSSESSION
YES	ME	FW	RT	OF	CONTINUED POSSESSION
YES	ME	BW	RT	MO	CONTINUED POSSESSION
YES	ME	BW	LT	CE	CONTINUED POSSESSION
YES	ME	BW	RT	MO	CONTINUED POSSESSION
YES	ME	BW	RT	MO	CONTINUED POSSESSION
YES	ME	BW	RT	MO	CONTINUED POSSESSION
YES	ME	BW	RT	MO	CONTINUED POSSESSION
YES	ST	FW	RT	MO	CONTINUED POSSESSION
YES	LG	FW	LT	MD	CONTINUED POSSESSION
YES	LG	BW	RT	MD	LOST POSSESSION
YES	ST	FW	RT	MO	CONTINUED POSSESSION
YES	ST	BW	RT	MO	CONTINUED POSSESSION
YES	ME	FW	RT	OF	CONTINUED POSSESSION
YES	ME	FW	RT	MO	CONTINUED POSSESSION
YES	ME	BW	LT	MO	LOST POSSESSION
YES	ME	FW	LT	MO	SUCCESFULL
YES	ME	BW	LT	CE	CONTINUED POSSESSION

YES	ME	BW	RT	MO	CONTINUED POSSESSION
YES	LG	FW	LT	MO	LOST POSSESSION
YES	ST	FW	RT	MD	CONTINUED POSSESSION
YES	LG	FW	LT	MD	LOST POSSESSION
YES	LG	FW	RT	CE	LOST POSSESSION
YES	ST	BW	RT	MO	LOST POSSESSION
YES	LG	FW	RT	MD	CONTINUED POSSESSION
YES	ST	FW	RT	MO	CONTINUED POSSESSION
YES	LG	FW	LT	MO	CONTINUED POSSESSION
YES	ST	BW	LT	OF	CONTINUED POSSESSION
YES	ME	FW	LT	OF	LOST POSSESSION
YES	ST	FW	RT	MO	LOST POSSESSION
YES	ST	FW	RT	DF	LOST POSSESSION
YES	ST	BW	RT	DF	LOST POSSESSION
YES	ST	FW	RT	MO	LOST POSSESSION
YES	ME	FW	LT	OF	CONTINUED POSSESSION
YES	LG	FW	RT	MD	LOST POSSESSION
YES	LG	FW	LT	MO	LOST POSSESSION
YES	ME	FW	LT	MO	CONTINUED POSSESSION
YES	ST	FW	LT	MO	LOST POSSESSION
YES	ME	FW	RT	CE	CONTINUED POSSESSION
YES	ME	BW	LT	OF	CONTINUED POSSESSION
YES	ME	BW	LT	CE	CONTINUED POSSESSION
YES	ST	BW	RT	CE	CONTINUED POSSESSION
YES	ST	FW	RT	CE	CONTINUED POSSESSION
YES	ME	BW	LT	OF	CONTINUED POSSESSION
YES	ME	FW	RT	MO	CONTINUED POSSESSION
YES	ME	BW	LT	MO	CONTINUED POSSESSION
YES	ST	FW	RT	DF	LOST POSSESSION
YES	ST	BW	LT	MO	CONTINUED POSSESSION
YES	ME	FW	LT	MO	SUCCESFULL
YES	ME	FW	RT	MD	LOST POSSESSION
YES	ME	FW	RT	CE	CONTINUED POSSESSION
YES	ST	FW	LT	MO	LOST POSSESSION
YES	ST	BW	LT	MO	UNSUCCESFULL
YES	ME	BW	LT	CE	CONTINUED POSSESSION
YES	ME	BW	LT	OF	LOST POSSESSION
YES	ME	BW	LT	MD	LOST POSSESSION
YES	LG	FW	LT	MD	LOST POSSESSION
YES	LG	BW	LT	OF	CONTINUED POSSESSION
YES	LG	FW	LT	MO	LOST POSSESSION
YES	ME	BW	RT	MD	CONTINUED POSSESSION
YES	ME	BW	RT	CE	CONTINUED POSSESSION
YES	ME	FW	RT	MD	CONTINUED POSSESSION
YES	LG	BW	RT	OF	UNSUCCESFULL
YES	ST	FW	RT	OF	CONTINUED POSSESSION
YES	ME	FW	RT	MO	CONTINUED POSSESSION
YES	ME	BW	LT	CE	CONTINUED POSSESSION
YES	ME	FW	LT	MD	LOST POSSESSION
YES	ME	FW	LT	MD	LOST POSSESSION

YES	ME	FW	LT	MD	LOST POSSESSION
YES	ME	FW	LT	MO	SUCCESFULL
YES	ME	FW	RT	MO	CONTINUED POSSESSION
YES	LG	FW	LT	MO	CONTINUED POSSESSION
YES	ME	BW	LT	MO	CONTINUED POSSESSION
YES	LG	FW	LT	DF	LOST POSSESSION
YES	ST	FW	RT	MD	LOST POSSESSION
YES	ME	BW	RT	MO	CONTINUED POSSESSION
YES	ME	FW	RT	CE	LOST POSSESSION
YES	LG	FW	LT	MD	LOST POSSESSION
YES	LG	FW	LT	MD	CONTINUED POSSESSION
YES	LG	FW	LT	CE	CONTINUED POSSESSION
YES	LG	FW	LT	CE	LOST POSSESSION
YES	ME	FW	LT	MO	CONTINUED POSSESSION
YES	ME	BW	LT	OF	CONTINUED POSSESSION
YES	ME	FW	LT	MO	LOST POSSESSION
YES	ME	BW	RT	OF	LOST POSSESSION
YES	ME	BW	RT	MD	CONTINUED POSSESSION
YES	ME	FW	LT	MO	LOST POSSESSION
YES	ME	BW	LT	OF	CONTINUED POSSESSION
YES	ME	BW	LT	OF	CONTINUED POSSESSION
YES	ME	FW	LT	MO	LOST POSSESSION
YES	ME	FW	LT	CE	LOST POSSESSION
YES	LG	FW	LT	OF	LOST POSSESSION
YES	ME	BW	RT	MD	CONTINUED POSSESSION
YES	ME	BW	LT	MD	UNSUCCESFULL
YES	ST	FW	LT	DF	CONTINUED POSSESSION
YES	LG	BW	LT	MO	CONTINUED POSSESSION
YES	LG	FW	RT	OF	SUCCESFULL
YES	ME	BW	RT	CE	CONTINUED POSSESSION
YES	ME	BW	RT	MD	CONTINUED POSSESSION
YES	ME	BW	RT	CE	CONTINUED POSSESSION
YES	ME	BW	RT	MD	LOST POSSESSION
YES	ME	FW	LT	MD	CONTINUED POSSESSION
YES	ME	BW	RT	CE	CONTINUED POSSESSION
YES	ME	BW	RT	OF	LOST POSSESSION
YES	LG	BW	LT	CE	CONTINUED POSSESSION
YES	ST	BW	RT	MO	SUCCESFULL
NO	LG	BW	LT	CE	LOST POSSESSION
YES	ME	BW	RT	MO	CONTINUED POSSESSION
YES	ST	FW	RT	OF	SUCCESFULL
YES	ME	FW	RT	MO	CONTINUED POSSESSION
YES	ME	BW	LT	MO	LOST POSSESSION
YES	LG	BW	LT	OF	CONTINUED POSSESSION
YES	ST	BW	RT	MO	CONTINUED POSSESSION
YES	LG	BW	LT	MO	CONTINUED POSSESSION
YES	ST	BW	LT	OF	LOST POSSESSION
YES	ST	FW	LT	DF	UNSUCCESFULL
YES	ME	BW	RT	MO	CONTINUED POSSESSION
YES	ST	FW	LT	CE	CONTINUED POSSESSION

YES	ME	BW	RT	CE	LOST POSSESSION
NO	ME	BW	RT	MO	CONTINUED POSSESSION
YES	ST	FW	RT	CE	LOST POSSESSION
YES	ST	FW	RT	MD	SUCCESFULL
YES	ST	BW	LT	OF	CONTINUED POSSESSION
YES	ST	FW	LT	MO	CONTINUED POSSESSION
YES	LG	BW	RT	OF	CONTINUED POSSESSION
YES	ST	FW	LT	MD	LOST POSSESSION
YES	ST	FW	RT	MO	CONTINUED POSSESSION
YES	ST	FW	LT	MD	LOST POSSESSION
YES	ME	FW	LT	CE	LOST POSSESSION
YES	ST	BW	LT	OF	LOST POSSESSION
YES	ME	FW	LT	MD	CONTINUED POSSESSION
YES	ME	FW	LT	MO	CONTINUED POSSESSION
YES	ME	FW	LT	MO	CONTINUED POSSESSION
YES	ME	FW	LT	MO	LOST POSSESSION
YES	ME	FW	RT	OF	LOST POSSESSION
YES	ST	FW	RT	MD	CONTINUED POSSESSION
YES	ME	FW	LT	MD	LOST POSSESSION
YES	ME	BW	RT	MO	CONTINUED POSSESSION
YES	ME	FW	LT	MO	LOST POSSESSION
YES	ST	FW	LT	CE	CONTINUED POSSESSION
YES	ST	BW	RT	CE	CONTINUED POSSESSION
YES	ME	FW	RT	OF	LOST POSSESSION
YES	ME	FW	LT	MO	LOST POSSESSION
YES	ST	BW	RT	MO	LOST POSSESSION
YES	ST	FW	LT	CE	LOST POSSESSION
YES	ST	FW	LT	MO	CONTINUED POSSESSION
YES	ME	BW	RT	MO	CONTINUED POSSESSION
YES	ME	FW	RT	MO	SUCCESFULL
YES	ME	FW	RT	MO	CONTINUED POSSESSION
YES	ST	BW	LT	OF	CONTINUED POSSESSION
YES	ME	FW	LT	MD	LOST POSSESSION
YES	ST	FW	RT	MD	LOST POSSESSION
YES	ST	FW	RT	MO	LOST POSSESSION
YES	ME	FW	RT	CE	CONTINUED POSSESSION
YES	ME	FW	LT	MD	LOST POSSESSION
YES	LG	BW	RT	CE	CONTINUED POSSESSION
YES	ST	FW	RT	MO	CONTINUED POSSESSION
YES	ST	BW	LT	MO	LOST POSSESSION
YES	ME	FW	RT	MD	CONTINUED POSSESSION
YES	ME	FW	RT	CE	LOST POSSESSION
YES	ST	FW	LT	MD	CONTINUED POSSESSION
YES	ME	FW	RT	MD	LOST POSSESSION
YES	ME	BW	RT	MO	SUCCESFULL
YES	ST	FW	RT	CE	CONTINUED POSSESSION
YES	ST	FW	RT	MO	CONTINUED POSSESSION
YES	ST	FW	RT	OF	CONTINUED POSSESSION
YES	ST	BW	RT	MO	CONTINUED POSSESSION
YES	ME	FW	RT	MO	SUCCESFULL

YES	ST	BW	LT	MO	LOST POSSESSION
YES	ME	FW	LT	MO	LOST POSSESSION
YES	LG	BW	LT	MO	SUCCESFULL
YES	ST	FW	RT	MD	LOST POSSESSION
YES	ST	FW	LT	MD	LOST POSSESSION
YES	ME	BW	LT	CE	CONTINUED POSSESSION
YES	ST	BW	LT	OF	CONTINUED POSSESSION
YES	ME	FW	RT	MD	LOST POSSESSION
YES	ME	FW	RT	MO	LOST POSSESSION
YES	ME	FW	LT	MO	LOST POSSESSION
YES	ME	BW	RT	MO	LOST POSSESSION
YES	ME	FW	RT	MO	LOST POSSESSION
YES	ST	BW	RT	MO	LOST POSSESSION
YES	ST	FW	LT	CE	CONTINUED POSSESSION
YES	ST	FW	RT	CE	SUCCESFULL
YES	ME	BW	RT	CE	LOST POSSESSION
NO	ME	FW	LT	OF	LOST POSSESSION
YES	ST	FW	RT	DF	LOST POSSESSION
YES	ME	FW	LT	MO	CONTINUED POSSESSION
YES	ST	FW	LT	CE	CONTINUED POSSESSION
YES	ST	FW	LT	MD	CONTINUED POSSESSION
YES	ST	FW	RT	MO	LOST POSSESSION
YES	ST	BW	RT	CE	CONTINUED POSSESSION
YES	ME	FW	RT	CE	LOST POSSESSION
YES	ST	BW	LT	OF	CONTINUED POSSESSION
YES	ST	FW	RT	MD	CONTINUED POSSESSION
YES	ST	BW	RT	MO	LOST POSSESSION
YES	ST	FW	RT	MO	CONTINUED POSSESSION
YES	ST	BW	RT	MO	UNSUCCESFULL
YES	ST	FW	RT	MD	LOST POSSESSION
YES	ME	FW	RT	CE	LOST POSSESSION
YES	ST	BW	LT	MO	LOST POSSESSION
YES	ME	FW	LT	MO	LOST POSSESSION
YES	ME	BW	RT	MO	CONTINUED POSSESSION
YES	ME	FW	LT	MD	CONTINUED POSSESSION
YES	ME	FW	LT	MD	LOST POSSESSION
YES	ME	FW	LT	CE	CONTINUED POSSESSION
YES	ME	FW	LT	MD	LOST POSSESSION
YES	ST	FW	RT	CE	CONTINUED POSSESSION
YES	ST	FW	LT	CE	LOST POSSESSION
YES	ST	FW	RT	CE	CONTINUED POSSESSION
YES	ST	FW	RT	MO	CONTINUED POSSESSION
YES	ME	BW	LT	MO	CONTINUED POSSESSION
YES	ME	FW	LT	DF	LOST POSSESSION
YES	ST	BW	LT	MO	CONTINUED POSSESSION
YES	ST	FW	RT	MO	CONTINUED POSSESSION
YES	ST	BW	RT	CE	CONTINUED POSSESSION
YES	ME	BW	RT	MO	CONTINUED POSSESSION
YES	ST	FW	LT	CE	CONTINUED POSSESSION
YES	ST	FW	LT	MO	CONTINUED POSSESSION

YES	ST	BW	LT	MO	CONTINUED POSSESSION
NO	ME	BW	LT	MO	CONTINUED POSSESSION
YES	ST	BW	LT	CE	CONTINUED POSSESSION
YES	ME	BW	LT	MO	CONTINUED POSSESSION
YES	ST	FW	RT	MO	CONTINUED POSSESSION
YES	ST	BW	LT	OF	LOST POSSESSION
YES	ST	FW	RT	OF	CONTINUED POSSESSION
YES	ST	BW	LT	MO	CONTINUED POSSESSION
YES	ST	FW	RT	MD	CONTINUED POSSESSION
YES	ST	BW	RT	OF	CONTINUED POSSESSION
YES	ST	FW	RT	CE	LOST POSSESSION
YES	ST	FW	RT	CE	CONTINUED POSSESSION
YES	ST	FW	RT	MO	LOST POSSESSION
YES	ST	FW	RT	CE	LOST POSSESSION
YES	ME	BW	LT	OF	CONTINUED POSSESSION
YES	ST	FW	RT	CE	LOST POSSESSION
YES	ST	FW	LT	MO	LOST POSSESSION
YES	ME	FW	LT	CE	LOST POSSESSION
YES	ME	FW	LT	MD	SUCCESFULL
YES	ME	FW	LT	CE	CONTINUED POSSESSION
YES	ST	FW	LT	CE	LOST POSSESSION
YES	ME	FW	RT	MD	CONTINUED POSSESSION
YES	ME	FW	RT	MO	CONTINUED POSSESSION
YES	ST	FW	RT	MO	LOST POSSESSION
YES	ME	FW	LT	MO	CONTINUED POSSESSION
YES	ME	FW	LT	MO	LOST POSSESSION
YES	ST	BW	RT	MO	LOST POSSESSION
YES	ME	FW	RT	MD	LOST POSSESSION
YES	ME	FW	RT	CE	SUCCESFULL
YES	ME	FW	LT	MD	CONTINUED POSSESSION
YES	ST	FW	LT	CE	CONTINUED POSSESSION
YES	ME	FW	LT	CE	SUCCESFULL
YES	ST	FW	LT	OF	LOST POSSESSION
YES	ME	FW	RT	CE	CONTINUED POSSESSION
YES	ME	FW	RT	CE	LOST POSSESSION
YES	ME	FW	LT	MO	LOST POSSESSION
YES	ME	FW	LT	MD	LOST POSSESSION
YES	ST	FW	LT	DF	LOST POSSESSION
YES	ST	FW	LT	DF	LOST POSSESSION
YES	ME	FW	LT	MO	CONTINUED POSSESSION
YES	ST	BW	LT	OF	LOST POSSESSION
YES	ST	FW	LT	MO	CONTINUED POSSESSION
YES	ST	BW	RT	MD	CONTINUED POSSESSION
YES	ST	BW	LT	MD	LOST POSSESSION
YES	ME	FW	LT	CE	CONTINUED POSSESSION
YES	ME	FW	RT	MO	LOST POSSESSION
YES	ME	BW	RT	OF	LOST POSSESSION
YES	ME	FW	LT	MO	CONTINUED POSSESSION
YES	ST	FW	LT	MO	LOST POSSESSION
YES	ME	FW	LT	CE	LOST POSSESSION

YES	ME	FW	RT	DF	LOST POSSESSION
YES	ST	FW	LT	OF	LOST POSSESSION
YES	ST	BW	LT	MO	LOST POSSESSION
YES	ME	FW	LT	MO	LOST POSSESSION
YES	ME	FW	RT	MO	CONTINUED POSSESSION
YES	ME	FW	LT	MO	LOST POSSESSION
YES	ME	FW	RT	MO	LOST POSSESSION
YES	ME	FW	RT	DF	CONTINUED POSSESSION
YES	ST	FW	LT	MD	CONTINUED POSSESSION
YES	ME	FW	LT	MD	CONTINUED POSSESSION
YES	ME	FW	LT	MD	CONTINUED POSSESSION
YES	ME	FW	RT	MD	CONTINUED POSSESSION
YES	ME	FW	RT	MD	CONTINUED POSSESSION
YES	ST	FW	LT	MD	CONTINUED POSSESSION
YES	ME	FW	LT	MO	SUCCESFULL
YES	ME	FW	RT	CE	CONTINUED POSSESSION
YES	ME	FW	LT	MO	CONTINUED POSSESSION
YES	ST	FW	LT	MO	CONTINUED POSSESSION
YES	ST	FW	RT	MD	CONTINUED POSSESSION
YES	ST	FW	LT	MD	CONTINUED POSSESSION
YES	ST	FW	LT	OF	SUCCESFULL
YES	ST	FW	RT	MD	CONTINUED POSSESSION
YES	ST	BW	LT	OF	SUCCESFULL
YES	ST	FW	LT	MD	CONTINUED POSSESSION
YES	ST	BW	LT	DF	CONTINUED POSSESSION
YES	ST	BW	RT	CE	CONTINUED POSSESSION
YES	ST	FW	LT	MD	UNSUCCESFULL
YES	ST	FW	RT	CE	CONTINUED POSSESSION
YES	ME	FW	LT	MO	CONTINUED POSSESSION
YES	ST	FW	RT	CE	LOST POSSESSION
YES	ST	FW	RT	MD	CONTINUED POSSESSION
YES	ST	FW	RT	MD	CONTINUED POSSESSION
YES	ST	FW	LT	OF	CONTINUED POSSESSION
NO	ME	BW	LT	MD	CONTINUED POSSESSION
YES	ST	FW	RT	OF	SUCCESFULL
YES	ST	FW	LT	OF	CONTINUED POSSESSION
YES	ST	FW	LT	OF	CONTINUED POSSESSION
YES	ST	FW	RT	MO	CONTINUED POSSESSION
YES	ST	FW	RT	OF	CONTINUED POSSESSION
YES	ST	FW	LT	MO	CONTINUED POSSESSION
YES	ME	FW	RT	MO	LOST POSSESSION
YES	ME	FW	RT	MO	CONTINUED POSSESSION
YES	ST	BW	RT	MO	CONTINUED POSSESSION
YES	ME	FW	RT	MO	SUCCESFULL
YES	ME	FW	RT	MO	CONTINUED POSSESSION
YES	ST	FW	RT	MO	CONTINUED POSSESSION
YES	ST	FW	RT	CE	CONTINUED POSSESSION
YES	LG	FW	LT	MO	LOST POSSESSION
YES	LG	FW	RT	CE	LOST POSSESSION
YES	ME	FW	RT	CE	CONTINUED POSSESSION

YES	ME	FW	RT	CE	CONTINUED POSSESSION
YES	LG	FW	RT	MD	CONTINUED POSSESSION
YES	LG	FW	LT	MO	LOST POSSESSION
YES	LG	FW	RT	MD	LOST POSSESSION
YES	ST	BW	LT	MD	CONTINUED POSSESSION
YES	ST	FW	RT	MO	UNSUCCESFULL
YES	ME	FW	LT	CE	LOST POSSESSION
YES	ST	FW	LT	MO	LOST POSSESSION
